# Diversity and function of the Antarctic krill microorganisms from *Euphausia superba*

**DOI:** 10.1038/srep36496

**Published:** 2016-11-04

**Authors:** Xiaoqiu Cui, Guoliang Zhu, Haishan Liu, Guoliang Jiang, Yi Wang, Weiming Zhu

**Affiliations:** 1 Key Laboratory of Marine Drugs, Ministry of Education of China, School of Medicine and Pharmacy, Ocean University of China, Qingdao 266003, China; 2 School of Pharmacy, Jining Medical University, Jining 272067, China; 3 College of Marine Life, Ocean University of China, Qingdao 266003, China

## Abstract

The diversity and ecological function of microorganisms associated with *Euphausia superba*, still remain unknown. This study identified 75 microbial isolates from *E*. *superba*, that is 42 fungi and 33 bacteria including eight actinobacteria. And all the isolates showed NaF tolerance in conformity with the nature of the fluoride krill. The maximum concentration was 10%, 3% and 0.5% NaF for actinobacteria, bacteria and fungi, respectively. The results demonstrated that 82.4% bacteria, 81.3% actinobacteria and 12.3% fungi produced antibacterial metabolites against pathogenic bacteria without NaF; the MIC value reached to 3.9 μg/mL. In addition, more than 60% fungi produced cytotoxic metabolites against A549, MCF-7 or K562 cell lines. The presence of NaF led to a reduction in the producing antimicrobial compounds, but stimulated the production of cytotoxic compounds. Furthermore, seven cytotoxic compounds were identified from the metabolites of *Penicillium citrinum* OUCMDZ4136 under 0.5% NaF, with the IC_50_ values of 3.6–13.1 μM for MCF-7, 2.2–19.8 μM for A549 and 5.4–15.4 μM for K562, respectively. These results indicated that the krill microbes exert their chemical defense by producing cytotoxic compounds to the mammalians and antibacterial compounds to inhibiting the pathogenic bacteria.

Diverse microorganisms have been acting in a state of commensalism or parasitism with animals and plants for thousands of years^1^. Mutualistic relationships are abundant in the nutrient cycle, and enforce the protection between microorganisms and the host against environmental conditions^2^. The Antarctic region is considered a unique and severe ecological environment, dominated by microorganisms^3^. The Antarctic krill, *Euphausia superba*, is the staple food source for whales, fishes and seals, playing a vital role in the Antarctic Ocean ecosystem^4^. Previous research on Antarctic krill has focused on the distribution, the fishing demand and conservation measures^5^. Consequently, few studies have investigated the molecular biology and chemistry of the mutualism relationship between Antarctic krill and its associate microbes. Bacteria growth occurs on the surface and stomach of krill, and is an important component of the total digestive process in *E*. *superba*^6^,^7^. A psychrotrophic halotolerant, *Psychrobacter proteolyticus* sp. 116, was isolated and identified from the stomach of the Antarctic krill^8^. However, there are no reports on phylogenetic and bioactive microorganisms from *E*. *superba*. Additionally, the function of the microorganisms to *E*. *superba* has yet to be investigated. Thus, we investigated the diversity of microbial community, the analysis of phylogenetic relationships and the chemical defenses provided to their host of microorganisms associated with *E*. *superba* collected in the Antarctic area FAO48.1. The chemical defenses of the microbes from *E*. *superba* to their host were represented as the inhibitions on aquatic and human pathogenic bacteria as well as the cytotoxicity to mammal cells.

In addition, the impacts of the abnormally high levels of fluoride contents in *E. superba*, due to *E. superba* concentrating fluoride from the seawater, was investigated^9^. Although high concentrations of fluoride are toxic to other animals, it is an important component of krill cuticle formation^10^. The fluoride content varies in different part of *E. superba*; the fresh shell has levels approximately five times higher (13000 μg/g) than the meat^11^. The biological availability of fluoride in *E. superba* allows the utilization of sodium fluoride (NaF)^12^. Thus, commensalism was also studied by investigating the tolerance of the microbial community to NaF.

## Results

### Phylogenetic Diversity of Culturable strains Associated with Euphausia superba

**Figure 1:**
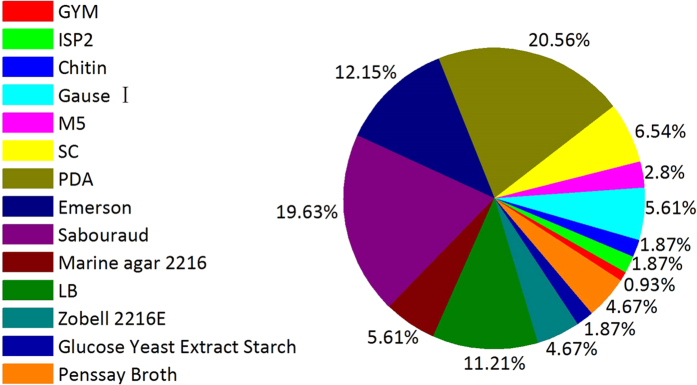
Percentage of microbial isolates associated with *E*. *superba* from the different media.

Using thirteen culture media (Table 1), 134 microbial isolates were obtained from *E*. *superba*. The duplicated strains were removed using a detailed morphological approach, pigment formation and HPLC fingerprint analysis of the extracts. The 75 representative isolates, including 42 fungal strains, 33 bacterial strains among which eight strains belonged to actinobacteria, were selected for sequencing and identified successfully (Table 2). Using gene sequences and phylogenetic analysis, these 75 representative strains were classified into eleven genera, nine families, and seven orders within seven classes of four phyla; most sequence showed from 98% to 100% homology (Table 2, Figs S1–S75). The most suitable medium for isolating fungi, actinobacteria and bacteria from *E*. *superba* was PDA, M5 and LB medium, respectively (Fig. 1). The optimum temperature for isolating bacteria, actinobacteria and fungi was 16 °C, 16 °C and 28 °C, respectively. Among 25 bacterial isolates, 23 bacterial isolates were obtained at 16 °C and only two isolates, *Streptomyces* sp. OUCMDZ4220 and *Advenella* sp. OUCMDZ4222, were obtained at 4 °C. Eight actinobacterial isolates were obtained at 16 °C, while 42 fungal isolates were obtained at 28 °C.

**Figure 2:**
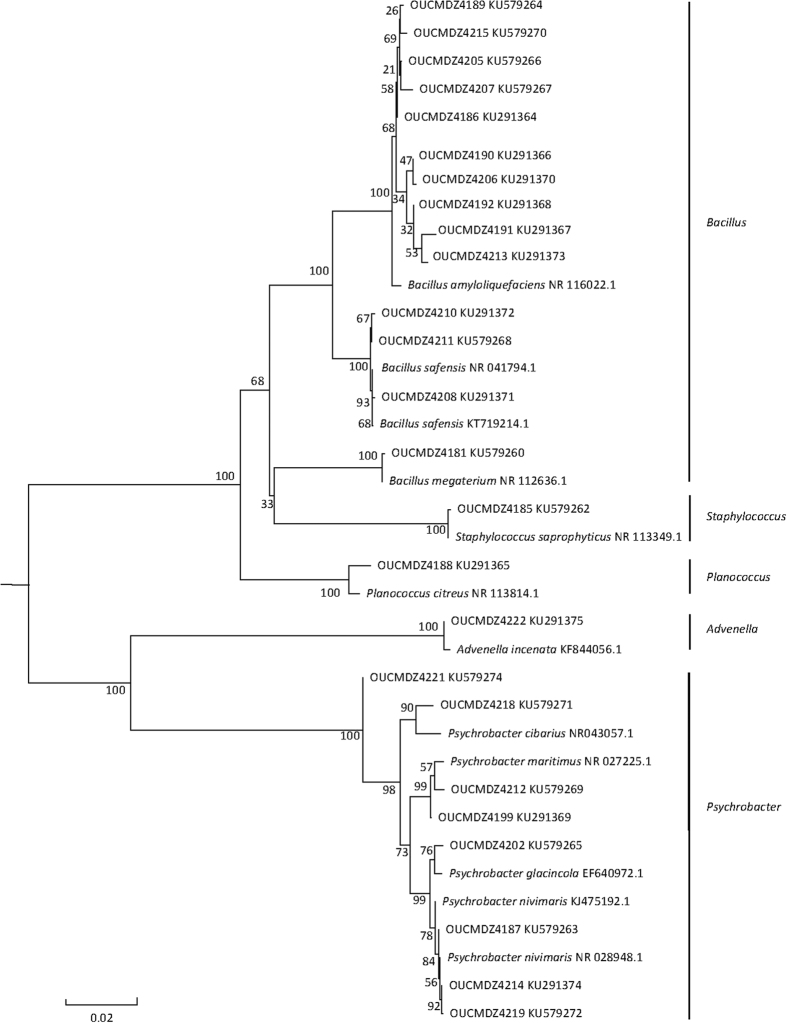
Neighbor-joining of phylogenetic tree of 25 representative bacteria isolates from *E. superba* based on 16S rRNA gene sequences. The numbers on each node represent the bootstrap value from 1000 replicates and the scale bar represents 0.02 substitutions per nucleotide.

**Figure 3:**
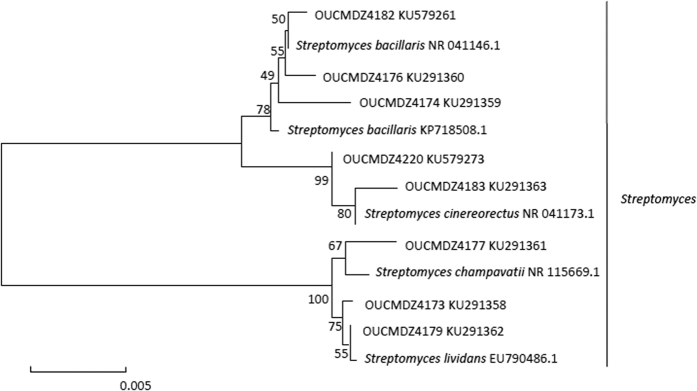
Neighbor-joining of phylogenetic tree of eight representative actinobacterial isolates from *E*. *superba* based on 16S rRNA gene sequences. The numbers on each node represent the bootstrap value from 1000 replicates and the scale bar represents 0.005 substitutions per nucleotide.

**Figure 4:**
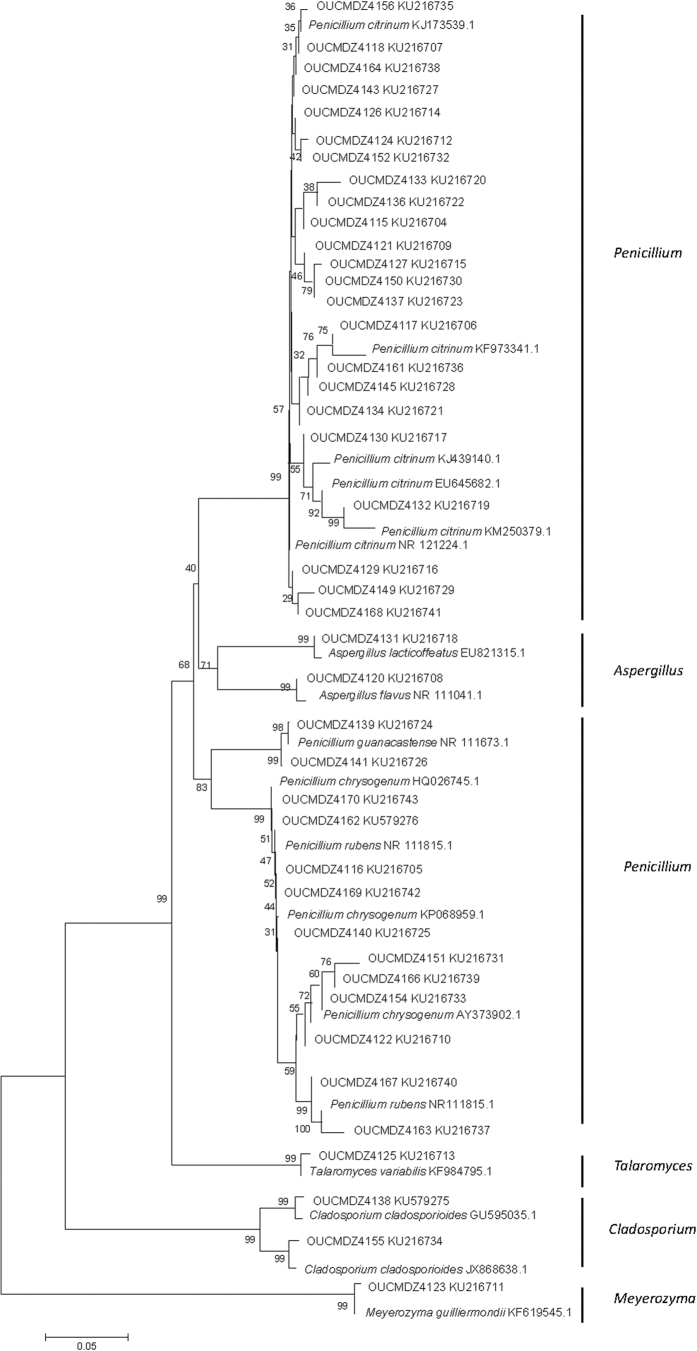
Neighbor-joining of phylogenetic tree of 42 representative fungi isolates from *E*. superba. based on 18S rRNA gene sequences. The numbers on each node represent the bootstrap value from 1000 replicates and the scale bar represents 0.05 substitutions per nucleotide

**Figure 5:**
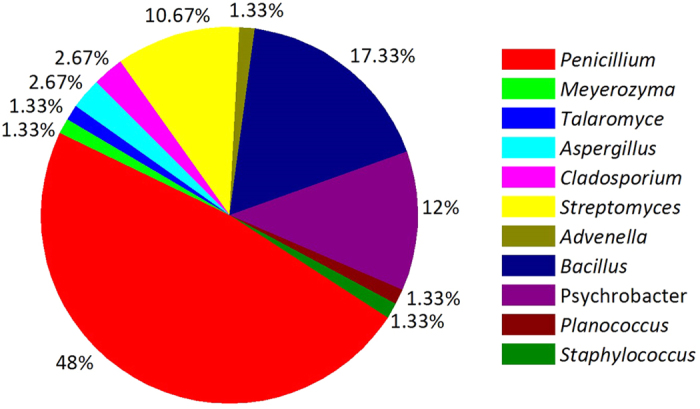
Diversity of microbial isolates from *E*. *superba*.

Genebank accession number and their blast results in the NCBI database of the identified strains were summarized (Tables 2 and 3). Based on the homology of 16S or 18S rRNA gene, three phylogenetic trees containing all the isolates were constructed to analyze the phylogenetic relationship of microorganisms (Figs 2, 3 and 4). The taxonomical group classification concluded 42 fungal strains, eight actinobacteria and 25 bacterial strains. The fungal isolates belonged to the phylum *Ascomycota*, including three classes (*Eurotiomycetes, Dothideomycetes and Saccharomycetes*), three orders (*Eurotiales, Capnodiales* and *Saccharomycetales*), three families (Trichocomaceae, Mycosphaerellaceae and Debaryomycetaceae) and five genera (*Penicillium, Cladosporium, Aspergillus*, *Talaromyces and Meyerozyma*) (Fig. 5). *Penicillium* was the dominant fungal group accounting for 85.7% of all isolates. The bacterial isolates included two phyla, *Firmicutes* and *Proteobacteria*. Fifteen isolates belonged to the phylum *Firmicutes*, including one class (*Bacilli*), one order (*Bacillales*), three families (Bacillaceae, Staphylococcaceae and Planococcaceae) and three genera (*Bacillus, Staphylococcus* and *Planococcus*); *Bacillus* was the major bacterial genera identified (Fig. 5). Ten pure cultures belonged to the *Proteobacteria* phylum including two classes (*Gammaproteobacteria* and *Betaproteobacteria*), two orders (*Pseudomonadales* and *Burkholderiales*), two families (Moraxellaceae and Alcaligenaceae) and two genera (*Psychrobacter* and *Advenella*). Actinobacteria community associated with *E*. *superba* composed of eight isolates belonging to one *Actinobacteria* phylum, one *Actinobacteria* class, one *Streptomycineae* order, one Streptomycetaceae family and one *Streptomyces* genus (Fig. 5). *Streptomyces* was the only isolated genus from *E*. *superba*. It should be noted that strains OUCMDZ4221 and OUCMDZ4222 were identified as actinobacteria initially because of their morphological features; however, the 16S rRNA gene sequences analysis indicated they belong to *Psychrobacter* and *Advenella*, respectively.

### The antibacterial activity

**Figure 6:**
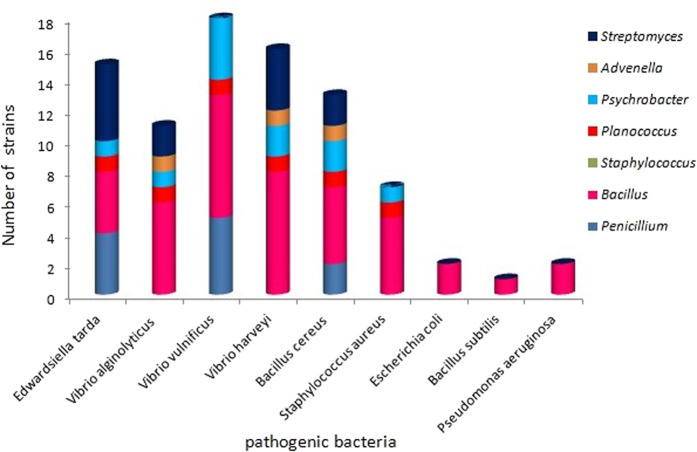
The antibacterial activity of different genera microbes from *E*. *superba*.

**Figure 7:**
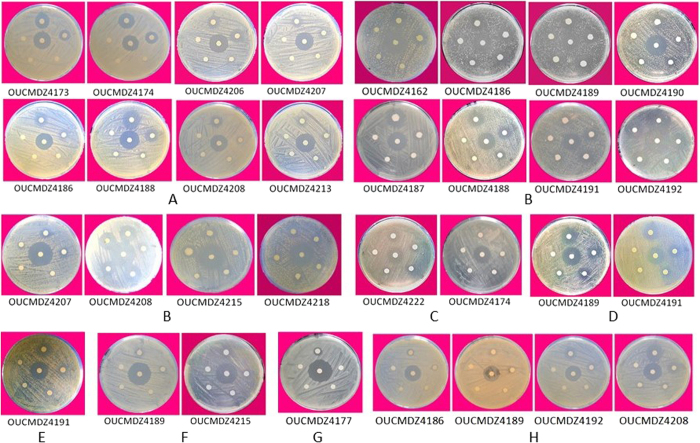
The MIC determination of active strains against aquatic and human pathogenic bacteria. The middle paper was absorbed the positive drug, and all around papers were absorbed 10 μL samples with different concentration. The numbers such as OUCMDZ4189 were the strain number. The sample concentrations of each paper absorbed clockwise from twelve o’clock corresponded to 1000, 500, 250, 125 and 62.5 μg/mL for A–G and 62.5, 31.25, 15.62, 7.81 and 3.90 μg/mL for H, respectively. (**A**) against *Edwardsiella tarda*; (**B**) against *Vibrio vulnificus*; (**C**) against *Vibrio harveyi*; (**D**) against *Vibrio alginolyticus;* (**E**) against *Staphylococcus aureu*; (**F**) against *Bacillus cereus*; G. against *S*. *aureus* in the actinobacterial medium 2# containing 8% NaF, H. against *V*. *vulnificus*)

All ethyl acetate (EtOAc) extracts of the 75 isolates were tested for antibacterial activity against six aquatic pathogenic bacteria (*Vibrio vulnificus*, *V*. *alginolyticus*, *V*. *parahemolyticus*, *V*. *harveyi*, *Edwardsiella tarda*, *Bacillus cereus*) and five human pathogenic bacteria (*Staphylococcus aureus*, *Escherichia coli*, *Enterobacter aerogenes*, *Bacillus subtilis* and *Pseudomonas aeruginosa*). The bacterial and actinobacterial metabolites from *E. superba* displayed broad and stronger antibacterial activity than fungal metabolites, and the antibacterial activity focused on inhibition of aquatic pathogenic bacteria, especially *V*. *vulnificus*, followed by *E*. *tarda*, *B*. *cereus*, and *V*. *alginolyticus* (Table 4, Figs 6 and 7). Among all the isolates, the genus bacteria of *Psychrobacter* and *Bacillus* displayed the best antibacterial activity. Three *Bacillus* sp. OUCMDZ4186, OUCMDZ4192 and OUCMDZ4208 and one *Psychrobacter* sp. OUCMDZ4189 were the most active, whose minimal inhibitory concentration (MIC) values against *V*. *vulnificus* reached to 31.2, 15.6, 7.8 and 3.9 μg/mL, respectively. The MIC values for *Bacillus* sp. OUCMDZ4191 against *B. cereus*, *V*. *alginolyticus* and *S*. *aureus*, and the *Planococcus* sp. OUCMDZ4188 against *V*. *vulnificus* and *E*. *tarda* were 62.5, 125 and 125, 125 and 250 μg/mL, respectively. The MIC values for *Psychrobacter* sp. OUCMDZ4189 against *B. cereus*, *V*. *alginolyticus* and *V*. *vulnificus*, and *Psychrobacter* sp. OUCMDZ4187 against *V*. *vulnificus* were 500, 62.5 and 31.2, and 125 μg/mL, respectively. *Streptomyces* sp. OUCMDZ4173 and OUCMDZ4174 were active on *E*. *tarda*, both with the MIC value of 250 μg/mL. Only a few fungi displayed weak antibacterial activity against *E*. *tarda, V*. *vulnificus* and *B. cereus* at the concentration of 1000 μg/mL, among which *Penicillium* sp. OUCMDZ4162 displayed strongest inhibition on *V*. *vulnificus* with MIC values of 250 μg/mL.

### The cytotoxicity

**Figure 8:**
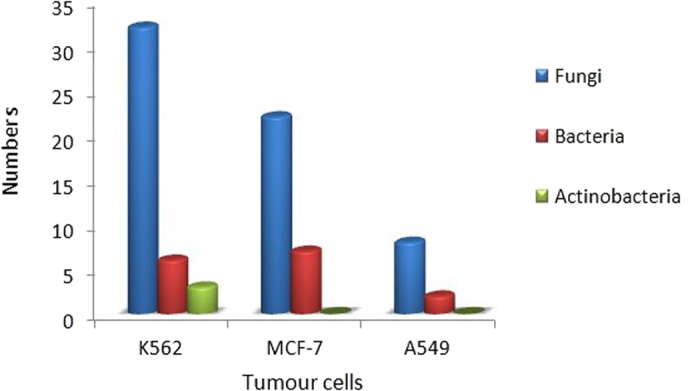
The quantities of active strains (≥ 60% inhibition at 100 μg/mL) from *E. superba* against K562, MCF-7 and A549 tumor cell lines.

All the EtOAc extracts were also evaluated their cytotoxicities against A549, K562 and MCF-7 tumour cell lines at the concentration of 100 *μ*g/mL. Most of the fungal metabolites displayed more than 60% inhibition against three cell lines, equivalent or near to the adriamycin (positive control); 75%, 72% and 65% inhibitions on the A549, K562 and MCF-7 cell lines, respectively, at the concentration of 1 μM were found for adriamycin (Table 5, Fig. 8). The active strain was defined as those with 60% inhibition at the 100 μg/mL. Additionally, results indicated that 83%, 69% and 19% of the fungal isolates were active against the K562, MCF-7 and A549 cell lines, respectively. However, the *Penicillium* sp. OUCMDZ4122 showed proliferative effects on A549, K562 and MCF-7 cell lines with −60.0%, −203.3% and −139.5% inhibitions, respectively. The ratios of bacteria to produce active metabolites against K562 were 24%, while 28% was found for the MCF-7 cell line. Very few bacteria produced the cytotoxic metabolites against A549 cell line. Only three actinobacteria produced cytotoxic metabolites to K562 cell line, and these metabolites were not active against A549 and MCF-7 cell lines.

### NaF tolerance of microbes and the corresponding bioactivity

In order to verify whether the microbial isolates were from the Antarctic krill, the NaF tolerances of all the 75 isolates were evaluated by adding different concentrations of NaF into the media. The maximum NaF tolerance concentration of actinobacteria, bacteria and fungi reached to 10% (in actinobacterial medium 2#), 3% (in LB medium) and 0.5% (in fungal medium 2#) (Tables 6, 7 and 8). The bioactive screening indicated that adding NaF had a significant effect on metabolites. After adding NaF into the media, the microbes reduced the production of antimicrobial products toward the tested pathogenic bacteria, the exception being *Streptomyces* sp. OUCMDZ4177, where metabolites were active at 1 mg/mL against *S*. *aureus* under the actinobacterial medium 2# containing 8% NaF (Fig. 7G). This is consistent with the fact that fluoride is toxic to microorganisms especially in high concentrations. Thus, the fluoride-tolerant microorganisms might reduce the yield of the anti-pathogenic bacterial compounds in such high NaF media. Although only one actinobacterial isolate produced the antibacterial metabolites under NaF, the cytotoxicity of the actinobacterial metabolites under NaF increased. For example, nearly all of the actinobacterial metabolites under 2%, 3%, 8% and 10% NaF displayed more than 50% inhibition on MCF-7 cells (Table 9). However, the secondary metabolites of bacteria under NaF did not show cytotoxicities. And the eight fungi produced the cytotoxic metabolites against three tumor cell lines under 0.5% NaF (Table 10). Among them, *Penicillium citrinum* OUCMDZ4136 was selected for further chemical study by producing the strongest cytotoxic metabolites against three tumor cells in the SWS medium (Table 1) containing 0.5% NaF. Chemical studies led to the identification of seven compounds (**1**–**7**). These compounds did not show antibacterial activity towards 11 pathogenic bacteria but showed pronounced cytotoxicity against MCF-7, A549 and K562 tumor cells with the IC_50_ values ranging from 2.2 to 19.8 μM (Table 11), further indicating that krill microbes continue to produce cytotoxic compounds under a fluoride media.

### Compounds from *Penicillium citrinum* OUCMDZ4136

**Figure 9:**
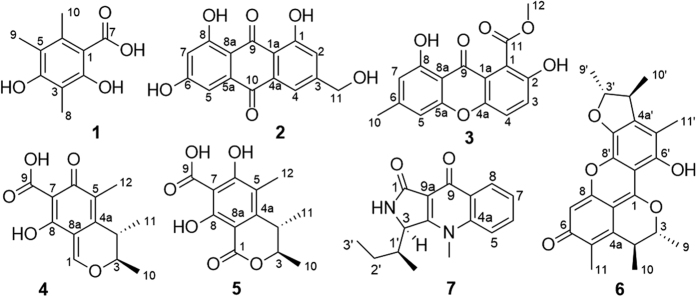
The chemical structures of the identified seven compounds.

Seven compounds were isolated from the fermentation broth of *P*. *citrinum* OUCMDZ4136 in the SWS medium containing 0.5% NaF. By comparison of NMR and specific rotation data with those reported, their structures were identified as 2,4-dihydroxy-3,5,6-trimethylbenzoic acid (**1**)^13^,^14^, citreorosein (**2**)^15^,^16^, pinselin (**3**)^17^, citrinin (**4**)^18^,^19^,^20^, dihydrocitrinone (**5**)^21^,^22^, pennicitrinone A (**6**)^23^,^24^ and quinolactacin A1 (**7**)^25^, respectively (Fig. 9).

## Discussion and Conclusion

Both pathogenic bacteria *Edwardsiella tarda* and *Vibrio vulnificus* are common, serious and even fatal aquatic pathogenic bacteria^26^,^27^. These infections seriously threaten aquatic agriculture and lead to serious ecological losses in the aquatic area. Pathogenic bacteria also have a wide host distribution in the Antarctic region^28^, typically causing intestinal disease and wound infections in many organisms including marine life and humans^29^,^30^,^31^. *E. superba* appears to have developed an immune system to avoid or restrain the invasion of aquatic pathogenic bacteria and other organisms. For example, Antarctic krill enzymes had significant effect on treating venous leg ulcer and could fast promote wound healing when compared with the non-enzymes control^32^. In addition, microorganisms from *E*. *superba* may participate in the chemical defense of the host through the production of antibacterial and/or cytotoxic metabolites. Our results indicated that the microbial isolates produced antibacterial metabolites against *E. tarda*, *V. vulnificus*, *V*. *harveyi*, *V*. *alginolyticus*, *B*. *cereus*, *S*. *aureus* and *E*. *coli* were 17, 17, 15, 9, 13, 8, and 2 strains, respectively. Additionally, the microbes that produced the cytotoxic metabolites with more than 60% inhibitions to K562, MCF-7 and A549 cell lines were 41, 29 and 10 strains, respectively. Even in the condition of the NaF, most of the actinobacteria and some fungi still produce cytotoxic products. For example, compounds **1**–**7** from the metabolites of *P. citrinum* OUCMDZ4136 under 0.5% NaF displayed moderate to strong cytotoxicity against A549, K562 and MCF-7 tumour cells. These results provide scientific evidence that microbes from *E*. *superba* participate in the protection of their host by producing antibacterial metabolites to pathogenic and cytotoxic metabolites for predatory mammals even under the high concentration of NaF.

In conclusion, a total 75 microbial isolates were identified from *E*. *superb*a collected in the Antarctic area FAO48.1. The phylogenetic relationships of these culturable microorganisms on the basis of gene sequences were constructed successfully. This construction revealed diverse krill-associated microbes including four phyla, seven classes, seven orders, nine families and 11 genera. All isolates were able to survive under NaF, and the NaF endurance ranged from 0.5% to 10%. These krill microbes could metabolize the antibacterial and cytotoxic products in the media without NaF, but mainly produced cytotoxic compounds under NaF. This study proves that symbiotic microbes may use their cytotoxic and antibacterial metabolites as the chemical weapon to protect their host in addition to receiving a nutritional benefit from the host.

## Methods

### General experimental procedures

The sample of the Antic krill strains were isolated from *Euphausia superba* collected from the Southern Ocean FAO48.1 (50–65°W, 50–65°S) (Fig. S77) in February 2010. The purified strains were submitted to the Identification Service of BGI tech (Beijing, China) for sequencing. The phylogenic relationships on the basis of gene sequences were analyzed. At the same time, the assay of NaF tolerance and anti-bacterial and cytotoxic activity were carried out. The aquatic pathogenic bacteria, *Edwardsiella tarda* (ATCC 15947), *Vibrio parahemolyticus* (ATCC 17802), *V*. *vulnificus* (ATCC 27562), *V*. *harveyi* (ATCC 14126), *V*. *alginolyticus* (ATCC 17749) and *Bacillus cereus* (ATCC 14579), the human pathogenic bacteria, *Staphylococcus aureus* (ATCC 6538), *Escherichia coli* (ATCC 11775), *Enterobacter aerogenes* (ATCC 13048), *Bacillus subtilis* (ATCC 6051) and *Pseudomonas aeruginosa* (ATCC 10145), were purchased from the Institute of Microbiology, Chinese Academy of Science, and Guangdong Institute of Microbiology. Nucleotide sequences were measured using an ABI3730XL sequencing apparatus. IR spectra were taken using a Nicolet NEXUS 470 spectrophotometer as KBr disks. UV spectrum data were obtained on a UH5300 spectrophotometer. Specific rotations were determined by a JASCO P-1020 digital polarimeter. CD data were obtained on a JASCO J-815 spectropolarimeter. ^1^H, ^13^C and DEPTQ NMR spectra of compounds **1**–**7** were recorded by Bruker Avance III 600 MHz spectrometer using TMS as internal standard, and chemical shifts were recorded as *δ* values that were further referenced to residual solvent signals for DMSO (*δ*_H_/*δ*_C_, 2.50/39.5). ESIMS data were recorded by a Q-TOF ULTIMA GLOBAL GAA076 LC mass spectrometer. TLC and column chromatography (CC) and vacuum-liquid chromatography (VLC) were performed on plates precoated with silica gel GF_254_ (10ica *μ*m) and silica gel (200cate mesh, Qingdao Marine Chemical Factory) and Sephadex LH-20 (Amersham Biosciences), respectively. HPLC and semi-preparative HPLC were performed using a Cholester column (YMC-pack, 4.6 × 250 mm, 5 *μ*m, 1 mL/min) and an ODS column [YMC-pack ODS-A, 10 × 250 mm, 5 μm, 4 mL/min], respectively.

### The isolations of Antarctic krill microbes

The *E*. *superba* (1.0 g) were leached with sterile seawater three times under sterile conditions to remove superficial sediments and microbes. The specimen was then homogenized and diluted with sterile seawater into three different concentrations (0.1, 0.01 and 0.001), 100 μL of each dilution were plated onto the corresponding isolation medium (Table 1) in triplicate. Chloramphenicol (100 μg/mL) was added into the medium to prohibit the growth of bacteria during the cultivation of actinobacteria and fungi. The inoculated plates of actinobacteria and bacteria were propagated at 4 or 16 °C for 3–90 d, whereas the fungi isolates were cultured at 4, 16, 25 or 28 °C for at least 5 d. If the colony could not be observed at 4 °C during three months, the next temperature was selected till the colony was visible. All microorganisms of the single colony were isolated, then inoculated onto a slope media and stored in a 4 °C refrigerator. Additionally, the same strains were preserved in 20% glycerine liquid medium at −80 °C for the future use.

### Microbial identification and Phylogenetic Analysis

Genomic DNA extraction and PCR amplification were conducted by BGI (The Beijing Genomics Institute, www.genomics.org.cn). The purified PCR product was sequenced bidirectionally on an ABI 3703 automated sequenator. The sequences contigs were edited by Bioedit 7.0.9.0 and MEGA 6.06 (Molecular Evolutionary Genetics Analysis) software. The obtained sequences were deposited in the NCBI (National Center for Biotechnology Information) Genebank database under accession number arranging from KU216704 to KU216743, from KU291358 to KU291375 and from KU579260 to KU579276. The obtained sequences were aligned in NCBI Genebank database with the Basic Local Alignment Search Tool (http:// blast.ncbi.nlm.nih.gov) in order to confirm the maximum similarity sequences. Phylogenetic trees were constructed with MEGA 6.06 based on Neighbor-Joining (NJ) method ^33^.

### The investigation of NaF tolerance

All isolates from *E*. *superba* were evaluated their NaF-tolerance by adding NaF into the media at concentrations of 12%, 10%, 8%, 3%, 2%, 0.5% and 0% (control). The strains were incubated as following the fermentation procedures. All the incubations were monitored and compared with the control every 12 h. The fermentation broth was extracted three times with an equal volume of EtOAc and the whole EtOAc solutions were evaporated to dryness under reduced pressure and weighed (Tables 6, 7 and 8).

### Sample preparations for bioassay

The 75 microbial isolates were fermented under two different conditions with and without NaF. The bacteria were shaking at 4 °C, 180 rpm for 3 d in a 500 mL conical flask containing 150 mL LB liquid medium (Table 1). The actinobacterial were shaking at 4 °C, 180 rpm for 7 d in a 500 mL conical flask containing 150 mL liquid actinobacterial medium 2# (Table 1). The fungi were statically cultivated at 16 °C for 60 d in a 1000 mL conical flask containing 300 mL liquid fungal medium 2# (Table 1). The amounts of NaF added were 0.5% for fungi, 3% for bacteria, and 2%, 3%, 8%, and 10% for actinobacteria. Each experiment was conducted in three parallels. The fermentation broth was extracted three times with an equal volume of EtOAc and the whole EtOAc solutions were evaporated under reduced pressure to give the dried extracts for bioassay.

### Compounds isolation and identification

*Penicillium citrinum* OUCMDZ4136 was fermented under static conditions at 16 °C for 60 days in three hundred 1000 mL Erlenmeyer flasks each with 300 mL SWS liquid medium (Table 1) containing 0.5% NaF (Fig. S76). The harvested fermentation broths (27 L) were extracted thrice with equal volume of EtOAc that gave 10.3 g EtOAc extracts after removal of the solvents *in vacuum*. All extracts were separated by a silica-gel column eluting solution with petroleum ether–EtOAc (v/v1:0, 1:1) and then CH_2_Cl_2_-MeOH (v/v 1:0, 50:1, 30.1, 20:1, 10:1, 1:1 and 0:1) to get nine subfractions (Fr.1–Fr.9). The cytotoxic fraction 2 (218.9 mg) was subjected to isolation over a Sephadex LH-20 column eluting with MeOH to give twenty-five subfractions (Fr.2–1–Fr.2–25). Fr.2–12 (130.5 mg) was further separated by a Sephadex LH-20 column eluting with MeOH to give twenty subfractions (Fr.2–12–1–Fr.2–12–20). The active Fr.2-12-15 (89.3 mg) was further subjected to purification with HPLC ODS column (v/v MeOH−H_2_O 7:3) to yield compounds **3** (4.72 mg, t_R_ 10.5 min), **4** (9.96 mg, t_R_ 4.9 min), and **5** (5.93 mg, t_R_ 5.5 min). Fr.2–21 (35.6 mg) was further purified by semi-preparative HPLC over ODS column eluting with MeOH-H_2_O-CF_3_CO_2_H (v/v 70:30:0.15) to give compounds **1** (3.30 mg, t_R_ 5.1 min) and **2** (4.25 mg, t_R_ 6.9 min). The second cytotoxic fraction (Fr.8, 20.9 mg) was isolated by a Sephadex LH-20 column eluting with CH_2_Cl_2_-MeOH (v/v 1:1) to obtain thirty fractions. The main fraction (Fr.8-20, 13.4 mg) was further purified by HPLC ODS column (v/v MeCN-H_2_O 4:6) to yield compound **7** (8.39 mg, t_R_ 4.8 min). Fr.4 (89.7 mg) and Fr.5 (58.3 mg) were combined and separated over Sephadex LH-20 column eluting with CH_2_Cl_2_-MeOH (v/v 1:1) to give eight subfractions (Fr.4-1–Fr.4-8). The Fr.4-3 (33.7 mg) was further purified by semi-preparative HPLC over ODS column (v/v MeOH−H_2_O-CF_3_CO_2_H 60:40:0.15) to yield compound **6** (2.9 mg, t_R_ 13.1 min).

### 2,4-Dihydroxy-3,5,6-trimethylbenzoic acid (1)

yellow white powder; UV (MeOH) λ_max_ (logε) 257 (3.13), 264 (2.02), 213 (4.13) nm; IR (KBr) *ν*_max_ 3424, 2361, 1683, 1207, 1140 cm^−1^; ^1^H NMR (DMSO-*d*_*6*_, 600 MHz) *δ* 2.00 (s, 3H, H-8), 2.04 (s, 3H, H-9), 2.38 (s, 3H, H-10). ^13^C NMR (DMSO-*d*_*6*_, 150 MHz) *δ* 106.7 (qC, C-1), 159.1 (qC, C-2), 108.2 (qC, C-3), 157.2 (qC, C-4), 115.3 (qC, C-5), 136.5 (qC, C-6), 174.2 (qC, C-7), 9.0 (CH_3_, C-8), 12.3 (CH_3_, C-9), 18.3 (CH_3_, C-10); ESI-MS *m/z* 197.96 [M + H]^+^.

### Citreorosein (2)

yellow amorphous solid; UV (MeOH) *λ*_max_ (logε) 437 (2.63), 289 (4.12), 267 (4.02) nm; IR (KBr) *ν*_max_ 3083, 1682, 1571, 1398, 1212, 762 cm^−1^; ^1^H NMR (DMSO-*d*_*6*_, 600 MHz) *δ* 7.22 (s, 1H, H-2), 7.61 (s, 1H, H-4), 7.09 (d, *J* = 2.3 Hz, 1H, H-5), 6.57 (d, *J* = 2.3 Hz, 1H, H-7), 4.59 (s, 2H, H-11), 12.0 (s, 2H, 1/8-OH). ^13^C NMR (DMSO-*d*_*6*_, 150 MHz) *δ* 164.5 (qC, C-1), 120.8 (CH, C-2), 152.9 (qC, C-3), 117.1 (CH, C-4), 133.0 (qC, C-4a), 109.0 (CH, C-5), 135.2 (qC, C-5a), 161.5 (qC, C-6), 108.0 (CH, C-7), 165.8 (qC, C-8), 109.0 (qC, C-8a), 189.7 (qC, C-9), 181.4 (qC, C-10), 62.1 (CH_2_, C-11); HRESIMS *m/z* [M + H]^+^ 287.0545.

### Pinselin (3)

reddish brown amorphous powder; UV (MeOH) λ_max_ (logε) 384 (1.27), 290 (1.85), 263 (3.82) nm; IR (KBr) *ν*_max_ 2361, 1208, 1683 cm^−1^; ^1^H NMR (DMSO-*d*_*6*_, 600 MHz) *δ* 7.48 (d, *J* = 8.2 Hz, 1H, H-3), 7.61 (d, *J* = 8.2 Hz, 1H, H-4), 6.89 (s, 1H, H-5), 6.64 (s, 1H, H-7), 2.39 (s, 3H, H-10), 3.84 (s, 3H, H-11). ^13^C NMR (DMSO-*d*_*6*_, 150 MHz) *δ* 117.1 (qC, C-1), 117.2 (qC, C-1a), 150.8 (qC, C-2), 125.4 (CH, C-3), 120.1 (CH, C-4), 149.4 (qC, C-4a), 107.4 (CH, C-5), 155.5 (qC, C-5a), 149.4 (qC, C-6), 110.8 (CH, C-7), 160.4 (qC, C-8), 106.0 (qC, C-8a), 180.3 (qC, C-9), 22.1 (CH_3_, C-10), 167.3 (qC, C-11), 52.3 (CH_3_, C-12); HRESIMS *m/z* 301.0704 [M + H]^+^.

### Citrinin (4)

yellow needle crystal; UV (MeOH) λ_max_ (logε) 391 (1.31), 256 (2.56), 223 (4.45) nm; [α]_D_^25^−19.6 (*c* 0.35, MeOH); CD (*c* 0.02 M, MeOH) λ_max_ (Δε) 372 (−8.05), 311 (+13.92), 273 (+1.92), 265 (+2.54), 250 (−1.45) nm; IR (KBr) ν_max_ 3446, 2361, 1623,1610 cm^−1^; ^1^H NMR (DMSO-*d*_*6*_, 600 MHz) *δ* 8.61 (s, 1H, H-1), 4.98 (q, *J* = 6.7 Hz, 1H, H-3), 3.20 (q, *J* = 7.2 Hz, 1H, H-4), 1.26 (d, *J* = 6.7 Hz, 3H, H-10), 1.13 (d, *J* = 7.2 Hz, 3H, H-11), 1.96 (s, 3H, H-12). ^13^C NMR (DMSO-*d*_*6*_, 150 MHz) *δ* 166.9 (CH, C-1), 80.1 (CH, C-3), 33.5 (CH, C-4), 141.1 (qC, C-4a), 121.3 (qC, C-5), 182.6 (qC, C-6), 99.5 (qC, C-7), 176.5 (qC, C-8), 106.6 (qC, C-8a), 174.2 (qC, C-9), 17.6 (CH_3_, C-10), 18.0 (CH_3_, C-11), 9.1 (CH_3_, C-12); ESI-MS *m/z* 251.2 [M + H]^+^.

### Dihydrocitrinone (5)

yellow powder; UV (MeOH) λ_max_ (logε) 323 (2.75), 254 (2.42) nm; [α]_D_^25^ +42.7 (*c* 0.18 MeOH); CD (*c* 0.019 M, MeOH) λ_max_ (Δε) 338.5 (−2.86), 271 (+19.83), 266 (+19.27), 236 (+7.06); IR (KBr) *ν*_max_ 2977, 2361, 1653, 1596, 1402 cm^−1^; ^1^H NMR (DMSO-*d*_*6*_, 600 MHz) *δ* 4.61 (q, *J* = 6.6 Hz, 1H, H-3), 3.07 (q, *J* = 6.6 Hz, 1H, H-4), 1.19 (d, *J* = 6.6 Hz, 6H, H-10/11), 2.02 (s, 3H, H-12). ^13^C NMR (DMSO-*d*_*6*_, 150 MHz) *δ* 165.6 (qC, C-1), 78.2 (CH, C-3), 34.2 (CH, C-4), 146.9 (qC, C-4a), 112.9 (qC, C-5), 166.4 (qC, C-6), 101.4 (qC, C-7), 163.6 (qC, C-8), 99.0 (qC, C-8a), 173.4 (qC, C-9), 18.9 (CH_3_, C-10), 19.6 (CH_3_, C-11), 9.7 (CH_3_, C-12); ESI-MS *m/z* 267.2 [M + H]^+^.

### Pennicitrinone A (6)

orange yellow powder; UV (MeOH) λ_max_ (logε) 206 (0.558), 276 (0.759), 422 (0.726) nm; [α]_D_^25^ +66.5 (*c* 0.1 CHCl_3_); CD (*c* 0.013 M, MeOH) λ_max_ (Δε) 419 (+9.42), 324 (−5.93), 301 (+4.40), 244 (−0.39) nm; IR (KBr) ν_max_ 3470, 2340, 1204, 1663, 1652 cm^−1^; ^1^H NMR (DMSO-*d*_*6*_, 600 MHz) *δ* 5.35 (q, *J* = 6.5 Hz, 1H, H-3), 3.43 (q, *J* = 7.1 Hz, 1H, H-4), 6.76 (s, 1H, H-7), 1.33 (d, *J* = 6.5 Hz, 3H, H-9), 1.23 (d, *J*  =  7.1 Hz, 3H, H-10), 2.13 (s, 3H, H-11), 4.70 (dq, *J* = 3.5, 6.5 Hz,1H, H-3′), 3.34 (dq, *J* = 3.5, 6.9 Hz, 1H, H-4′), 1.33 (d, *J* = 6.5 Hz, 3H, H-9′), 1.27 (d, *J* = 6.9 Hz, 3H, H-10′), 2.23 (s, 3H, H-11′); ^13^C NMR (DMSO-*d*_*6*_, 150 MHz) *δ* 169.8 (qC, C-1), 84.2 (CH, C-3), 33.7 (CH, C-4), 136.4 (qC, C-4a), 125.9 (qC, C-5), 173.9 (qC, C-6), 100.1 (CH, C-7), 157.7 (qC, C-8), 101.5 (qC, C-8a), 18.4 (CH_3_, C-9), 18.9 (CH_3_, C-10), 10.3 (CH_3_, C-11), 87.9 (CH, C-3′), 44.3 (CH, C-4′), 143.6 (qC, C-4a′), 118.7 (qC, C-5′), 148.9 (qC, C-6′), 104.3 (qC, C-7′), 136.8 (qC, C-8′), 137.4 (qC, C-8a′), 20.8 (CH_3_, C-9′), 18.9 (CH_3_, C-10′), 12.1 (CH_3_, C-11′); ESI-MS *m/z* 379.17 [M - H]^−^.

### Quinolactacin A1 (7)

white solid. UV (MeOH) λ_max_ (logε) 315(3.09), 371(1.18), 236(3.52) nm; IR (KBr) ν_max_ 2340, 1204, 1663, 1652; [α]_D_^25^ +28.5(*c* 0.1 CHCl_3_), ^1^H NMR (DMSO-*d*_6_, 600 MHz) *δ* 4.94 (s, 1H, H-3), 3.80 (s, 3H, N-CH_3_), 8.00 (d, *J* = 8.2 Hz, 1H, H-5), 7.89 (dd, *J* = 8.2, 7.6 Hz, 1H, H-6), 7.58 (dd, *J*  = 7.6, 7.7 Hz, 1H, H-7), 8.23 (d, *J*  = 7.7 Hz, 1H, H-8), 2.20 (m, 1H, H-1′), 0.43 (d, *J*  = 6.5 Hz, 1H, 1’-CH_3_), 1.39 (m, 1H, H-2′), 1.59 (m, 1H, H-2′), 1.00 (t, *J*  = 7.7 Hz, 3H, H-3′). ^13^C NMR (DMSO-*d*_6_, 150 MHz) δ 169.0 (qC, C-1), 57.9 (CH, C-3), 163.9 (qC, C-3a), 35.8 (CH_3_, *N*-CH_3_), 141.5 (qC, C-4a), 118.4 (CH, C-5), 133.6 (CH, C-6), 126.0 (CH, C-7), 126.1 (CH, C-8), 130.2 (qC, C-8a), 170.8 (qC, C-9), 107.8 (qC, C-9a), 35.8 (CH, C-1′), 11.7 (CH_3_, 1′-CH_3_), 27.4 (CH_2_, C-2′), 12.0 (CH_3_, C-3′).

### Evaluation for antibacterial activity

A paper disk-agar diffusion method^34^ was applied into the antibacterial assay against six aquatic pathogenic bacteria (*E*. *tarda, V*. *parahemolyticus, V*. *vulnificus, V*. *harveyi, V*. *alginolyticus*, and *B. cereus*) and five human pathogenic bacteria (*S*. *aureus, E*. *coli, E*. *aerogenes, B*. *subtilis*, and *P*. *aeruginosa*). The initial concentration of sample was 1.0 mg/mL for EtOAc extracts and 0.1 mg/mL for compounds. Ciprofloxacin with the concentration of 0.1 mg/mL was used as a positive control. Pathogenic bacteria were inoculated into a 50 mL tube containing 20 mL liquid medium and shaken at 28 °C and 180 rpm. Aquatic pathogenic bacteria were cultured in 2216E medium for 24 h, human pathogenic bacteria were cultured in LB liquid media for 12 h. The bacteria suspension was coated on the corresponding agar plate, and cultured at room temperature for 30 min. Paper discs that absorbed 10 μL of sample and positive control solution were volatilized to dry and placed onto the agar plate coated with the bacterial suspension. Every sample was tested based on three parallels. The bacterial static plates were incubated for 12 h at 28 °C. The inhibition zone was observed every hour for 16 h and the inhibition zone diameters were recorded (Table 4 and Fig. 6). Once the antibacterial activity was observed, the MIC value was measured by conventional serious two-fold agar dilution methods. The MIC was defined as the lowest corresponding concentration adsorbed in paper around which no bacterial growth was observed. The solution of 1.0 mg/mL was further diluted to 500, 250, 125, and 62.5 μg/mL (Fig. 7A–G). If the inhibition zone could still be observed, the solution of 62.5 μg/mL was further diluted to 31.25, 15.62, 7.81, and 3.90 μg/mL (Fig. 7H).

### Cytotoxic Assay

Cytotoxicities for MCF-7 and A549, and for K562 cell lines (Table 5) were evaluated by MTT^35^ and CCK-8^36^ methods, respectively. The EtOAc extracts, compounds and adriamycin (the positive control) were dissolved in DMSO with the concentration of 2 mg/mL, 2 mM, and 0.2 mM, respectively. Then, 10 μL of the DMSO solution was diluted into 990 μL RPMI-1640 medium and the testing solution was formulated as 200 μg/mL for EtOAc extracts, 20 μM for compounds and 2 μM for adriamycin. The cell lines were grown in RPMI-1640 medium with 10% FBS under a humidified atmosphere of 5% CO_2_ and 95% air at 37 °C. The cell suspension, 100 μL, with 5 × 10^3^ cells/well for MCF-7 and A549 and 1 × 10^4^ cells/well for K562 were inoculated into the 96-well plates and cultured under the above conditions for 24 h. The contents of each well was added to 100 μ0 of the testing solutions in triplicate and incubated for 72 h; the final concentration for each extract, compound and adriamycin was 100 μg/mL, 10 μM, and 1 μM, respectively. The RPMI-1640 medium containing 5% FBS was used as the blank control. The MTT solution with the concentration of 5 mg/mL in FBS, 20 μL, was added into each well and incubated for 4 h at 37 °C. The medium containing MTT was gently pipetted and each well was added 150 μL DMSO to dissolve the formed formazan crystals. Absorbance was recorded on a Spectra Max Plus plate reader at 570 nm. As for K562 cell line, each well was added 10 μL CCK-8 solution (5 mg/mL) to dye and incubated for 6 h. The solution was directly used to measure the absorbance at 450 nm. The cytotoxicity was represented as the inhibition that was calculated as (OD_blank_ − OD_sample_)/OD_blank_ × 100%. If the inhibition is more than 50%, the IC_50_ value that is defined as the concentration of the compound to induce 50% inhibition, was measured by continuous dilution into 5, 2.5, 1.25, 0.625, and 0.312 μM, and then calculated by SPSS (Statistical Product and Service Solutions) v19.0 software.

## Additional Information

**How to cite this article**: Cui, X. *et al*. Diversity and function of the Antarctic krill microorganisms from *Euphausia superba*. *Sci. Rep*. **6**, 36496; doi: 10.1038/srep36496 (2016).

**Publisher’s note:** Springer Nature remains neutral with regard to jurisdictional claims in published maps and institutional affiliations.

## Supplementary Material

Supplementary Information

## Figures and Tables

**Table 1:** Ingredient of media used for isolation and fermentation of microbial strains from *E*. *superba*^a^.

Microbes	Medium	Ingredient (L^−1^)
Actinobacteria	GYM	4.0 g dextrose,4.0 g yeast extract,10.0 g malt extract,2.0 g CaCO_3_,12.0 g agar
	ISP2	4.0 g yeast extract,10.0 g malt extract,4.0 g dextrose,20.0 g agar
	Chitin	2.0 g chitin, 1.0 g NH_4_Cl, 0.9 g K_2_HPO_4_·3H_2_O, 0. g KH_2_PO_4_, 0.5 g MgSO_4_·7H_2_O 0.001 g FeSO_4_, 0.001 g ZnSO_4_, 0.1 g CaF_2_, 20.0 g agar
	M5	20.0 g agar
	SC	10.0 g soluble starch, 0.3 g casein, 2.0 g KNO_3_, 2.0 g NaCl, 2.0 g K_2_HPO_4_, 0.05 g MgSO_4_·7H_2_O, CaCO_3_ 0.02 g, 0.01 g FeSO_4_·7H_2_O, 20.0 g agar
	Actinobacterial medium 2#^b^	20.0 g glucose, 10.0 g soluble starch, 10.0 g peptone, 10 g yeast extract, 3.0 g beef extract, 0.5 g KH_2_PO_4_, 0.5 g MgSO_4_, 2.0 g CaCO_3_, pH 7.5–8.0.
Fungi	PDA	200 g potato extract, 20 g dextrose, 20.0 g agar
	Emerson	1.0 g dextrose,1.0 g yeast extract powder,4.0 g peptone,2.5 g NaCl,18.0 g agar
	Sabouraud	10.0 g peptone, 40.0 g maltose, 20.0 g agar
	Fungal medium 2#^b^	20.0 g maltose,10.0 g aginomoto,0.5 g KH_2_PO_4_, 0.3 g MgSO_4_·7H_2_O,10.0 g dextrose, 3.0 gyeast extract, 1.0 g corn steep liquor,20.0 g mannitol, pH6.5
	SWS^b^	1.0 g peptone, 10.0 g soluble starch, pH 7.5–8.0
Bacteria	Marine agar 2216^c^	5.0 g peptone, 1.0 g yeast extract, 0.1 g ferric citrate, 0.08 g KBr, 19.45 g NaCl, 8.8 g MgCl_2_, 3.24 g Na_2_SO_4_, 1.8 g CaCl_2_, 0.55 g KCl, 0.16 g NaHCO_3_, 0.0016 g, NH_4_NO_3_, 0.008 g Na_2_HPO_4_, 0.022 g H_3_BO_3_, 20.0 g agar
	Luria Bertani (LB)^b^,c	5.0 g yeast extract, 10.0 g peptone, 5.0 g NaCl, 15.0 g agar
	Zobell 2216E	5.0 g peptone,3.0 g yeast extract,15.0–20.0 g agar
	Glucose Yeast Extract Starch	10.0 g dextrose, 10.0 g yeast extract,10.0 g starch, 5.0 g NaCl, 3.0 g CaCO_3_, 20.0 gagar, pH 7.0
	Penssay Broth	1.5 g beef extract, 1.5 g yeast extract, 5.0 g tryptone, 3.5 g NaCl, 1.0 g dextrose, 4.8 g K_2_HPO_4_·3H_2_O, pH 7.2

^a^All media were prepared with natural sterile seawater and adjusted corresponding pH prior to autoclaving at 121 °C for 20 min.

^b^The liquid media were used to ferment actinobacteria, bacteria and fungi in bioassay and compound isolation, respectively.

^c^The 2216E and LB media were also used to culture aquatic and human pathogenic bacteria in the antibacterial tests, respectively.

**Table 2:** Phylogenetic affiliations of culturable isolates from *E*. *superba*.

Phylum	Class	Order	Family	Genus	Strain No.	Genebank accession No.	Top Blast match No.	Identity
*Ascomycota*	*Eurotiomycetes*	*Eurotiales*	*Trichocomaceae*	*Penicillium*	OUCMDZ4115	KU216704	AM745115.1	99%
					OUCMDZ4116	KU216705	NR111815.1	99%
					OUCMDZ4117	KU216706	NR121224.1	100%
					OUCMDZ4118	KU216707	AF033422.1	99%
					OUCMDZ4121	KU216709	AF033422.1	99%
					OUCMDZ4122	KU216710	KP942910.1	99%
					OUCMDZ4124	KU216712	NR121224.1	98%
					OUCMDZ4126	KU216714	AF033422.1	99%
					OUCMDZ4127	KU216715	AF033422.1	99%
					OUCMDZ4129	KU216716	KU613360.1	99%
					OUCMDZ4130	KU216717	KR706304.1	99%
					OUCMDZ4132	KU216719	KM250379.1	100%
					OUCMDZ4133	KU216720	AF033422.1	98%
					OUCMDZ4134	KU216721	NR121224.1	100%
					OUCMDZ4136	KU216722	AF033422.1	99%
					OUCMDZ4137	KU216723	KF973341.1	100%
					OUCMDZ4139	KU216724	NR111673.1	99%
					OUCMDZ4140	KU216725	NR111815.1	99%
					OUCMDZ4141	KU216726	NR111673.1	98%
					OUCMDZ4143	KU216727	NR121224.1	100%
					OUCMDZ4145	KU216728	NR121224.1	100%
					OUCMDZ4149	KU216729	KF973341.1	99%
					OUCMDZ4150	KU216730	NR121224.1	99%
					OUCMDZ4151	KU216731	NR077145.1	99%
					OUCMDZ4152	KU216732	AF033422.1	99%
					OUCMDZ4154	KU216733	AY373902.1	97%
					OUCMDZ4156	KU216735	AF033422.1	99%
					OUCMDZ4161	KU216736	AF033422.1	100%
					OUCMDZ4162	KU579276	HQ026745.1	99%
					OUCMDZ4163	KU216737	AY373902.1	99%
					OUCMDZ4164	KU216738	KJ173539.1	99%
					OUCMDZ4166	KU216739	AY373902.1	99%
					OUCMDZ4167	KU216740	NR111815.1	99%
					OUCMDZ4168	KU216741	NR121224.1	99%
					OUCMDZ4169	KU216742	NR111815.1	98%
					OUCMDZ4170	KU216743	NR077145.1	100%
				*Talaromyces*	OUCMDZ4125	KU216713	KF984795.1	100%
				*Aspergillus*	OUCMDZ4120	KU216708	NR111041.1	99%
					OUCMDZ4131	KU216718	EU821315.1	98%
	*Dothideomycetes*	*Capnodiales*	*Mycosphaerellaceae*	*Cladosporium*	OUCMDZ4138	KU579275	GU595035.1	99%
					OUCMDZ4155	KU216734	KJ596569.1	99%
	*Saccharomycetes*	*Saccharomycetales*	*Debaryomycetaceae*	*Meyerozyma*	OUCMDZ4123	KU216711	KF619545.1	98%
*Firmicutes*	*Bacilli*	*Bacillales*	*Bacillaceae*	*Bacillus*	OUCMDZ4186	KU291364	NR117946.1	99%
					OUCMDZ4181	KU579260	NR112636.1	99%
					OUCMDZ4190	KU291366	KF811045.1	99%
					OUCMDZ4191	KU291367	KJ149809.1	100%
					OUCMDZ4192	KU291368	FN597644.1	99%
					OUCMDZ4205	KU579266	NR116022.1	100%
					OUCMDZ4206	KU291370	KF954553.1	99%
					OUCMDZ4207	KU579267	KR045745.1	99%
					OUCMDZ4208	KU291371	NR113945.1	99%
					OUCMDZ4210	KU291372	NR041794.1	100%
					OUCMDZ4211	KU579268	KT719214.1	100%
					OUCMDZ4213	KU291373	KJ149809.1	99%
					OUCMDZ4215	KU579270	NR041455.1	99%
			*Staphylococcaceae*	*Staphylococcus*	OUCMDZ4185	KU579262	NR113349.1	99%
			*Planococcaceae*	*Planococcus*	OUCMDZ4188	KU291365	NR113814.1	99%
*Proteobacteria*	*Gammaproteobacteria*	*Pseudomonadales*	*Moraxellaceae*	*Psychrobacter*	OUCMDZ4187	KU579263	KJ475192.1	99%
					OUCMDZ4189	KU579264	NR041455.1	99%
					OUCMDZ4199	KU291369	NR027225.1	99%
					OUCMDZ4202	KU579265	HM584295.1	99%
					OUCMDZ4212	KU579269	NR027225.1	100%
					OUCMDZ4214	KU291374	NR028918.1	99%
					OUCMDZ4218	KU579271	NR043057.1	98%
					OUCMDZ4219	KU579272	NR028948.1	99%
					OUCMDZ4221	KU579274	NR028948.1	99%
	*Betaproteobacteria*	*Burkholderiales*	*Alcaligenaceae*	*Advenella*	OUCMDZ4222	KU291375	KF844056.1	100%
*Actinobacteria*	*Actinobacteria*	*Streptomycineae*	*Streptomycetaceae*	*Streptomyces*	OUCMDZ4173	KU291358	KU317912.1	99%
					OUCMDZ4174	KU291359	KM513543.1	99%
					OUCMDZ4176	KU291360	KP718508.1	99%
					OUCMDZ4177	KU291361	NR115669.1	99%
					OUCMDZ4179	KU291362	EU790486.1	99%
					OUCMDZ4182	KU579261	NR 041146.1	99%
					OUCMDZ4183	KU291363	NR041173.1	99%
					OUCMDZ4220	KU579273	NR041173.1	100%

**Table 3:** The classification of culturable isolates from *E*. *superba*.

	*Phylum*	Class	Order	Family	Genus	Quantity
	*Ascomycota*	*Eurotiomycetes*	*Eurotiales*	*Trichocomaceae*	*Penicillium*	36
					*Talaromyces*	1
					*Aspergillus*	2
		*Dothideomycetes*	*Capnodiales*	*Mycosphaerellaceae*	*Cladosporium*	2
		*Saccharomycetes*	*Saccharomycetales*	*Debaryomycetaceae*	*Meyerozyma*	1
	*Firmicutes*	*Bacilli*	*Bacillales*	*Bacillaceae*	*Bacillus*	13
					*Staphylococcus*	1
					*Planococcus*	1
	*Proteobacteria*	*Gammaproteobacteria*	*Pseudomonadales*	*Moraxellaceae*	*Psychrobacter*	9
		*Betaproteobacteria*	*Burkholderiales*	*Alcaligenaceae*	*Advenella*	1
	*Actinobacteria*	*Actinobacteria*	*Streptomycineae*	*Streptomycetaceae*	*Streptomyces*	8
Total	4	7	7	7	11	75

**Table 4:** The antibacterial activity of the EtOAc extracts at 1000 μg/mL^a^^,^ b.

Order	Family	Genus	Strain No.^c^	*E*. *tarda*	*V. vulnificus*	*V. harveyi*	*V*. *alginolyticus*	*B. cereus*	*S*. *aureus*	*E*. *coli*	*B. subtilis*	*P. aeruginosa*
*Eurotiales*	*Trichocomaceae*	*Penicillium*	OUCMDZ4129	+	−	−	−	−	−	−		
			OUCMDZ4134	+	−	−	−	−	−	−		
			OUCMDZ4139	−	+	−	−	−	−	−		
			OUCMDZ4140	+	+	−	−	−	−	−		
			OUCMDZ4141	+	+	−	−	−	−	−		
			OUCMDZ4162	−	++	−	-	−	−	−		
			OUCMDZ4163	−	−	−	−	+	−	−		
			OUCMDZ4164	−	−	−	−	+	−	−		
*Capnodiales*	*Mycosphaerellaceae*	*Cladosporium*	OUCMDZ4138	−	+	−	−	−	−	−		
*Streptomycineae*	*Streptomycetaceae*	*Streptomyces*	OUCMDZ4173	++	−	+	+	+	−	−		
			OUCMDZ4174	+	−	+	+	+	−	-		
			OUCMDZ4176	+	−	+	−	−	−	−		
			OUCMDZ4177	+	−	+	−	−	−	−		
			OUCMDZ4179	+	−	−	−	−	−	−		
*Bacillales*	*Bacillaceae*	*Bacillus*	OUCMDZ4186	+	+++	+	+	+	+	−		
			OUCMDZ4181	−	−	−	−	−	−	−		
			OUCMDZ4190	−	+	+	−	+	−	−		
			OUCMDZ4191	−	+++	+	+++	+	+	+		+
			OUCMDZ4192	−	+	−	−	−	+	+		+
			OUCMDZ4205	−	−	−	−	−	+	−		
			OUCMDZ4206	+	−	−	−	−	+	−		
			OUCMDZ4207	++	+	+	+	−	−	−		
			OUCMDZ4208	+	+++	+	+	−	−	−		
			OUCMDZ4211	−	−	−	−	−	−	−	+	
			OUCMDZ4213	+	+	+	+	+	−	−		
			OUCMDZ4215	−	+	−	−	−	+	−		
	*Planococcaceae*	*Planococcus*	OUCMDZ4188	++	+++	+	+	+	+	−		
*Pseudomonadales*	*Moraxellaceae*	*Psychrobacter*	OUCMDZ4187	+	+++	−	−	−	−	−		
			OUCMDZ4189	−	+++	+	−	+	−	−		
			OUCMDZ4214	−	+	−	+++	+	+	−		
			OUCMDZ4218	−	−	+	−	+	−	−		
			OUCMDZ4219	−	−	+++	-	−	−	−		
*Burkholderiales*	*Alcaligenaceae*	*Advenella*	OUCMDZ4222	+	−	+	+	+	−	−		

^a^+++ strong: inhibition zone diameter (IZD) ≥18 mm; ++ moderate: IZD = 10–17 mm; +weak: IZD< 10 mm; - non active: IZD = 0 mm. All metabolites didn’t show any activity against *Vibrio parahemolyticus* and *Enterobacter aerogenes*.

^b^IDZs of ciprofloxacin (positive control) at 100 μg/mL were 20, 25, 20, 18, 25, 25 and 15 mm for *Edwardsiella tarda*, *Vibrio vulnificus*, *V. alginolyticus*, *V. harveyi*, *Bacillus cereus*, *Escherichia coli*, and *Staphylococcus aureus*, respectively.

^c^Fungi, bacteria, and actinobacteria were cultured in the fungal medium 2#, LB medium, and actinobacterial medium 2#, respectively.

**Table 5:** Cytotoxicity of the EtOAc extracts of microbial metabolites at 100 μg/mL^a^^,^ b.

Strain No.^c^	K562 (%)	MCF-7 (%)	A549 (%)	Strain No.	K562 (%)	MCF-7 (%)	A549 (%)
OUCMDZ4115	67.85 ± 1.45	63.71 ± 1.46	-	OUCMDZ4164	69.28 ± 2.03	65.27 ± 5.89	--
OUCMDZ4116	70.75 ± 1.06	77.29 ± 2.73	66.19 ± 2.43	OUCMDZ4166	70.75 ± 0.25	60.16 ± 1.74	-
OUCMDZ4117	69.16 ± 0.47	75.27 ± 3.09	79.57 ± 1.50	OUCMDZ4167	-	55.39 ± 0.93	--
OUCMDZ4118	67.69 ± 0.51	65.04 ± 3.92	--	OUCMDZ4168	69.18 ± 0.61	58.26 ± 1.73	--
OUCMDZ4120	70.32 ± 0.09	80.64 ± 1.56	82.56 ± 1.72	OUCMDZ4169	68.54 ± 0.84	73.27 ± 1.59	--
OUCMDZ4121	69.58 ± 1.74	81.94 ± 0.34	76.05 ± 5.29	OUCMDZ4170	-	-	--
OUCMDZ4122	−60.04 ± 4.89	−203.32 ± 16.80	−139.52 ± 15.91	OUCMDZ4173	-	-	--
OUCMDZ4123	--	--	-	OUCMDZ4174	--	--	--
OUCMDZ4124	64.49 ± 0.73	58.08 ± 1.36	-	OUCMDZ4176	65.18 ± 7.17	--	−38.10 ± 3.45
OUCMDZ4125	68.88 ± 1.77	81.83 ± 1.33	81.00 ± 0.54	OUCMDZ4177	69.79 ± 1.64	--	-
OUCMDZ4126	65.88 ± 0.42	61.20 ± 1.34	-	OUCMDZ4179	61.21 ± 0.43	−22.39 ± 14.02	−25.41 ± 6.49
OUCMDZ4127	71.94 ± 0.57	57.85 ± 0.18	-	OUCMDZ4185	59.96 ± 9.93	-	-
OUCMDZ4129	64.46 ± 1.18	--	--	OUCMDZ4186	-	81.46 ± 1.14	-
OUCMDZ4130	72.64 ± 1.08	75.96 ± 1.20	78.23 ± 2.68	OUCMDZ4187	64.12 ± 1.81	-	−18.24 ± 0.19
OUCMDZ4131	55.57 ± 1.66	--	--	OUCMDZ4188	62.65 ± 3.47	84.41 ± 6.36	-
OUCMDZ4132	71.26 ± 0.13	76.35 ± 0.92	59.32 ± 3.33	OUCMDZ4189	82.35 ± 0.82	77.15 ± 2.04	--
OUCMDZ4133	64.73 ± 1.89	62.64 ± 1.74	--	OUCMDZ4190	56.94 ± 4.01	78.31 ± 1.23	--
OUCMDZ4134	62.85 ± 0.40	59.78 ± 1.25	-	OUCMDZ4191	54.30 ± 2.60	68.02 ± 4.83	62.75 ± 6.12
OUCMDZ4136	67.50 ± 0.60	68.62 ± 5.84	-	OUCMDZ4192	52.81 ± 3.03	76.56 ± 2.85	57.01 ± 4.71
OUCMDZ4137	63.87 ± 1.85	-	--	OUCMDZ4199	-	--	54.17 ± 11.32
OUCMDZ4138	73.24 ± 1.91	62.45 ± 7.03	--	OUCMDZ4202	57.28 ± 9.45	--	--
OUCMDZ4139	64.10 ± 1.11	--	--	OUCMDZ4205	-	-	-
OUCMDZ4140	60.86 ± 3.46	53.67 ± 5.15	--	OUCMDZ4206	68.36 ± 1.26	--	61.81 ± 1.92
OUCMDZ4141	57.71 ± 8.11	71.73 ± 2.31	--	OUCMDZ4207	56.76 ± 4.74	54.91 ± 2.54	--
OUCMDZ4143	69.14 ± 0.79	61.23 ± 2.19	--	OUCMDZ4208	--	53.05 ± 0.78	--
OUCMDZ4145	68.94 ± 1.09	81.36 ± 0.31	-	OUCMDZ4210	--	52.98 ± 1.05	--
OUCMDZ4149	-	--	--	OUCMDZ4211	65.67 ± 1.86	58.85 ± 7.02	53.82 ± 0.98
OUCMDZ4150	63.98 ± 3.15	--	--	OUCMDZ4212	--	--	--
OUCMDZ4151	-	--	--	OUCMDZ4213	77.65 ± 2.96	65.85 ± 0.80	54.08 ± 8.42
OUCMDZ4152	-	--	-	OUCMDZ4218	--	58.26 ± 6.04	--
OUCMDZ4154	71.98 ± 0.35	52.09 ± 1.83	75.97 ± 0.35	OUCMDZ4214	-	--	--
OUCMDZ4155	-	--	-	OUCMDZ4215	-	-	-
OUCMDZ4156	65.99 ± 2.90	57.00 ± 2.69	-	OUCMDZ4219	--	-	--
OUCMDZ4161	66.93 ± 4.16	60.98 ± 0.35	-	OUCMDZ4220	--	−54.84 ± 9.91	−116.64 ± 8.18
OUCMDZ4162	61.86 ± 0.12	62.05 ± 6.69	-	OUCMDZ4221	--	--	--
OUCMDZ4163	72.19 ± 0.36	73.25 ± 5.32	63.43 ± 7.16	OUCMDZ4222	-	-	-
OUCMDZ4183	59.23 ± 1.28	--	-	OUCMDZ4181	--	--	--
OUCMDZ4182	-	-	-				

^a^Each datum represents the average inhibition and the standard deviation of three independent experiment: <50% inhibition (-, weak), <30% inhibition (--, very weak).

^b^The inhibitions of adriamycin (positive control) at 1 μM for K562, MCF-7 and A549 were 72%, 65% and 75%, respectively.

^c^Fungal strains OUCMDZ4115–4170 were cultured in the fungal medium 2#; Bacteria strains OUCMDZ4185–4219, 4181, 4221 and 4222 were cultured in the LB medium; Actinobacterial strains OUCMDZ4173–4179, 4182, 4183 and 4220 were cultured in the actinobacterial medium 2#.

**Table 6:** The production of the fungal metabolites in the fungal medium 2# (mg/mL).

Strain No.	Without NaF	0.5% NaF	Strain No.	Without NaF	0.5% NaF
OUCMDZ4115	0.323	0.080	OUCMDZ4139	0.487	0.013
OUCMDZ4116	0.553	0.012	OUCMDZ4140	0.082	0.079
OUCMDZ4117	0.573	0.167	OUCMDZ4141	0.451	0.189
OUCMDZ4118	0.485	0.102	OUCMDZ4143	0.550	0.208
OUCMDZ4120	0.718	0.269	OUCMDZ4145	0.348	0.054
OUCMDZ4121	0.583	0.173	OUCMDZ4149	0.253	0.013
OUCMDZ4122	0.177	0.071	OUCMDZ4150	0.413	0.044
OUCMDZ4123	0.368	0.128	OUCMDZ4151	0.248	0.076
OUCMDZ4124	0.591	0.244	OUCMDZ4152	0.315	0.184
OUCMDZ4125	0.425	0.156	OUCMDZ4154	0.259	0.192
OUCMDZ4126	0.662	0.256	OUCMDZ4155	0.376	0.155
OUCMDZ4127	0.439	0.107	OUCMDZ4156	0.547	0.108
OUCMDZ4129	0.779	0.138	OUCMDZ4161	0.267	0.034
OUCMDZ4130	0.055	0.037	OUCMDZ4162	0.533	0.196
OUCMDZ4131	0.901	0.329	OUCMDZ4163	0.519	0.150
OUCMDZ4132	0.645	0.281	OUCMDZ4164	0.692	0.244
OUCMDZ4133	0.636	0.213	OUCMDZ4166	0.511	0.034
OUCMDZ4134	0.647	0.335	OUCMDZ4167	0.413	0.214
OUCMDZ4136	0.515	0.128	OUCMDZ4168	0.393	0.019
OUCMDZ4137	0.038	0.029	OUCMDZ4169	0.687	0.021
OUCMDZ4138	0.507	0.144	OUCMDZ4170	0.265	0.099

**Table 7:** The production of the bacterial metabolites in the LB medium (mg/mL).

Strain No.	Without NaF	3% NaF	Strain No.	Without NaF	3% NaF
OUCMDZ4186	0.299	0.015	OUCMDZ4208	0.349	0.025
OUCMDZ4187	0.185	0.030	OUCMDZ4210	0.283	0.013
OUCMDZ4188	0.042	0.002	OUCMDZ4211	0.250	0.002
OUCMDZ4189	0.382	0.019	OUCMDZ4212	0.201	0.013
OUCMDZ4190	0.095	0.005	OUCMDZ4213	0.143	0.006
OUCMDZ4191	0.067	0.002	OUCMDZ4218	0.067	0.008
OUCMDZ4192	0.331	0.015	OUCMDZ4214	0.233	0.016
OUCMDZ4199	0.025	0.003	OUCMDZ4215	0.185	0.006
OUCMDZ4202	0.080	0.001	OUCMDZ4219	0.621	0.005
OUCMDZ4205	0.257	0.004	OUCMDZ4185	0.260	0.025
OUCMDZ4206	0.215	0.002	OUCMDZ4221	0.164	0.003
OUCMDZ4207	0.113	0.006	OUCMDZ4222	0.215	0.004
OUCMDZ4181	0.173	0.008			

**Table 8:** The production of the actinobacterial metabolites in the actinobacterial medium 2# (mg/mL).

Strain No.	Without NaF	2% NaF	3% NaF	8% NaF	10% NaF
OUCMDZ4173	0.698	0.188	0.089	0.047	0.026
OUCMDZ4174	0.289	0.077	0.043	0.034	0.017
OUCMDZ4176	0.427	0.038	0.068	0.015	0.032
OUCMDZ4177	0.350	0.058	0.019	0.009	0.009
OUCMDZ4179	0.129	0.029	0.024	0.019	0.016
OUCMDZ4182	0.206	0.024	0.016	0.013	0.011
OUCMDZ4183	0.192	0.019	0.015	0.003	0.001
OUCMDZ4220	0.287	0.082	0.053	0.019	0.009

**Table 9:** Cytotoxicity of the EtOAc extracts of actinobacterial metabolites under NaF^a^^,^ b.

Strain No.^c^	K562 (%)	2% NaF			3% NaF			8% NaF			10% NaF	
		MCF-7(%)	A549 (%)	K562 (%)	MCF-7(%)	A549 (%)	K562 (%)	MCF-7(%)	A549 (%)	K562 (%)	MCF-7(%)	A549 (%)
OUCMDZ4173	32.88 ± 0.04	63.96 ± 0.17	−4.55 ± 0.37	48.50 ± 0.06	69.97 ± 0.28	−6.81 ± 0.02	−4.53 ± 0.19	45.53 ± 0.11	7.89 ± 0.09	2.47 ± 0.07	67.20 ± 0.09	18.25 ± 0.01
OUCMDZ4174	6.99 ± 0.12	58.46 ± 0.07	29.64 ± 0.05	58.54 ± 0.23	54.43 ± 0.03	−6.63 ± 0.24	11.36 ± 0.45	27.62 ± 0.18	43.90 ± 0.08	−3.55 ± 0.83	63.23 ± 0.29	17.39 ± 0.09
OUCMDZ4176	9.73 ± 0.07	76.67 ± 0.38	29.73 ± 0.36	−2.51 ± 0.59	71.91 ± 0.54	13.80 ± 0.04	36.81 ± 0.36	66.83 ± 0.65	36.64 ± 0.13	4.08 ± 0.58	72.68 ± 0.04	28.20 ± 0.06
OUCMDZ4177	8.43 ± 0.03	56.66 ± 0.01	13.86 ± 0.08	43.68 ± 0.56	46.93 ± 0.37	31.90 ± 0.12	1.82 ± 0.56	51.40 ± 0.05	10.15 ± 0.06	5.02 ± 0.54	51.79 ± 0.11	35.47 ± 0.15
OUCMDZ4179	21.67 ± 0.16	32.40 ± 0.92	42.81 ± 0.81	26.88 ± 0.04	74.63 ± 0.08	−282.72 ± 0.73	29.29 ± 0.62	63.17 ± 0.06	22.74 ± 0.06	1.62 ± 0.53	75.98 ± 0.012	13.51 ± 0.01

^a^Each datum represents the average inhibition and the standard deviation of three independent experiment.

^b^The inhibitions of adriamycin (positive control) at 1 μM for K562, MCF-7 and A549 were 73%, 71% and 74%, respectively.

^c^Cultured in the actinobacterial medium 2#.

**Table 10:** Cytotoxicity of the EtOAc extracts of fungal metabolites under 0.5% NaFa,^b^.

Strain No.	K562 (%)	Fungal medium 2#			SWS medium	
MCF-7 (%)	A549 (%)	K562 (%)	MCF-7 (%)	A549 (%)		
OUCMDZ4118	79.32 ± 0.18	86.23 ± 0.25	84.15 ± 0.01	79.71 ± 0.61	60.19 ± 0.01	61.41 ± 0.01
OUCMDZ4122	62.90 ± 0.33	79.23 ± 0.37	79.16 ± 0.07	85.06 ± 0.82	63.99 ± 0.14	61.53 ± 0.03
OUCMDZ4130	57.19 ± 0.65	84.49 ± 0.004	85.09 ± 0.72	85.65 ± 1.12	64.06 ± 0.02	57.35 ± 0.17
OUCMDZ4131	17.36 ± 0.07	76.61 ± 0.01	63.97 ± 0.002	3.89 ± 0.03	49.85 ± 0.02	1.91 ± 0.49
OUCMDZ4134	63.20 ± 0.52	87.40 ± 0.07	75.72 ± 0.05	89.37 ± 0.02	72.25 ± 0.21	77.72 ± 0.02
OUCMDZ4136	40.90 ± 0.34	75.35 ± 0.06	65.77 ± 0.01	87.70 ± 0.19	80.00 ± 0.02	66.61 ± 0.07
OUCMDZ4137	80.86 ± 0.01	61.68 ± 0.22	73.00 ± 0.25	26.73 ± 1.07	66.88 ± 0.24	50.59 ± 0.01
OUCMDZ4145	50.36 ± 0.66	88.07 ± 0.45	87.74 ± 0.01	12.59 ± 4.10	55.43 ± 0.04	64.87 ± 0.21

^a^Each datum represents the average inhibition and the standard deviation of three independent experiment.

^b^The inhibitions of adriamycin (positive control) at 1 μM for K562, MCF-7 and A549 were 87%, 60% and 83%, respectively.

**Table 11:** Cytotoxicity of seven compounds from *P*. *citrinum* OUCMDZ4136 under 0.5% NaF^a^.

Compounds	MCF-7 (IC_50_/μM)	A549 (IC_50_/μM)	K562 (IC_50_/μM)
**1**	4.29 ± 0.12	7.10 ± 0.04	10.75 ± 0.03
**2**	3.60 ± 0.03	2.20 ± 0.06	5.43 ± 0.022
**3**	6.10 ± 0.13	5.60 ± 0.02	7.80 ± 0.12
**4**	4.20 ± 0.06	13.90 ± 0.05	13.50 ± 0.03
**5**	6.50 ± 0.02	>100	15.37 ± 0.06
**6**	13.15 ± 0.05	19.80 ± 0.09	12.60 ± 0.03
**7**	3.92 ± 0.02	3.00 ± 0.17	8.96 ± 0.16
Adriamycin	1.00 ± 0.14	1.38 ± 0.11	0.34 ± 0.01

^a^Each datum represents the average and the standard deviation of three independent experiment.
