# Supplementary material for: Diversity and function of the Antarctic krill microorganisms from Euphausia superba
Source: Sci Rep. 2016 Nov 4;6:36496. doi: 10.1038/srep36496 (PMC5095602; doi:10.1038/srep36496)
Supplement: Supplementary Information [file srep36496-s1.pdf]

Supplementary Information

Diversity and function of the Antarctic krill microorganisms from *Euphausia superba*

Xiaoqiu Cui[1,2], Guoliang Zhu[1], Haishan Liu[1], Guoliang Jiang[3], Yi Wang[1,*] and Weiming Zhu[1,*]

1. Key Laboratory of Marine Drugs, Ministry of Education of China, School of Medicine and Pharmacy, Ocean University of China, Qingdao 266003, China
2. School of Pharmacy, Jining Medical University, Jining 272067, China
3. College of Marine Life, Ocean University of China, Qingdao 266003, China

[*]Correspondence. weimingzhu@ouc.edu.cn, wangyi0213@ouc.edu.cn.

List of Supplementary Information

Figure S1. The phylogenetic tree of strain OUCMDZ4115 from *E. superba*....S3
Figure S2. The phylogenetic tree of strain OUCMDZ4116 from *E. superba*....S4
Figure S3. The phylogenetic tree of strain OUCMDZ4117 from *E. superba*....S4
Figure S4. The phylogenetic tree of strain OUCMDZ4118 from *E. superba*....S5
Figure S5. The phylogenetic tree of strain OUCMDZ4120 from *E. superba*....S5
Figure S6. The phylogenetic tree of strain OUCMDZ4121 from *E. superba*....S6
Figure S7. The phylogenetic tree of strain OUCMDZ4122 from *E. superba*....S6
Figure S8. The phylogenetic tree of strain OUCMDZ4123 from *E. superba*....S7
Figure S9. The phylogenetic tree of strain OUCMDZ4124 from *E. superba*....S7
Figure S10. The phylogenetic tree of strain OUCMDZ4125 from *E. superba*....S8
Figure S11. The phylogenetic tree of strain OUCMDZ4126 from *E. superba*....S8
Figure S12. The phylogenetic tree of strain OUCMDZ4127 from *E. superba*....S9
Figure S13. The phylogenetic tree of strain OUCMDZ4129 from *E. superba*....S9
Figure S14. The phylogenetic tree of strain OUCMDZ4130 from *E. superba*.... S10
Figure S15. The phylogenetic tree of strain OUCMDZ4131 from *E. superba*....S10
Figure S16. The phylogenetic tree of strain OUCMDZ4132from *E. superba*.... S11
Figure S17. The phylogenetic tree of strain OUCMDZ4133 from *E. superba*....S11
Figure S18. The phylogenetic tree of strain OUCMDZ4134 from *E. superba*.... S12
Figure S19. The phylogenetic tree of strain OUCMDZ4136 from *E. superba*....S13
Figure S20. The phylogenetic tree of strain OUCMDZ4137 from *E. superba*.... S13
Figure S21. The phylogenetic tree of strain OUCMDZ4138 from *E. superba*....S14
Figure S22. The phylogenetic tree of strain OUCMDZ4139 from *E. superba*.... S14
Figure S23. The phylogenetic tree of strain OUCMDZ4140 from *E. superba*....S15
Figure S24. The phylogenetic tree of strain OUCMDZ4141 from *E. superba*.... S15
Figure S25. The phylogenetic tree of strain OUCMDZ4143 from *E. superba*....S16

Figure S26. The phylogenetic tree of strain OUCMDZ4145 from *E. superba*........ S16
Figure S27. The phylogenetic tree of strain OUCMDZ4149 from *E. superba*........ S17
Figure S28. The phylogenetic tree of strain OUCMDZ4150 from *E. superba*........ S17
Figure S29. The phylogenetic tree of strain OUCMDZ4151 from *E. superba*........ S18
Figure S30. The phylogenetic tree of strain OUCMDZ4152 from *E. superba*........ S18
Figure S31. The phylogenetic tree of strain OUCMDZ4154 from *E. superba*........ S19
Figure S32. The phylogenetic tree of strain OUCMDZ4155 from *E. superba*........ S19
Figure S33. The phylogenetic tree of strain OUCMDZ4156 from *E. superba*........ S20
Figure S34. The phylogenetic tree of strain OUCMDZ4161 from *E. superba*........ S20
Figure S35. The phylogenetic tree of strain OUCMDZ4162 from *E. superba*........ S21
Figure S36. The phylogenetic tree of strain OUCMDZ4163 from *E. superba*........ S21
Figure S37. The phylogenetic tree of strain OUCMDZ4164 from *E. superba*........ S22
Figure S38. The phylogenetic tree of strain OUCMDZ4166 from *E. superba*........ S22
Figure S39. The phylogenetic tree of strain OUCMDZ4167 from *E. superba*........ S23
Figure S40. The phylogenetic tree of strain OUCMDZ4168 from *E. superba*........ S23
Figure S41. The phylogenetic tree of strain OUCMDZ4169 from *E. superba*........ S24
Figure S42. The phylogenetic tree of strain OUCMDZ4170 from *E. superba*........ S24
Figure S43. The phylogenetic tree of strain OUCMDZ4173 from *E. superba*........ S25
Figure S44. The phylogenetic tree of strain OUCMDZ4174 from *E. superba*........ S25
Figure S45. The phylogenetic tree of strain OUCMDZ4176 from *E. superba*........ S26
Figure S46. The phylogenetic tree of strain OUCMDZ4177 from *E. superba*........ S26
Figure S47. The phylogenetic tree of strain OUCMDZ4179 from *E. superba*........ S27
Figure S48. The phylogenetic tree of strain OUCMDZ4181 from *E. superba*........ S27
Figure S49. The phylogenetic tree of strain OUCMDZ4182 from *E. superba*........ S28
Figure S50. The phylogenetic tree of strain OUCMDZ4183 from *E. superba*........ S28
Figure S51. The phylogenetic tree of strain OUCMDZ4185 from *E. superba*........ S29
Figure S52. The phylogenetic tree of strain OUCMDZ4186 from *E. superba*........ S29
Figure S53. The phylogenetic tree of strain OUCMDZ4187 from *E. superba*........ S30
Figure S54. The phylogenetic tree of strain OUCMDZ4188 from *E. superba*........ S30
Figure S55. The phylogenetic tree of strain OUCMDZ4189 from *E. superba*........ S31
Figure S56. The phylogenetic tree of strain OUCMDZ4190 from *E. superba*........ S31
Figure S57. The phylogenetic tree of strain OUCMDZ4191 from *E. superba*........ S32
Figure S58. The phylogenetic tree of strain OUCMDZ4192 from *E. superba*........ S32
Figure S59. The phylogenetic tree of strain OUCMDZ4199 from *E. superba*........ S33
Figure S60. The phylogenetic tree of strain OUCMDZ4202 from *E. superba*........ S33
Figure S61. The phylogenetic tree of strain OUCMDZ4205 from *E. superba*........ S34
Figure S62. The phylogenetic tree of strain OUCMDZ4206 from *E. superba*........ S34
Figure S63. The phylogenetic tree of strain OUCMDZ4207 from *E. superba*........ S35
Figure S64. The phylogenetic tree of strain OUCMDZ4208 from *E. superba*........ S35
Figure S65. The phylogenetic tree of strain OUCMDZ4210 from *E. superba*........ S36
Figure S66. The phylogenetic tree of strain OUCMDZ4211 from *E. superba*........ S36
Figure S67. The phylogenetic tree of strain OUCMDZ4212 from *E. superba*........ S37
Figure S68. The phylogenetic tree of strain OUCMDZ4213 from *E. superba*........ S37
Figure S69. The phylogenetic tree of strain OUCMDZ4214 from *E. superba*........ S38

Figure S70. The phylogenetic tree of strain OUCMDZ4215 from *E. superba*........ S38
Figure S71. The phylogenetic tree of strain OUCMDZ4218 from *E. superba*........ S39
Figure S72. The phylogenetic tree of strain OUCMDZ4219 from *E. superba*........ S39
Figure S73. The phylogenetic tree of strain OUCMDZ4220 from *E. superba*........ S40
Figure S74. The phylogenetic tree of strain OUCMDZ4221 from *E. superba*........ S40
Figure S75. The phylogenetic tree of strain OUCMDZ4222 from *E. superba*........ S41
Figure S76. The HPLC profile of the EtOAc extracts from the cultures of Penicillium citrinum OUCMDZ4136...S41
Figure S77. Sampling site of *E. superba*........ S42

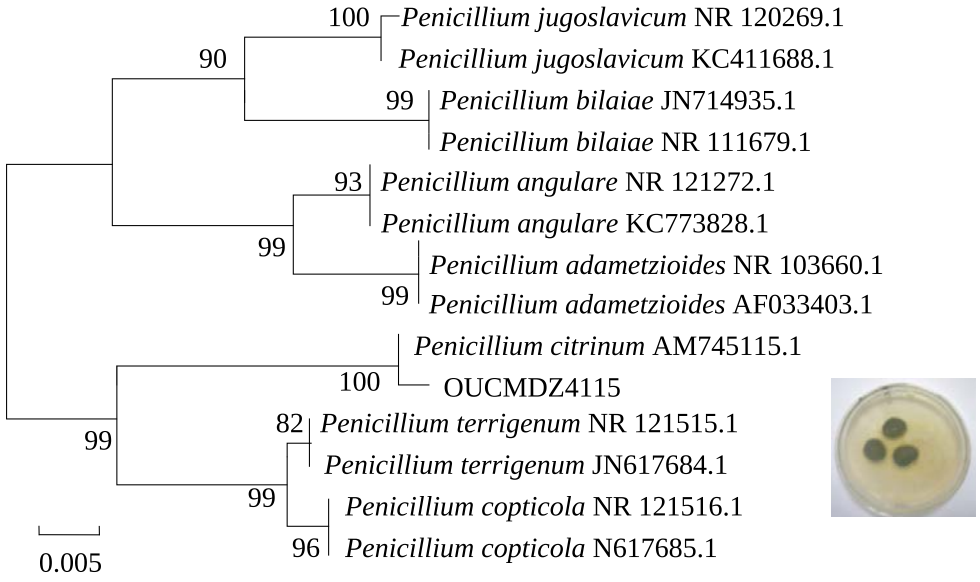

Figure S1. Neighbor-joining of phylogenetic tree of strain OUCMDZ4115 from *E. superba* based on 18S rRNA gene sequences. The numbers on each node represent the bootstrap value from 1000 replicates and the scale bar represents 0.005 substitutions per nucleotide.

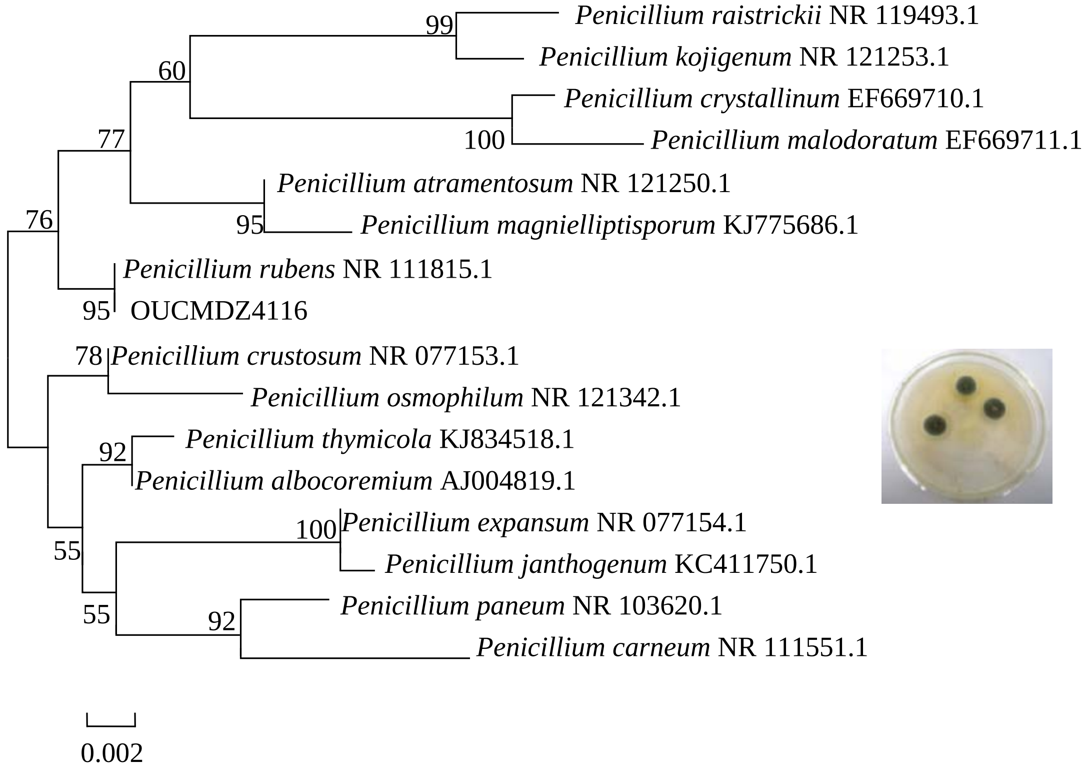

Figure S2. Neighbor-joining of phylogenetic tree of strain OUCMDZ4116 from *E. superba* based on 18S rRNA gene sequences. The numbers on each node represent the bootstrap value from 1000 replicates and the scale bar represents 0.002 substitutions per nucleotide.

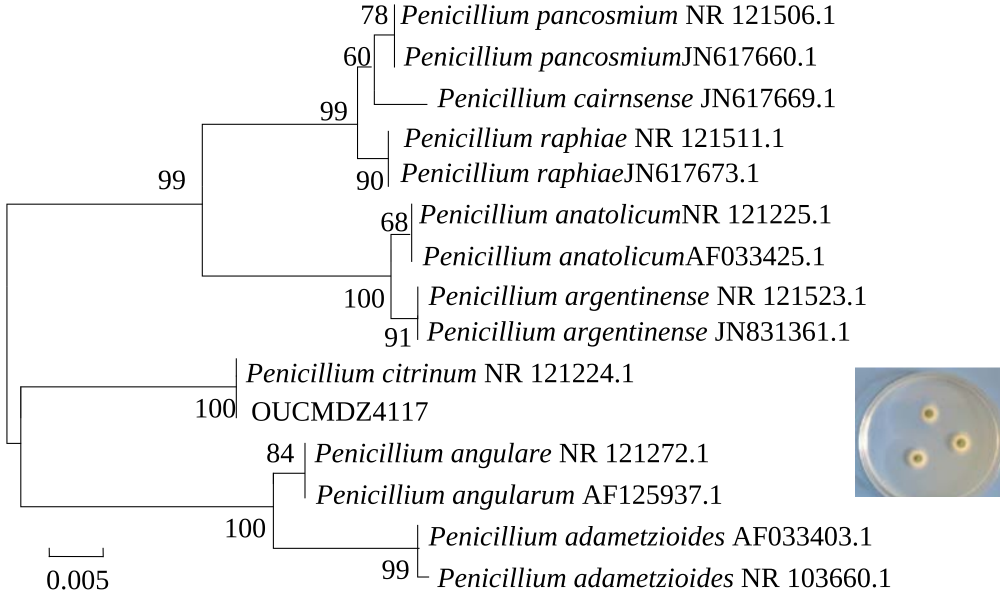

Figure S3. Neighbor-joining of phylogenetic tree of strain OUCMDZ4117 isolates from *E. superba* based on 18S rRNA gene sequences. The numbers on each node represent the bootstrap value from 1000 replicates and the scale bar represents 0.005 substitutions per nucleotide.

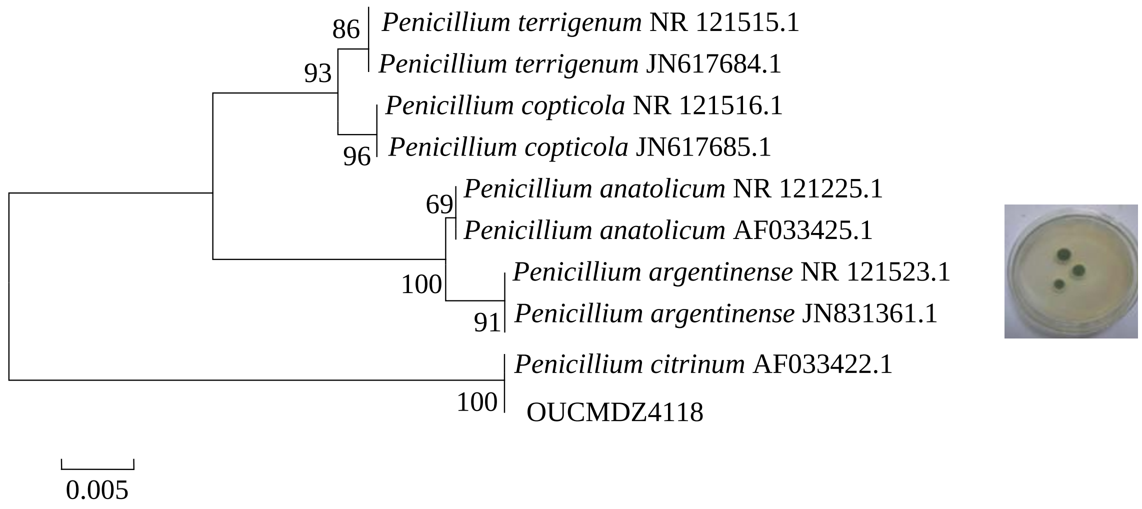

Figure S4. Neighbor-joining of phylogenetic tree of strain OUCMDZ4118 from *E. superba* based on 18S rRNA gene sequences. The numbers on each node represent the bootstrap value from 1000 replicates and the scale bar represents 0.005 substitutions per nucleotide.

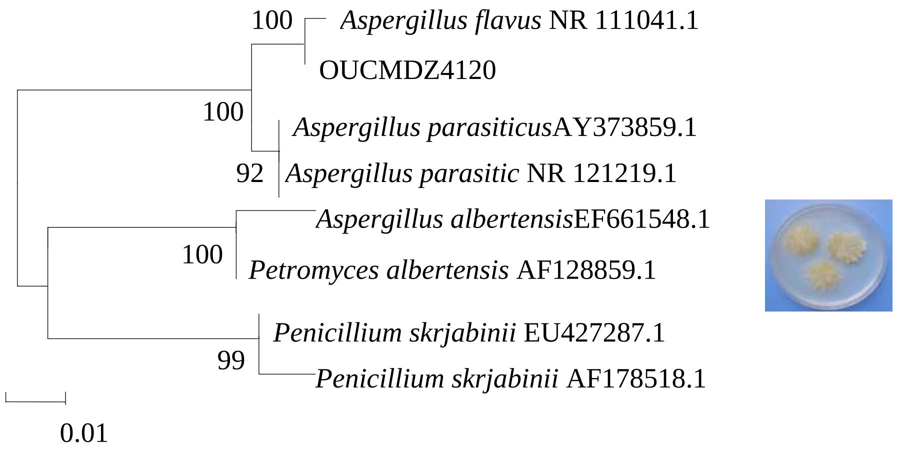

Figure S5. Neighbor-joining of phylogenetic tree of strain OUCMDZ4120 from *E. superba* based on 18S rRNA gene sequences. The numbers on each node represent the bootstrap value from 1000 replicates and the scale bar represents 0.01 substitutions per nucleotide.

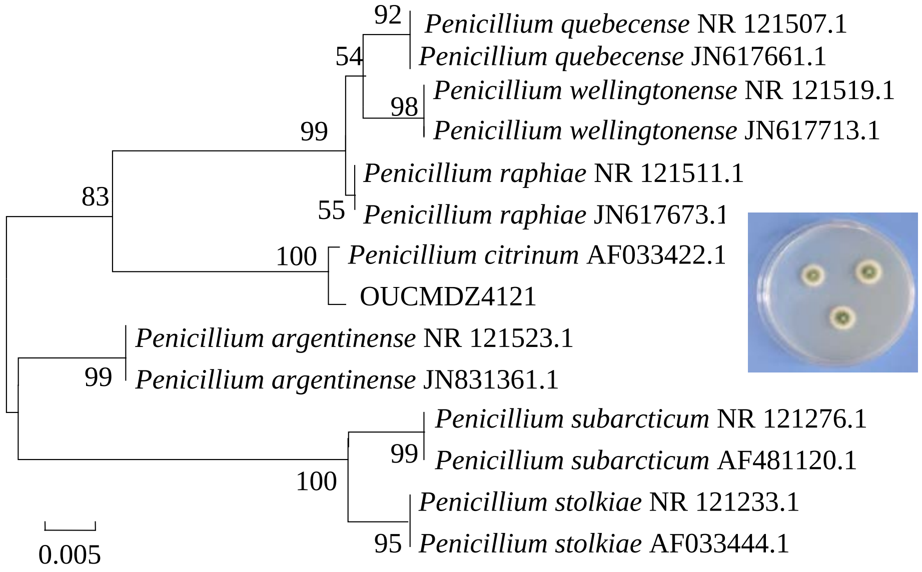

Figure S6. Neighbor-joining of phylogenetic tree of strain OUCMDZ4121 from *E. superba* based on 18S rRNA gene sequences. The numbers on each node represent the bootstrap value from 1000 replicates and the scale bar represents 0.005 substitutions per nucleotide.

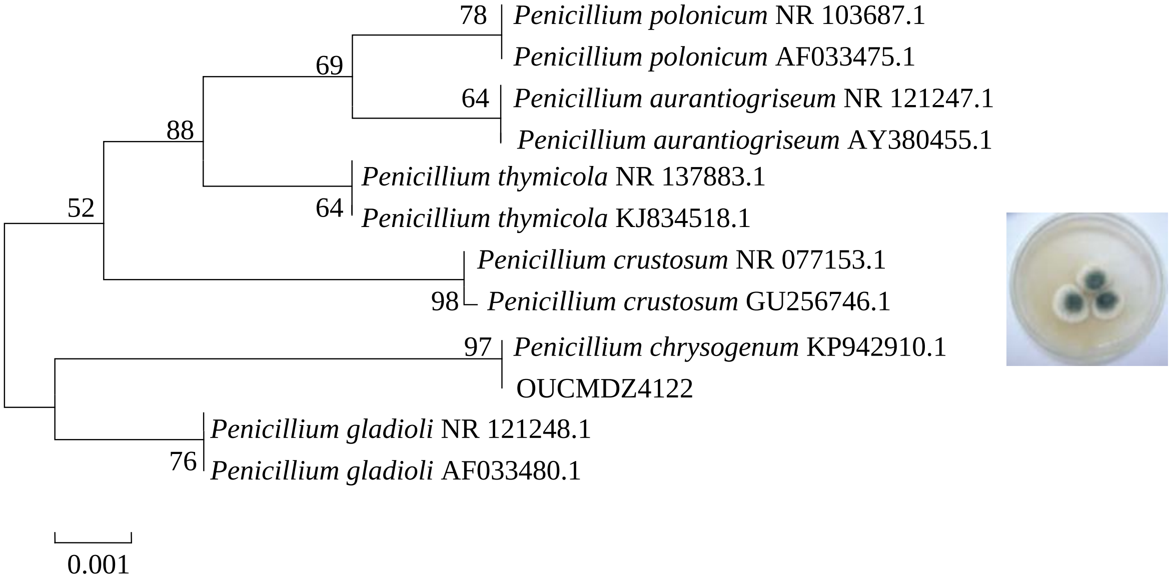

Figure S7. Neighbor-joining of phylogenetic tree of strain OUCMDZ4122 from *E. superba* based on 18S rRNA gene sequences. The numbers on each node represent the bootstrap value from 1000 replicates and the scale bar represents 0.1 substitutions per nucleotide.

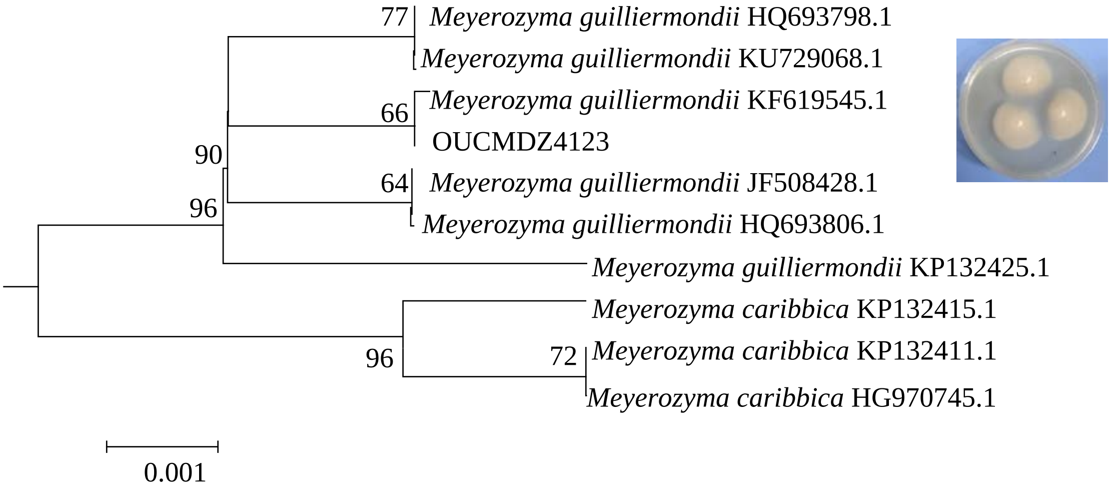

Figure S8. Neighbor-joining of phylogenetic tree of strain OUCMDZ4123 from *E. superba* based on 18S rRNA gene sequences. The numbers on each node represent the bootstrap value from 1000 replicates and the scale bar represents 0.001 substitutions per nucleotide.

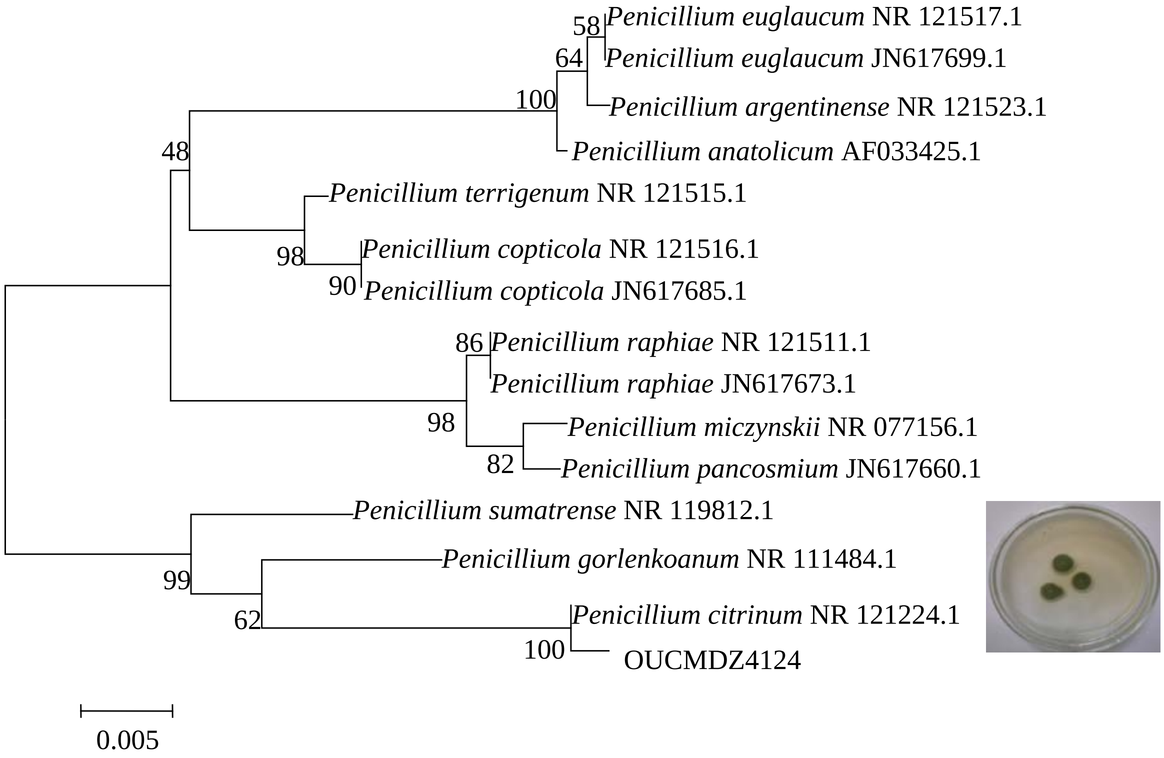

Figure S9. Neighbor-joining of phylogenetic tree of strain OUCMDZ4124 from *E. superba* based on 18S rRNA gene sequences. The numbers on each node represent the bootstrap value from 1000 replicates and the scale bar represents 0.005 substitutions per nucleotide.

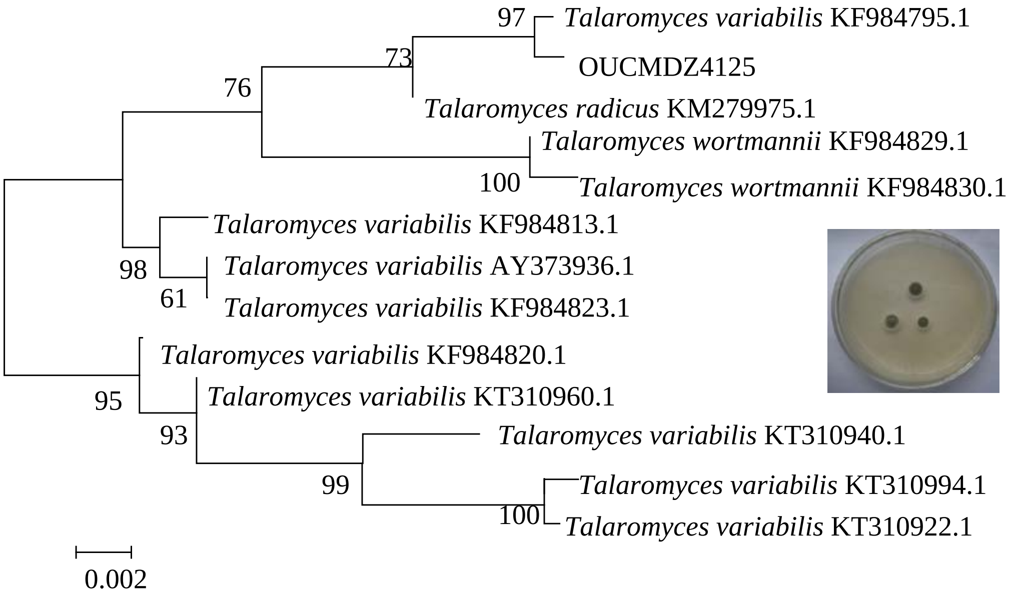

Figure S10. Neighbor-joining of phylogenetic tree of strain OUCMDZ4125 from *E. superba* based on 18S rRNA gene sequences. The numbers on each node represent the bootstrap value from 1000 replicates and the scale bar represents 0.002 substitutions per nucleotide.

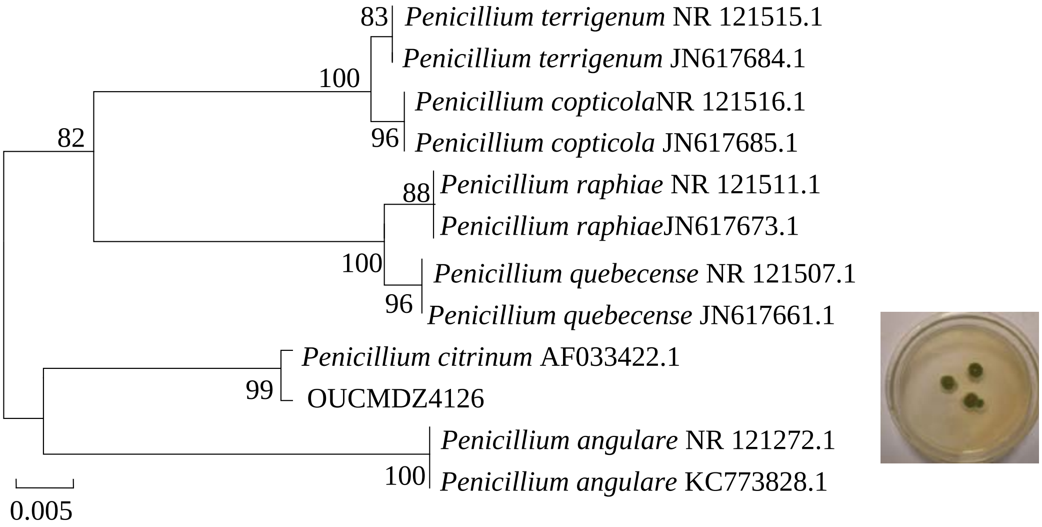

Figure S11. Neighbor-joining of phylogenetic tree of strain OUCMDZ4126 from *E. superba* based on 18S rRNA gene sequences. The numbers on each node represent the bootstrap value from 1000 replicates and the scale bar represents 0.005 substitutions per nucleotide.

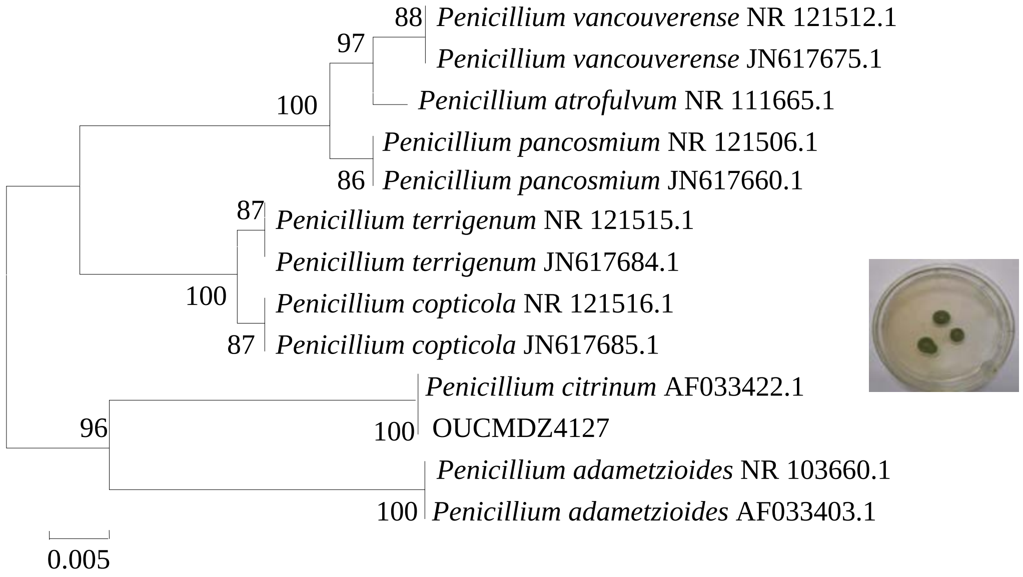

Figure S12. Neighbor-joining of phylogenetic tree of strain 7890-OUCMDZ4127 from *E. superba* based on 18S rRNA gene sequences. The numbers on each node represent the bootstrap value from 1000 replicates and the scale bar represents 0.005 substitutions per nucleotide.

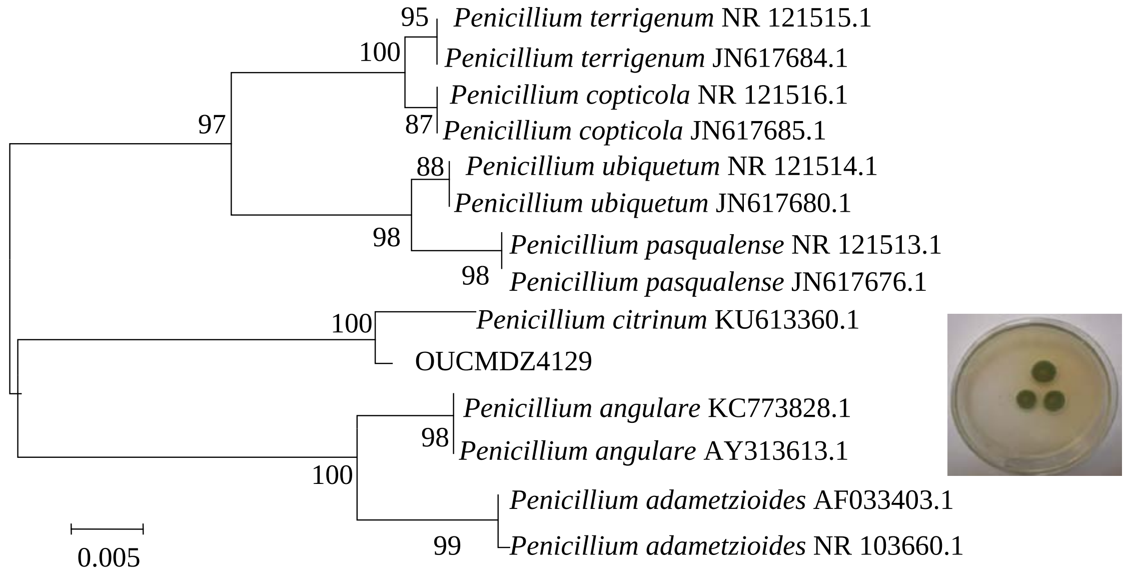

Figure S13. Neighbor-joining of phylogenetic tree of strain OUCMDZ4129 from *E. superba* based on 18S rRNA gene sequences. The numbers on each node represent the bootstrap value from 1000 replicates and the scale bar represents 0.005 substitutions per nucleotide.

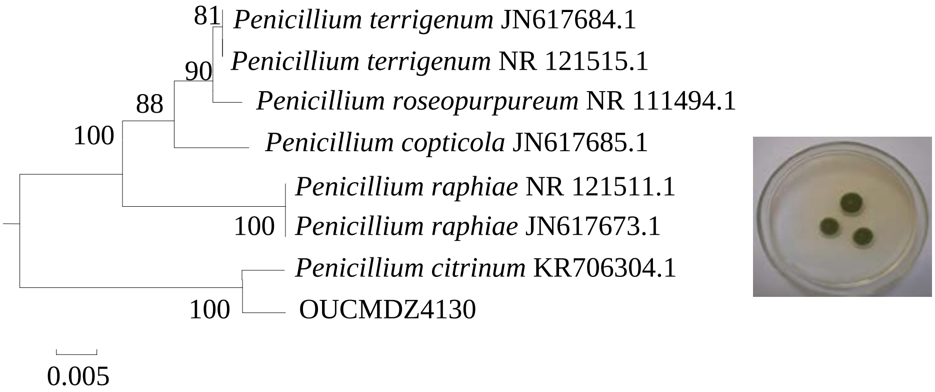

Figure S14. Neighbor-joining of phylogenetic tree of strain OUCMDZ4130 from *E. superba* based on 18S rRNA gene sequences. The numbers on each node represent the bootstrap value from 1000 replicates and the scale bar represents 0.005 substitutions per nucleotide.

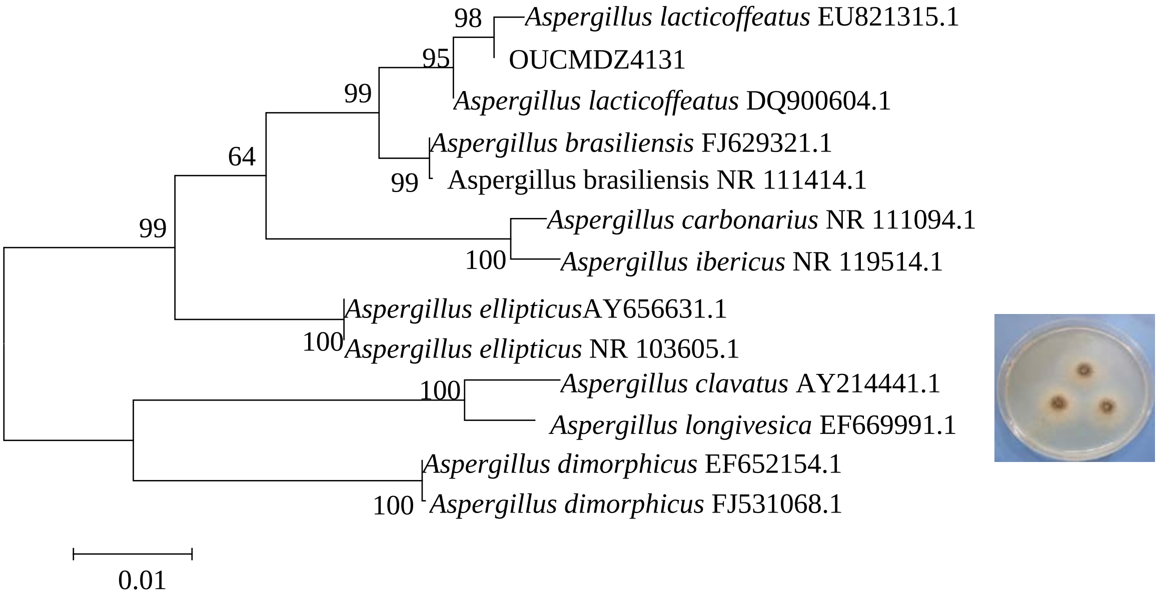

Figure S15. Neighbor-joining of phylogenetic tree of strain OUCMDZ4131 from *E. superba* based on 18S rRNA gene sequences. The numbers on each node represent the bootstrap value from 1000 replicates and the scale bar represents 0.01 substitutions per nucleotide.

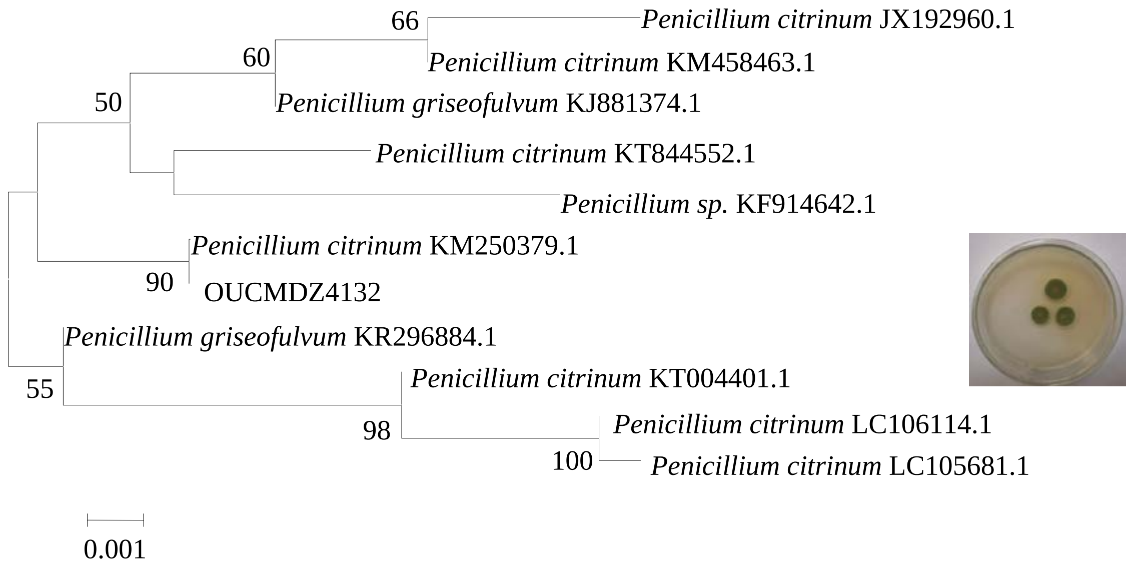

Figure S16. Neighbor-joining of phylogenetic tree of strain OUCMDZ4132 from *E. superba* based on 18S rRNA gene sequences. The numbers on each node represent the bootstrap value from 1000 replicates and the scale bar represents 0.001 substitutions per nucleotide.

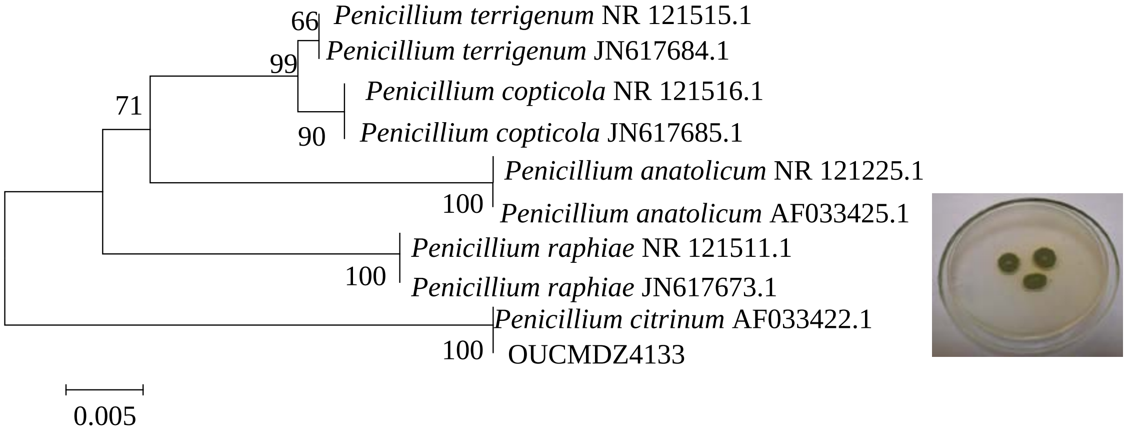

Figure S17. Neighbor-joining of phylogenetic tree of strain OUCMDZ4133 from *E. superba* based on 18S rRNA gene sequences. The numbers on each node represent the bootstrap value from 1000 replicates and the scale bar represents 0.005 substitutions per nucleotide.

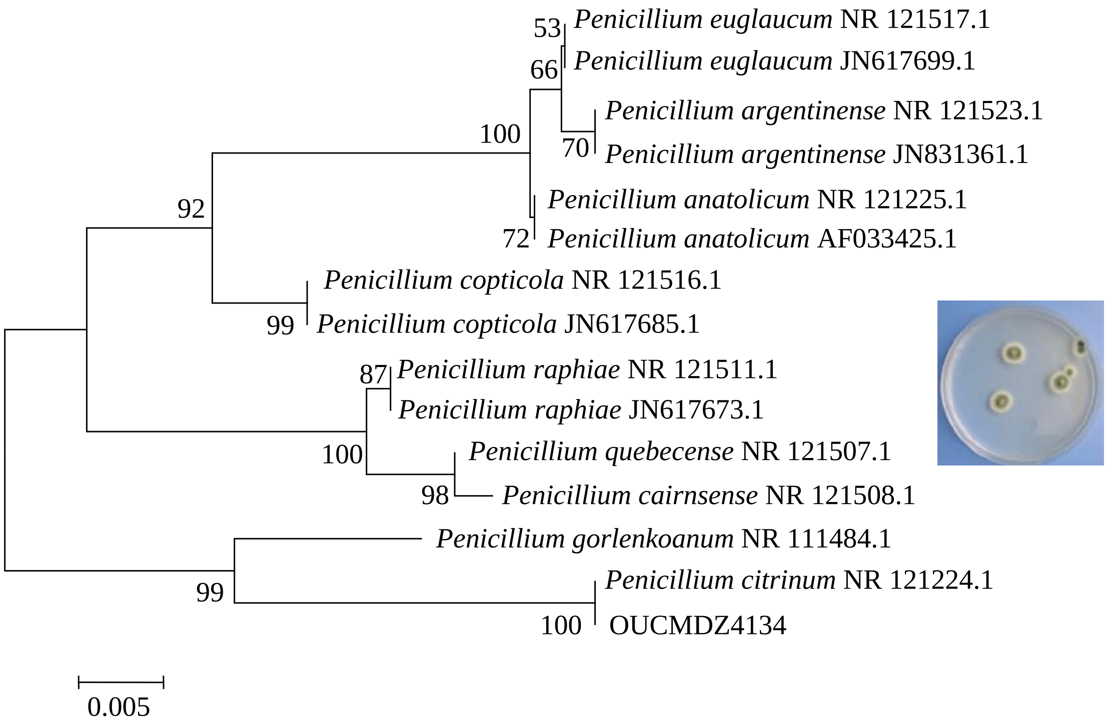

Figure S18. Neighbor-joining of phylogenetic tree of strain OUCMDZ4134 from *E. superba* based on 18S rRNA gene sequences. The numbers on each node represent the bootstrap value from 1000 replicates and the scale bar represents 0.005 substitutions per nucleotide.

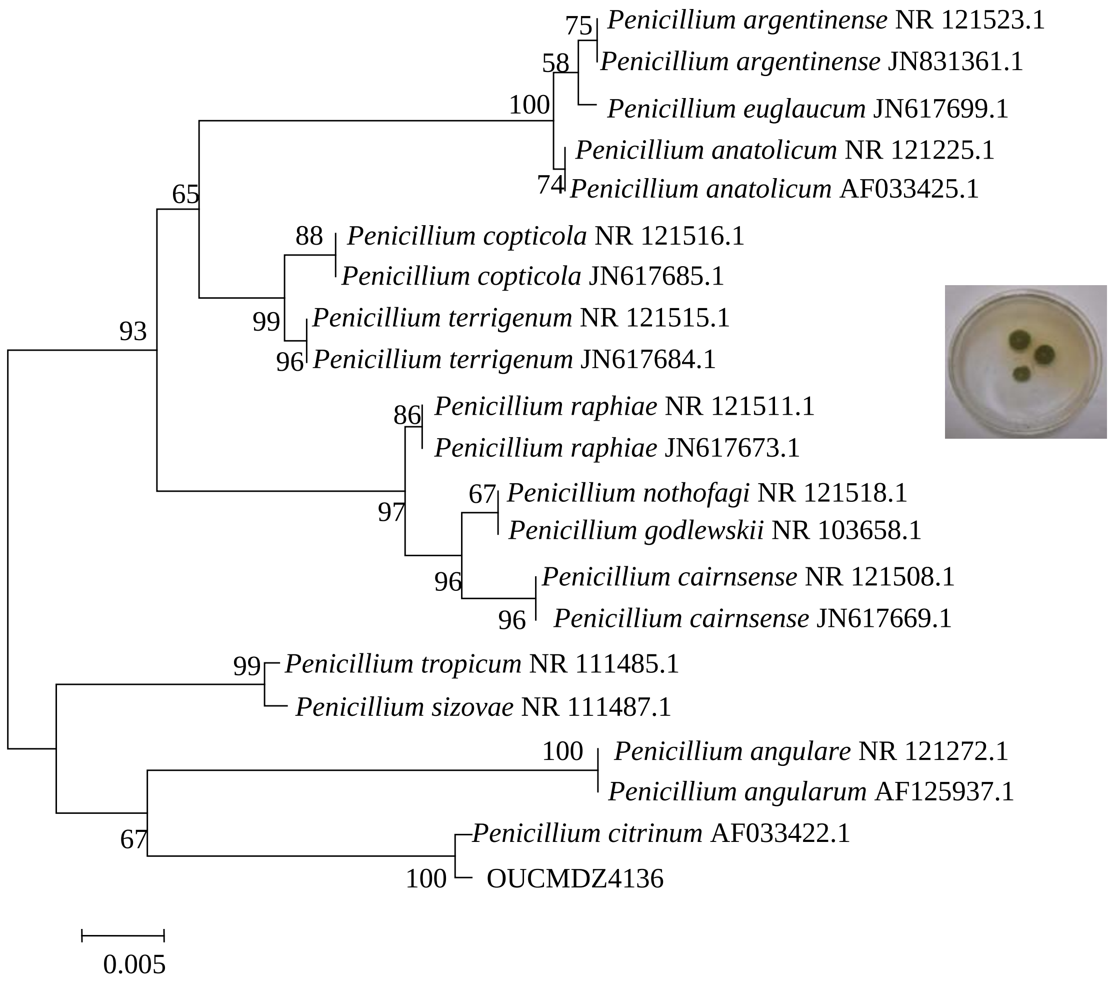

Figure S19. Neighbor-joining of phylogenetic tree of strain OUCMDZ4136 from *E. superba* based on 18S rRNA gene sequences. The numbers on each node represent the bootstrap value from 1000 replicates and the scale bar represents 0.005 substitutions per nucleotide.

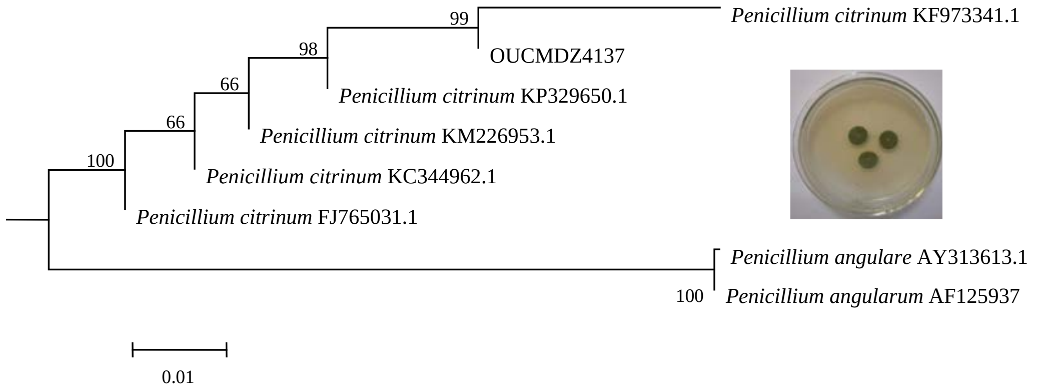

Figure S20. Neighbor-joining of phylogenetic tree of strain OUCMDZ4137 from *E. superba* based on 18S rRNA gene sequences. The numbers on each node represent the bootstrap value from 1000 replicates and the scale bar represents 0.01 substitutions per nucleotide.

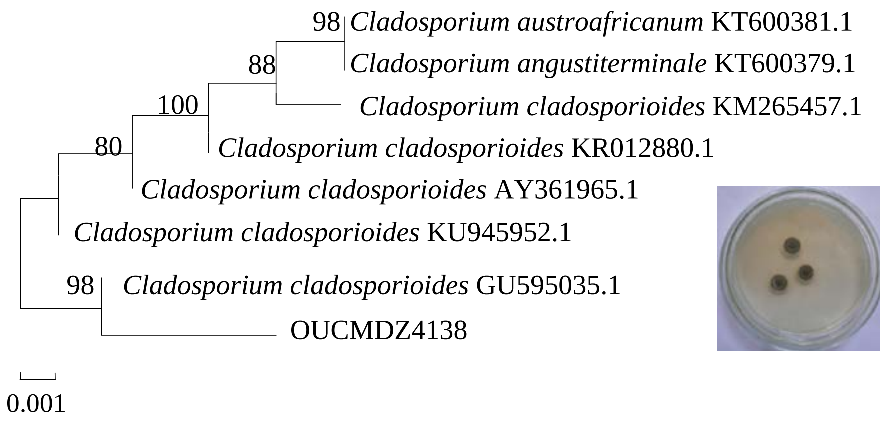

Figure S21. Neighbor-joining of phylogenetic tree of strain OUCMDZ4138 from *E. superba* based on 18S rRNA gene sequences. The numbers on each node represent the bootstrap value from 1000 replicates and the scale bar represents 0.001 substitutions per nucleotide.

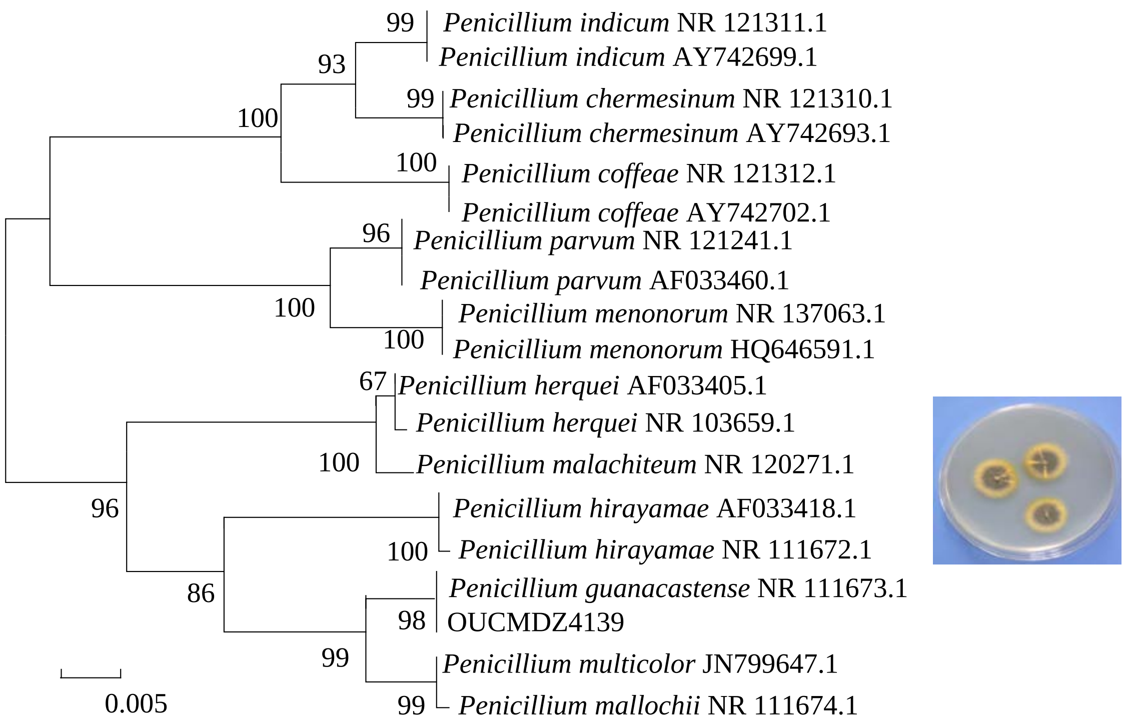

Figure S22. Neighbor-joining of phylogenetic tree of strain OUCMDZ4139 from *E. superba* based on 18S rRNA gene sequences. The numbers on each node represent the bootstrap value from 1000 replicates and the scale bar represents 0.005 substitutions per nucleotide.

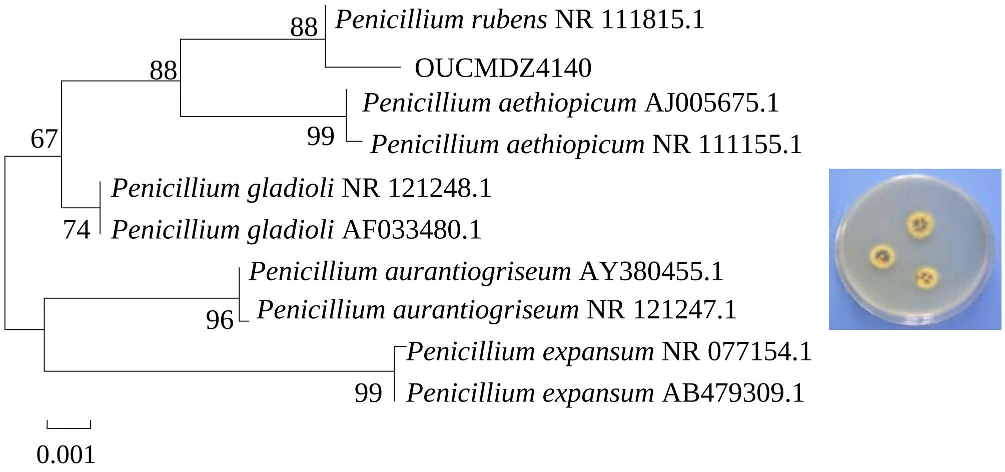

Figure S23. Neighbor-joining of phylogenetic tree of strain OUCMDZ4140 from *E. superba* based on 18S rRNA gene sequences. The numbers on each node represent the bootstrap value from 1000 replicates and the scale bar represents 0.001 substitutions per nucleotide.

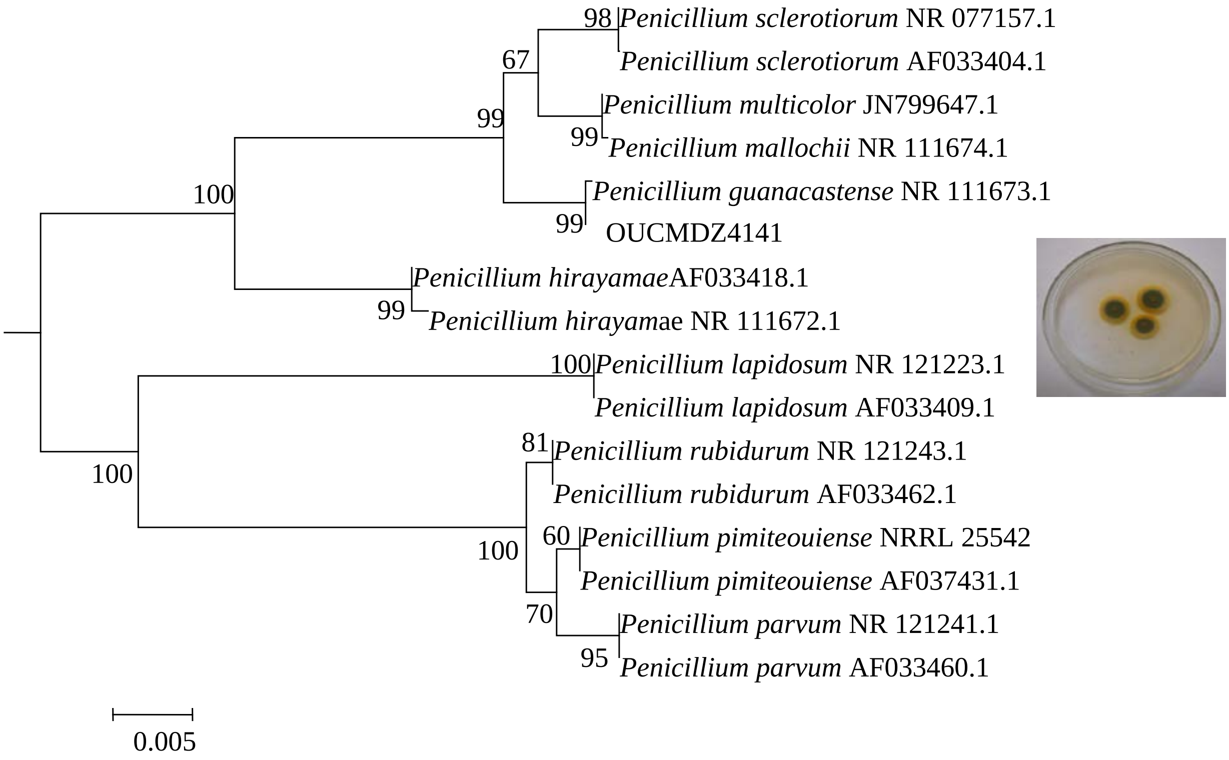

Figure S24. Neighbor-joining of phylogenetic tree of strain OUCMDZ4141 from *E. superba* based on 18S rRNA gene sequences. The numbers on each node represent the bootstrap value from 1000 replicates and the scale bar represents 0.005 substitutions per nucleotide.

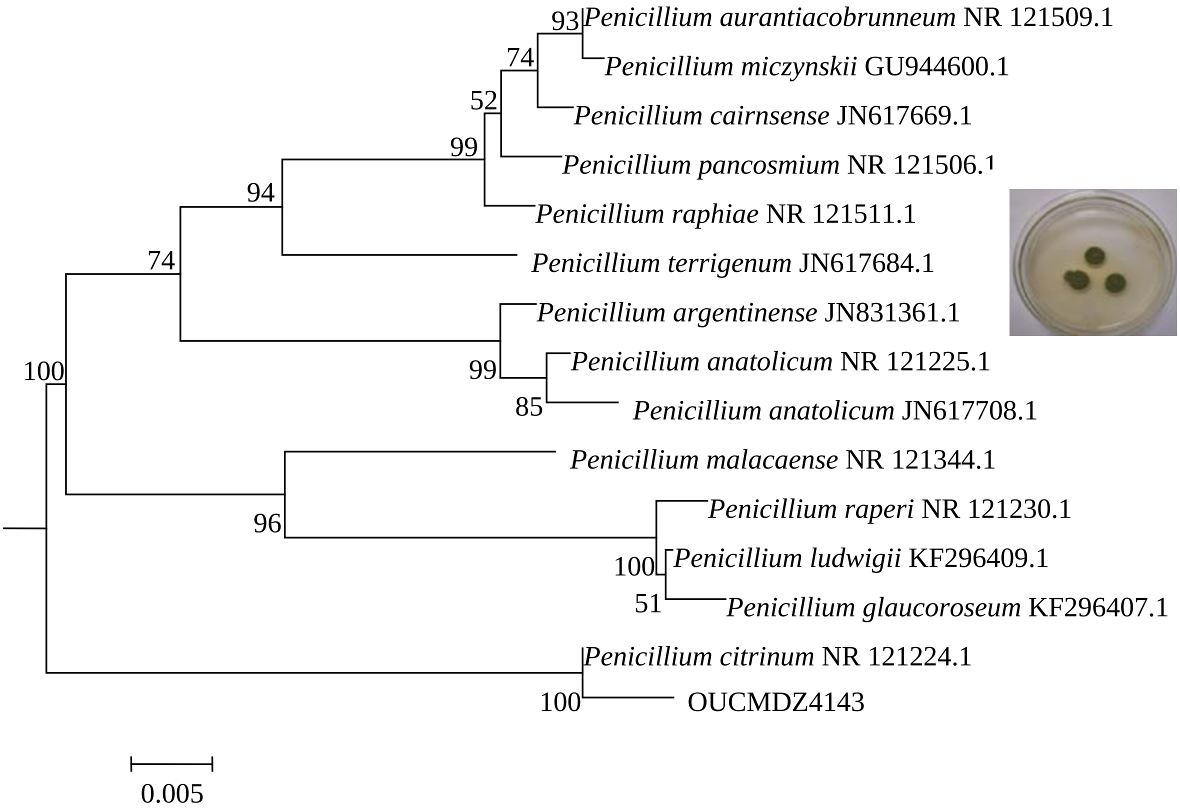

Figure S25. Neighbor-joining of phylogenetic tree of strain OUCMDZ4143 from *E. superba* based on 18S rRNA gene sequences. The numbers on each node represent the bootstrap value from 1000 replicates and the scale bar represents 0.005 substitutions per nucleotide.

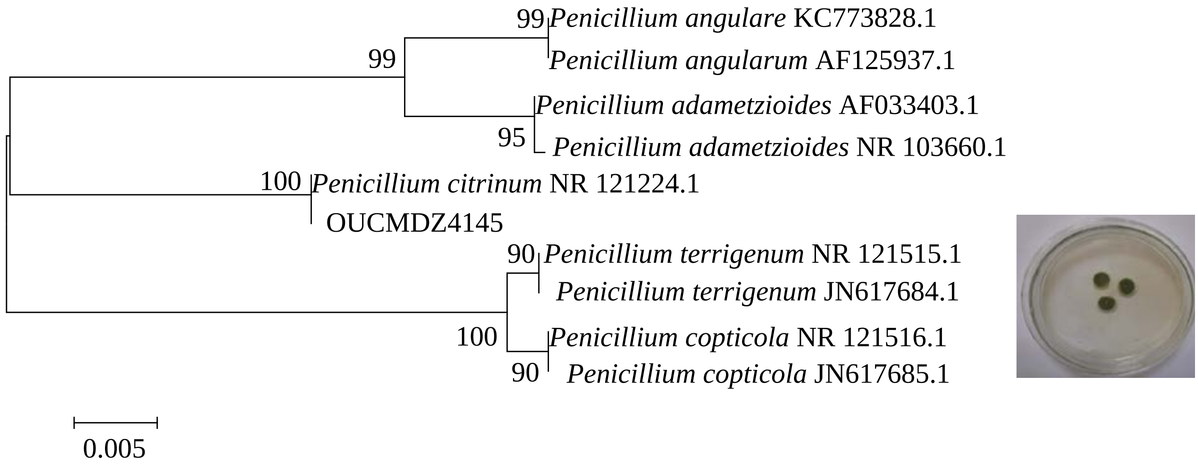

Figure S26. Neighbor-joining of phylogenetic tree of strain OUCMDZ4145 isolates from *E. superba* based on 18S rRNA gene sequences. The numbers on each node represent the bootstrap value from 1000 replicates and the scale bar represents 0.005 substitutions per nucleotide.

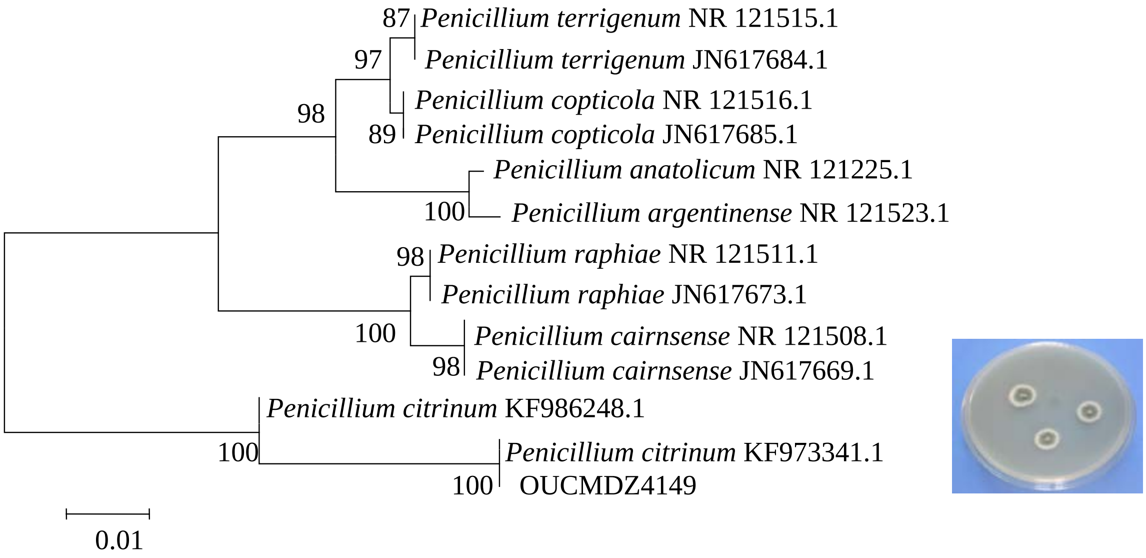

Figure S27. Neighbor-joining of phylogenetic tree of strain OUCMDZ4149 from *E. superba* based on 18S rRNA gene sequences. The numbers on each node represent the bootstrap value from 1000 replicates and the scale bar represents 0.01 substitutions per nucleotide.

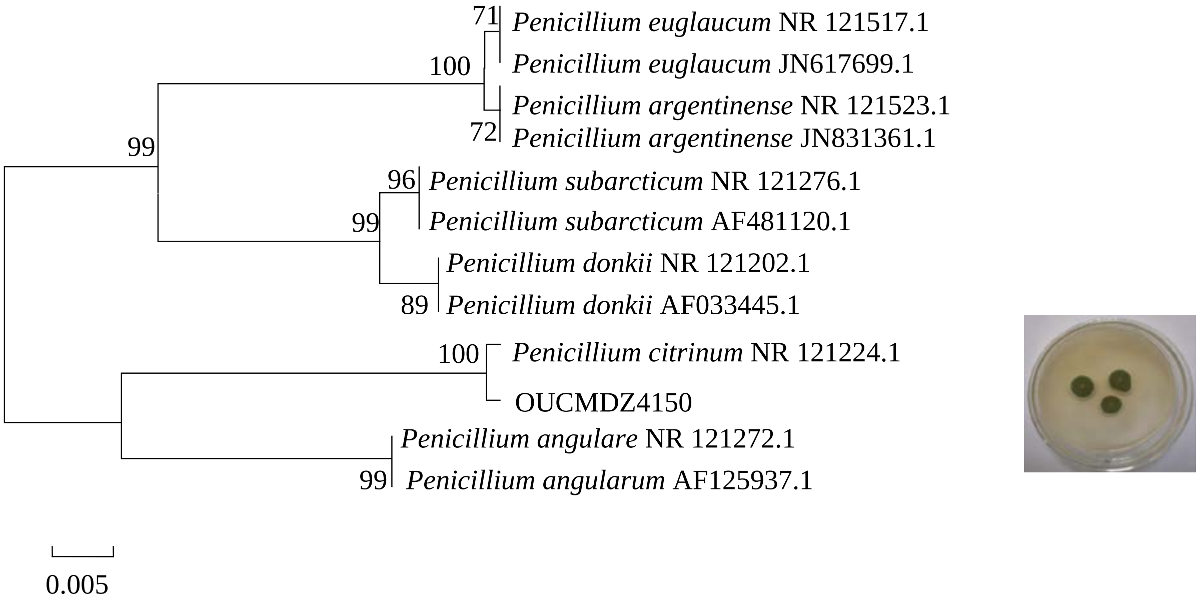

Figure S28. Neighbor-joining of phylogenetic tree of strain OUCMDZ4150 isolates from *E. superba* based on 18S rRNA gene sequences. The numbers on each node represent the bootstrap value from 1000 replicates and the scale bar represents 0.05 substitutions per nucleotide.

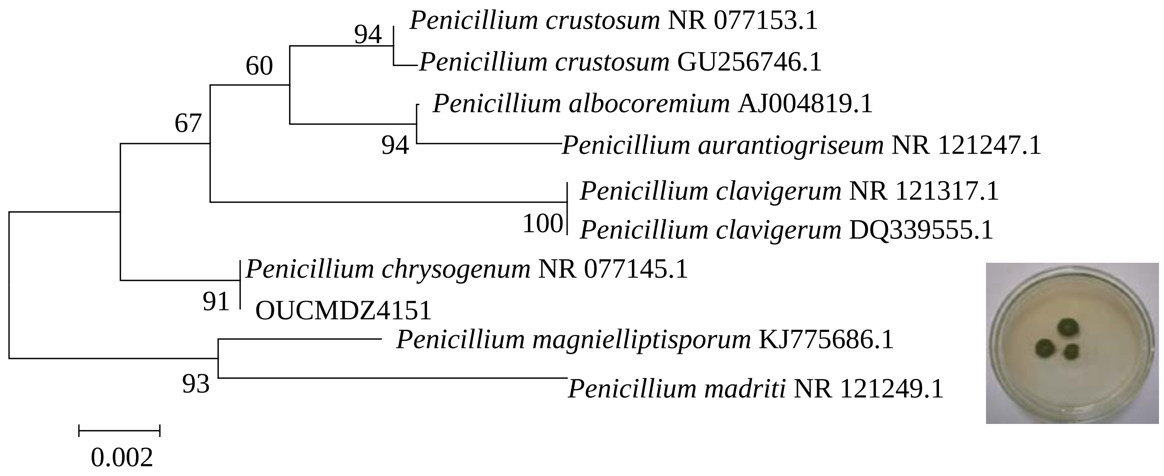

Figure S29. Neighbor-joining of phylogenetic tree of strain OUCMDZ4151 from *E. superba* based on 18S rRNA gene sequences. The numbers on each node represent the bootstrap value from 1000 replicates and the scale bar represents 0.002 substitutions per nucleotide.

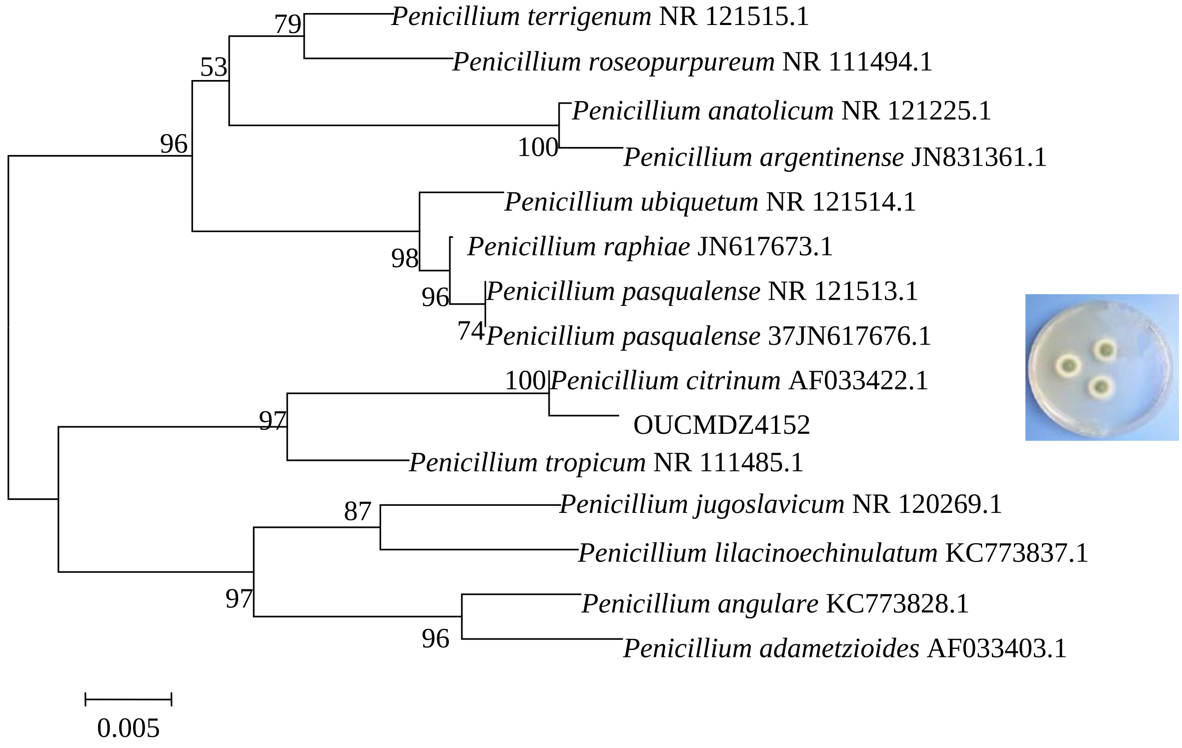

Figure S30. Neighbor-joining of phylogenetic tree of strain OUCMDZ4152 from *E. superba* based on 18S rRNA gene sequences. The numbers on each node represent the bootstrap value from 1000 replicates and the scale bar represents 0.005 substitutions per nucleotide.

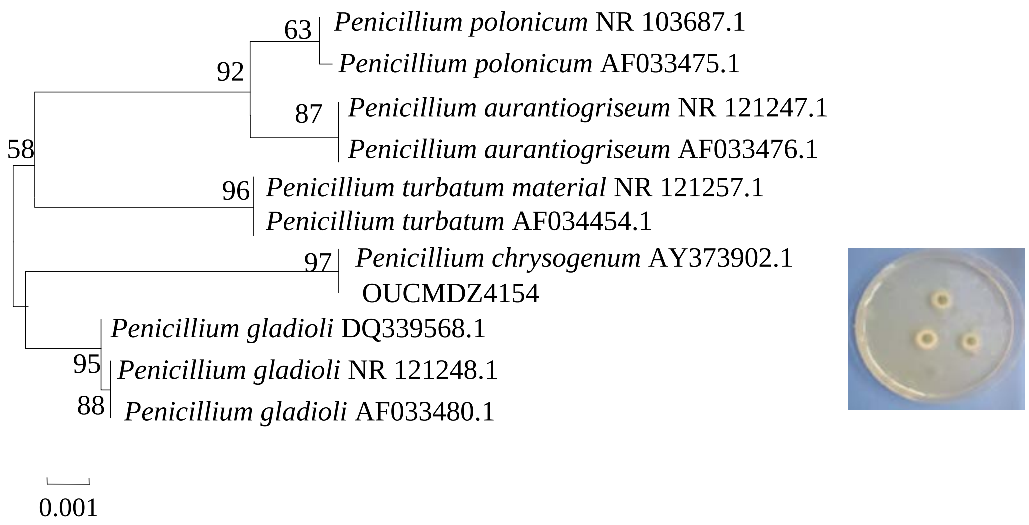

Figure S31. Neighbor-joining of phylogenetic tree of strain OUCMDZ4154 from *E. superba* based on 18S rRNA gene sequences. The numbers on each node represent the bootstrap value from 1000 replicates and the scale bar represents 0.001 substitutions per nucleotide.

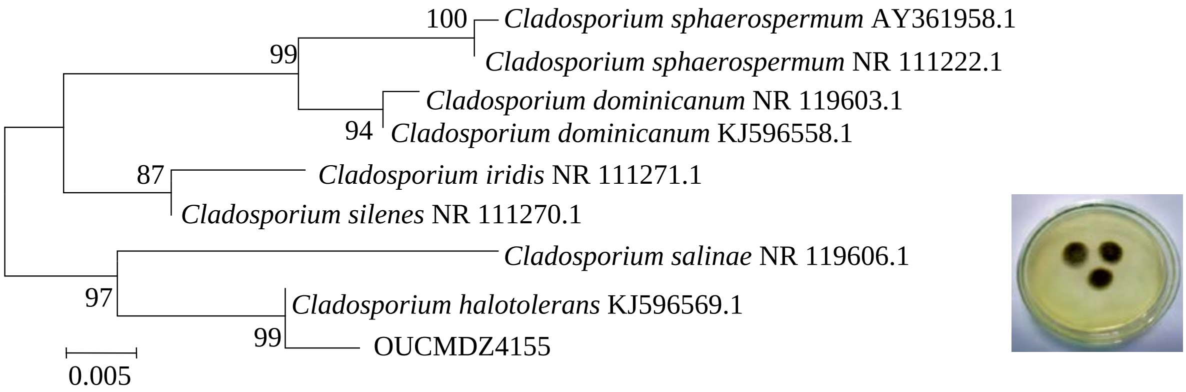

Figure S32. Neighbor-joining of phylogenetic tree of strain OUCMDZ4155 from *E. superba* based on 18S rRNA gene sequences. The numbers on each node represent the bootstrap value from 1000 replicates and the scale bar represents 0.005 substitutions per nucleotide.

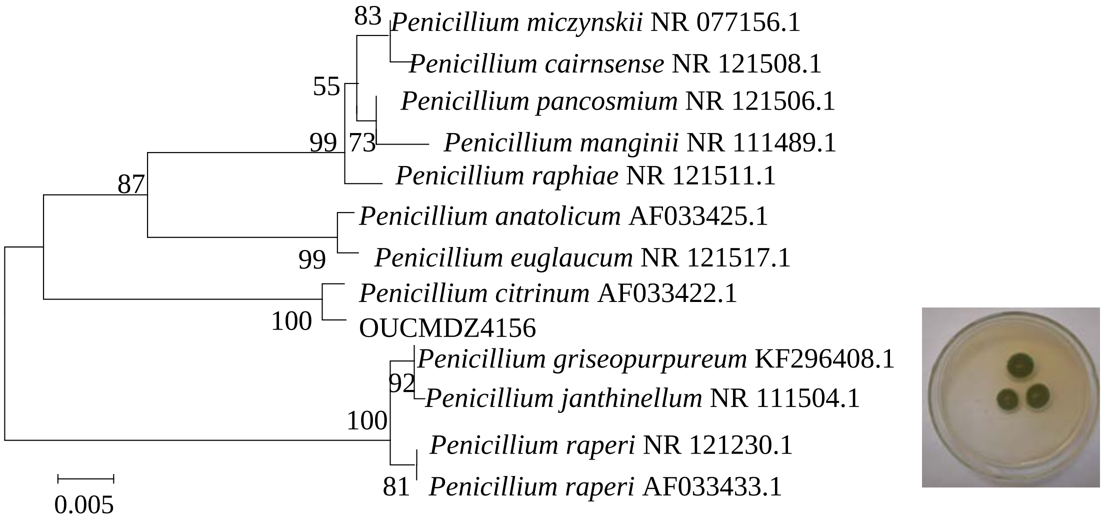

Figure S33. Neighbor-joining of phylogenetic tree of strain OUCMDZ4156 from *E. superba* based on 18S rRNA gene sequences. The numbers on each node represent the bootstrap value from 1000 replicates and the scale bar represents 0.005 substitutions per nucleotide.

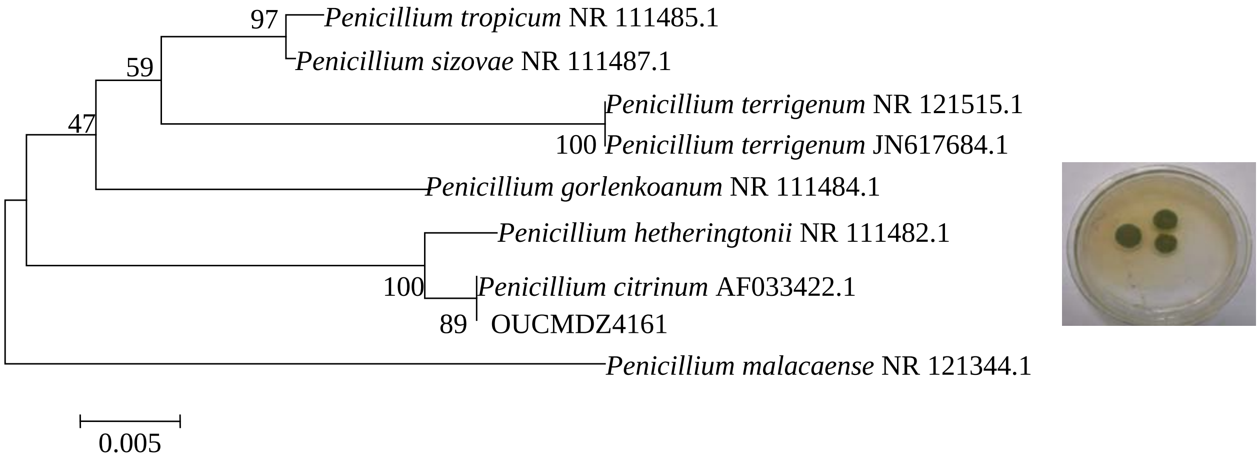

Figure S34. Neighbor-joining of phylogenetic tree of strain OUCMDZ4161 from *E. superba* based on 18S rRNA gene sequences. The numbers on each node represent the bootstrap value from 1000 replicates and the scale bar represents 0.005 substitutions per nucleotide.

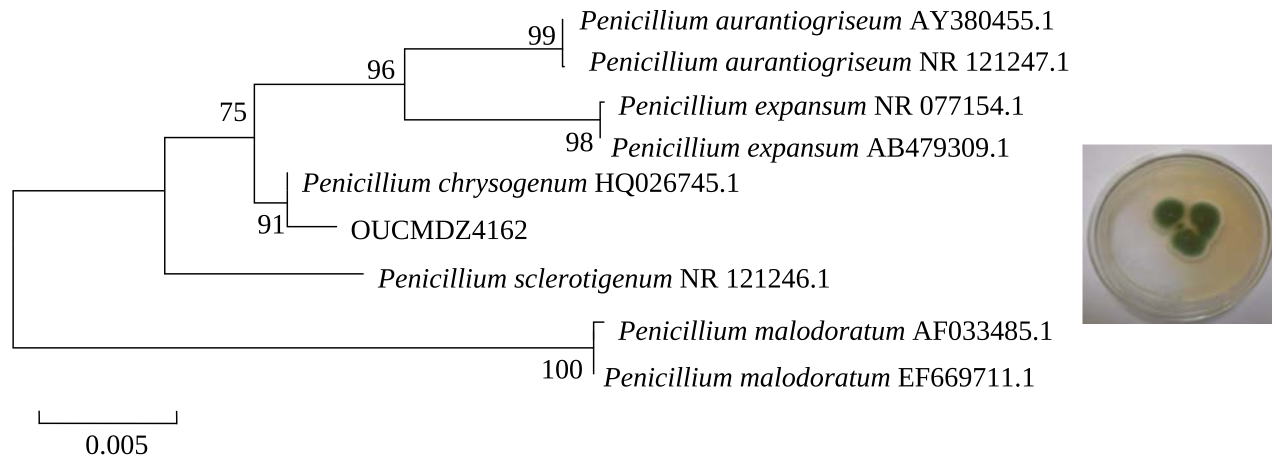

Figure S35. Neighbor-joining of phylogenetic tree of strain OUCMDZ4162 from *E. superba* based on 18S rRNA gene sequences. The numbers on each node represent the bootstrap value from 1000 replicates and the scale bar represents 0.005 substitutions per nucleotide.

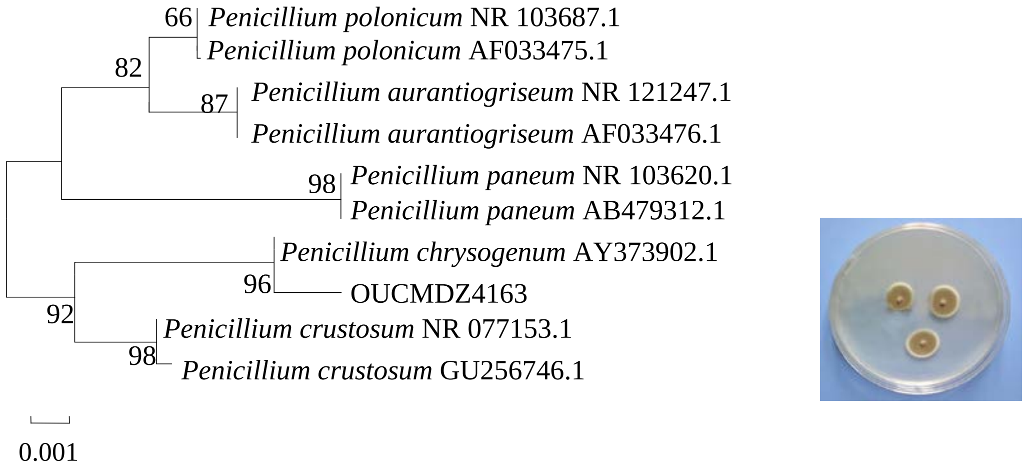

Figure S36. Neighbor-joining of phylogenetic tree of strain OUCMDZ4163 from *E. superba* based on 18S rRNA gene sequences. The numbers on each node represent the bootstrap value from 1000 replicates and the scale bar represents 0.001 substitutions per nucleotide.

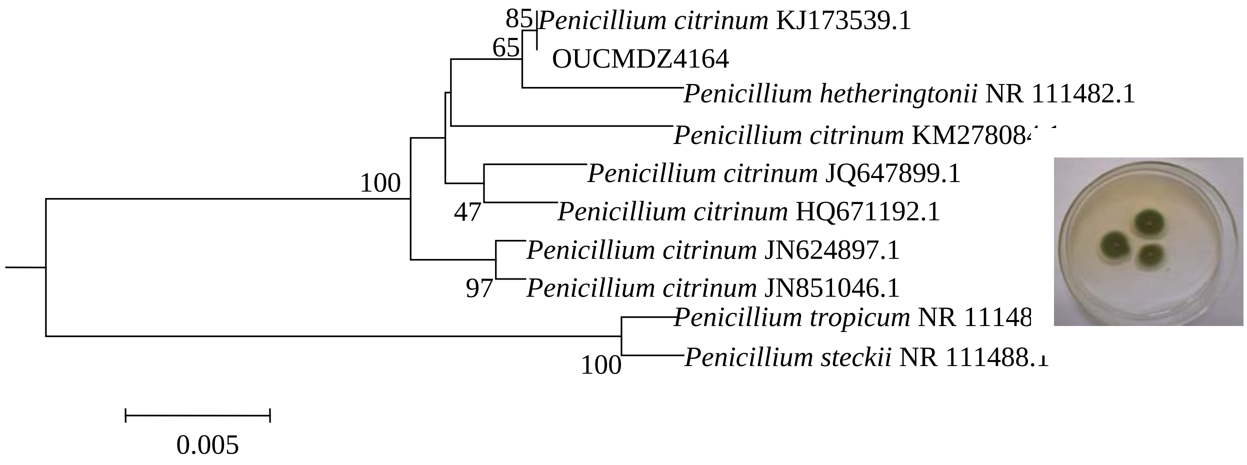

Figure S37. Neighbor-joining of phylogenetic tree of strain OUCMDZ4164 from *E. superba* based on 18S rRNA gene sequences. The numbers on each node represent the bootstrap value from 1000 replicates and the scale bar represents 0.005 substitutions per nucleotide.

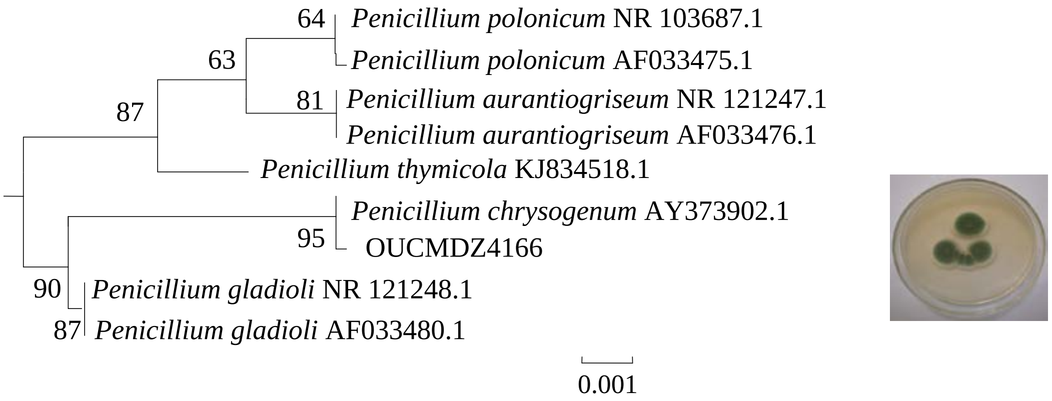

Figure S38. Neighbor-joining of phylogenetic tree of strain OUCMDZ4166 from *E. superba* based on 18S rRNA gene sequences. The numbers on each node represent the bootstrap value from 1000 replicates and the scale bar represents 0.001 substitutions per nucleotide.

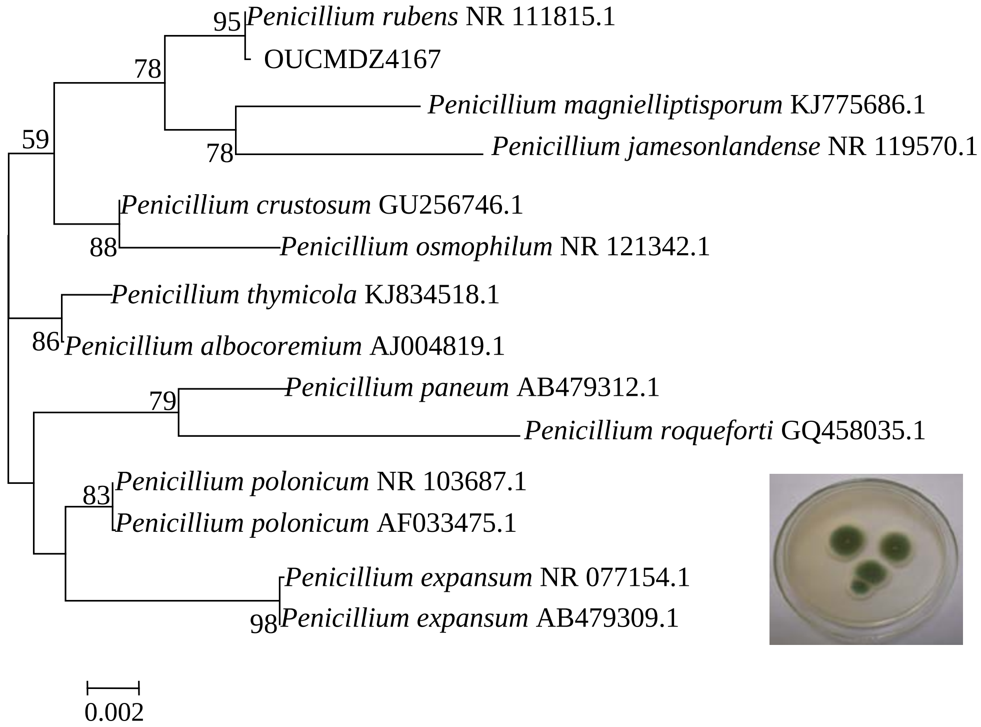

Figure S39. Neighbor-joining of phylogenetic tree of strain OUCMDZ4167 from *E. superba* based on 18S rRNA gene sequences. The numbers on each node represent the bootstrap value from 1000 replicates and the scale bar represents 0.002 substitutions per nucleotide.

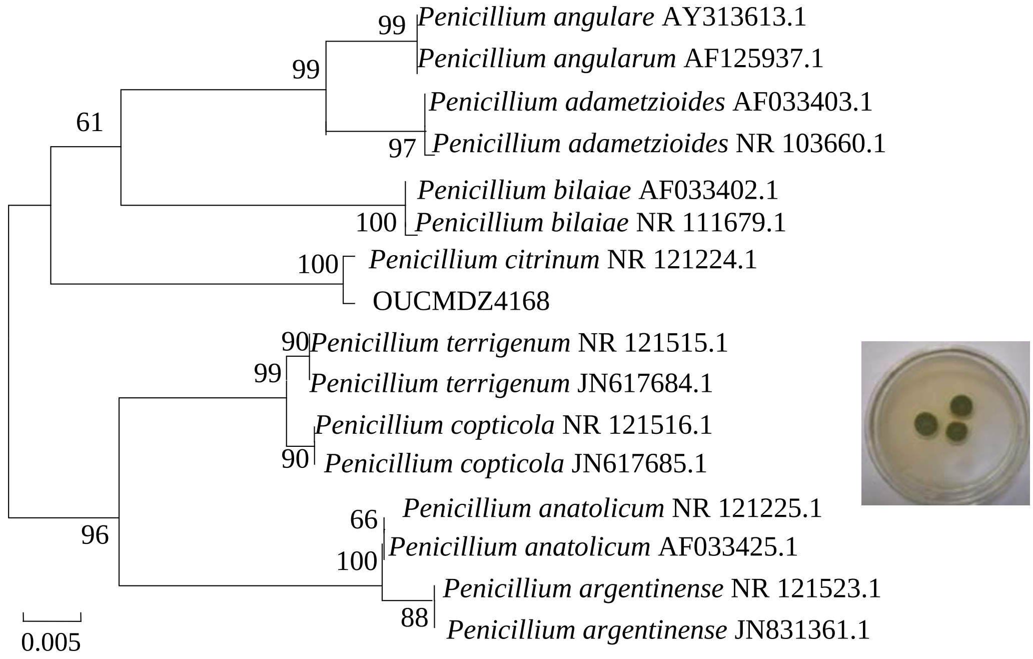

Figure S40. Neighbor-joining of phylogenetic tree of strain OUCMDZ4168 from *E. superba* based on 18S rRNA gene sequences. The numbers on each node represent the bootstrap value from 1000 replicates and the scale bar represents 0.005 substitutions per nucleotide.

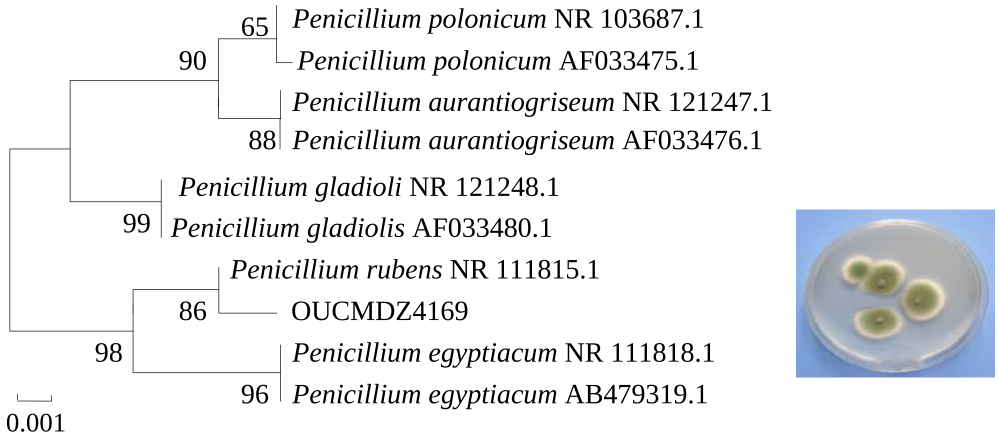

Figure S41. Neighbor-joining of phylogenetic tree of strain OUCMDZ4169 from *E. superba* based on 18S rRNA gene sequences. The numbers on each node represent the bootstrap value from 1000 replicates and the scale bar represents 0.001 substitutions per nucleotide.

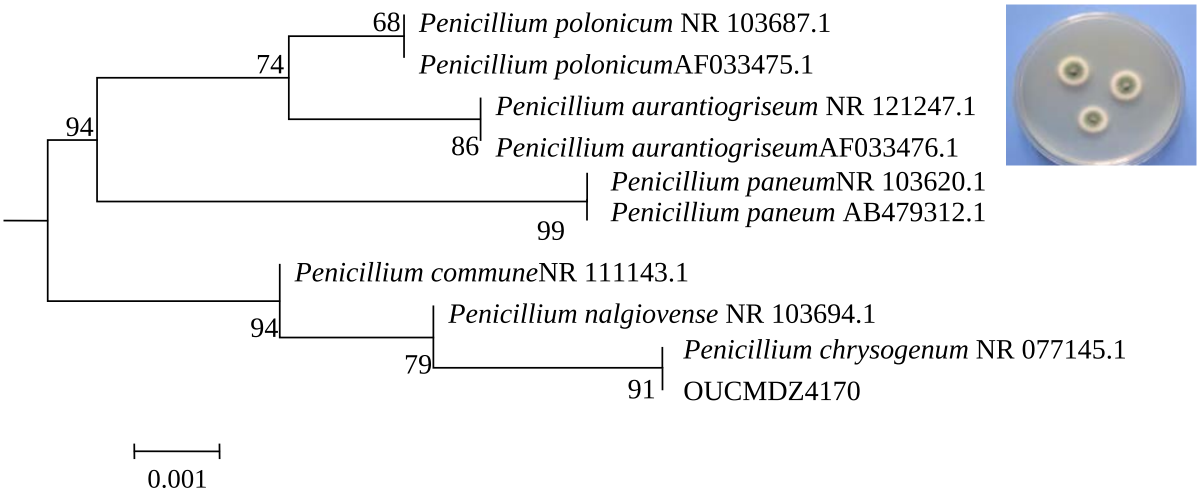

Figure S42. Neighbor-joining of phylogenetic tree of strain OUCMDZ4170 from *E. superba* based on 18S rRNA gene sequences. The numbers on each node represent the bootstrap value from 1000 replicates and the scale bar represents 0.001 substitutions per nucleotide.

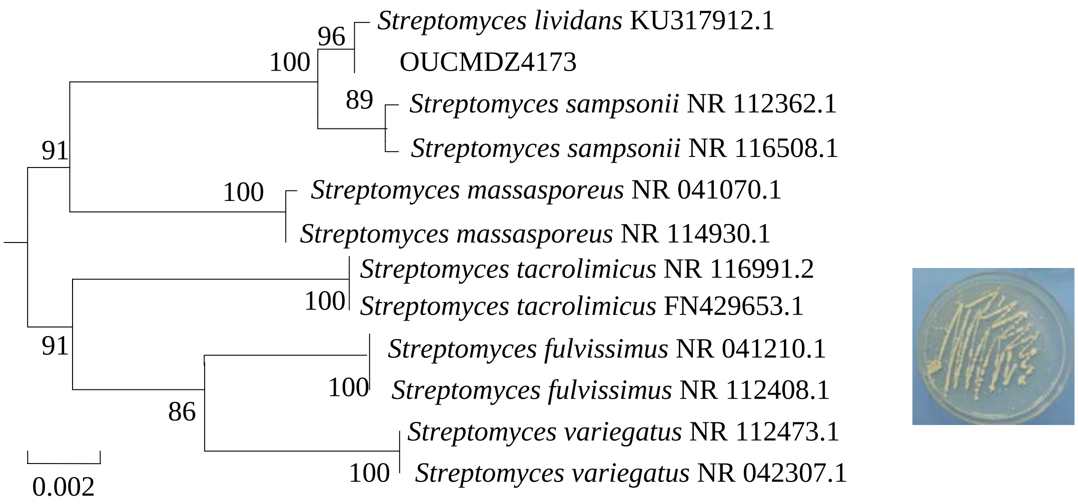

Figure S43. Neighbor-joining of phylogenetic tree of strain OUCMDZ4173 from *E. superba* based on 16S rRNA gene sequences. The numbers on each node represent the bootstrap value from 1000 replicates and the scale bar represents 0.002 substitutions per nucleotide.

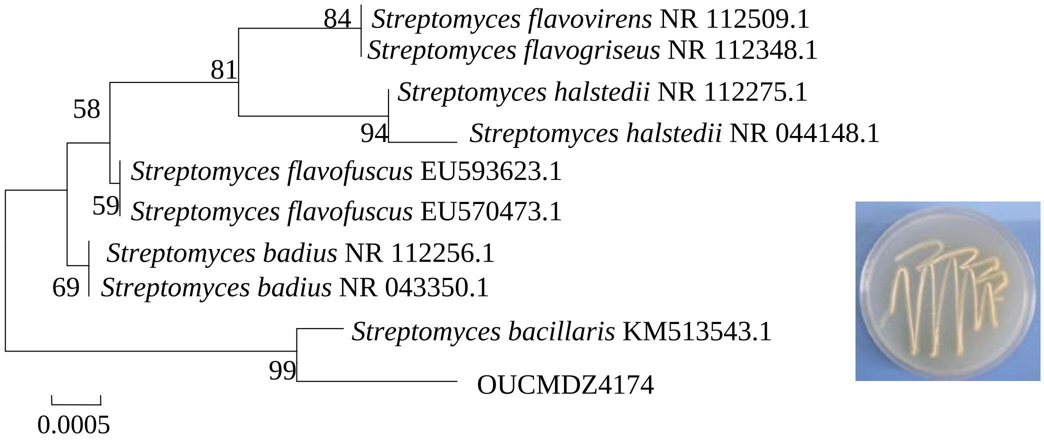

Figure S44. Neighbor-joining of phylogenetic tree of strain OUCMDZ4174 from *E. superba* based on 16S rRNA gene sequences. The numbers on each node represent the bootstrap value from 1000 replicates and the scale bar represents 0.0005 substitutions per nucleotide.

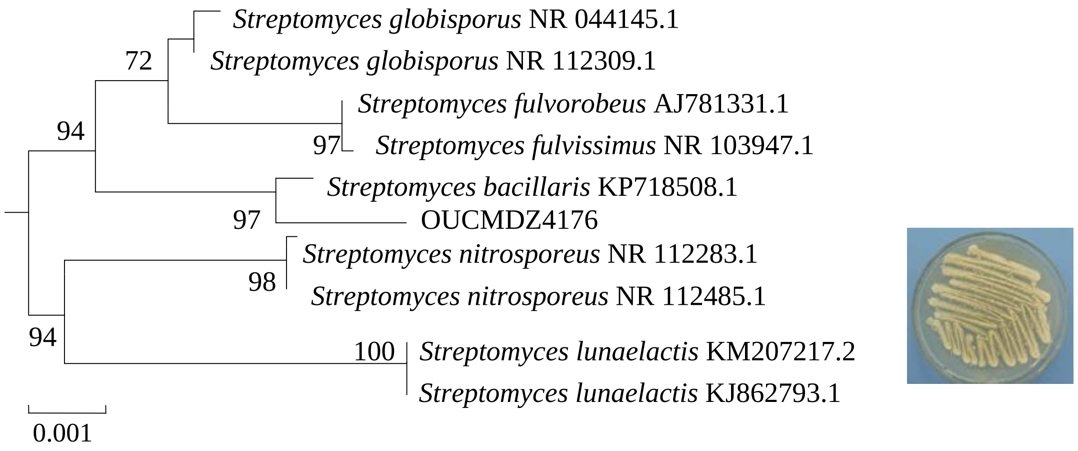

Figure S45. Neighbor-joining of phylogenetic tree of strain OUCMDZ4176 from *E. superba* based on 16S rRNA gene sequences. The numbers on each node represent the bootstrap value from 1000 replicates and the scale bar represents 0.001 substitutions per nucleotide.

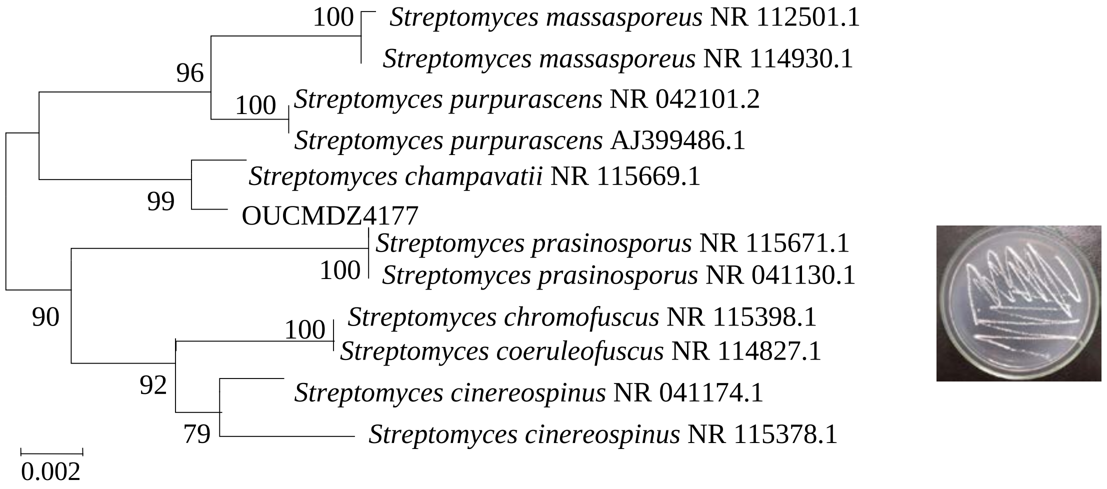

Figure S46. Neighbor-joining of phylogenetic tree of strain OUCMDZ4177 from *E. superba* based on 16S rRNA gene sequences. The numbers on each node represent the bootstrap value from 1000 replicates and the scale bar represents 0.002 substitutions per nucleotide.

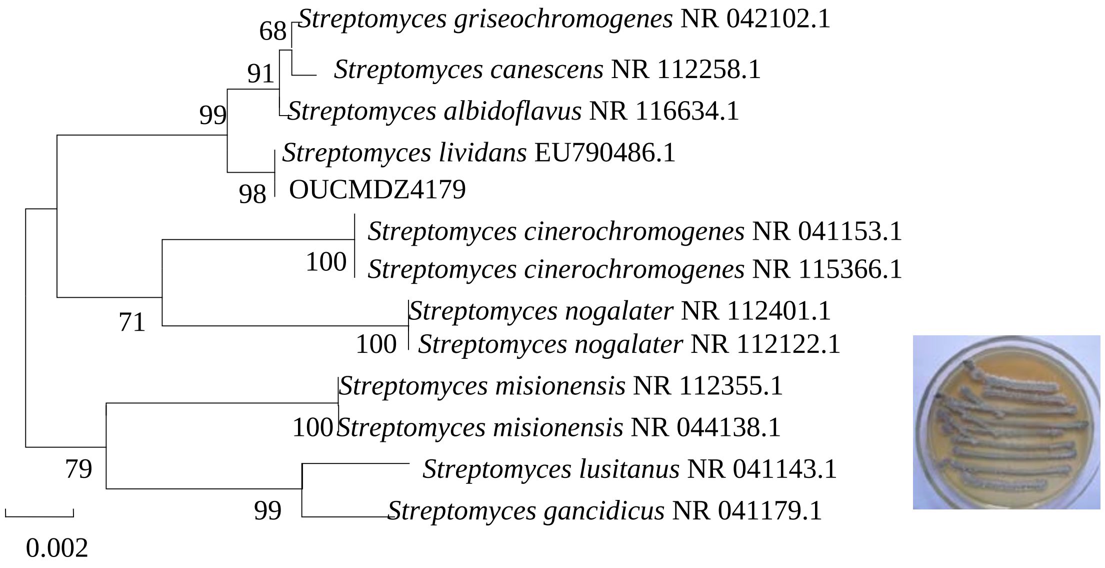

Figure S47. Neighbor-joining of phylogenetic tree of strain OUCMDZ4179 from *E. superba* based on 16S rRNA gene sequences. The numbers on each node represent the bootstrap value from 1000 replicates and the scale bar represents 0.002 substitutions per nucleotide.

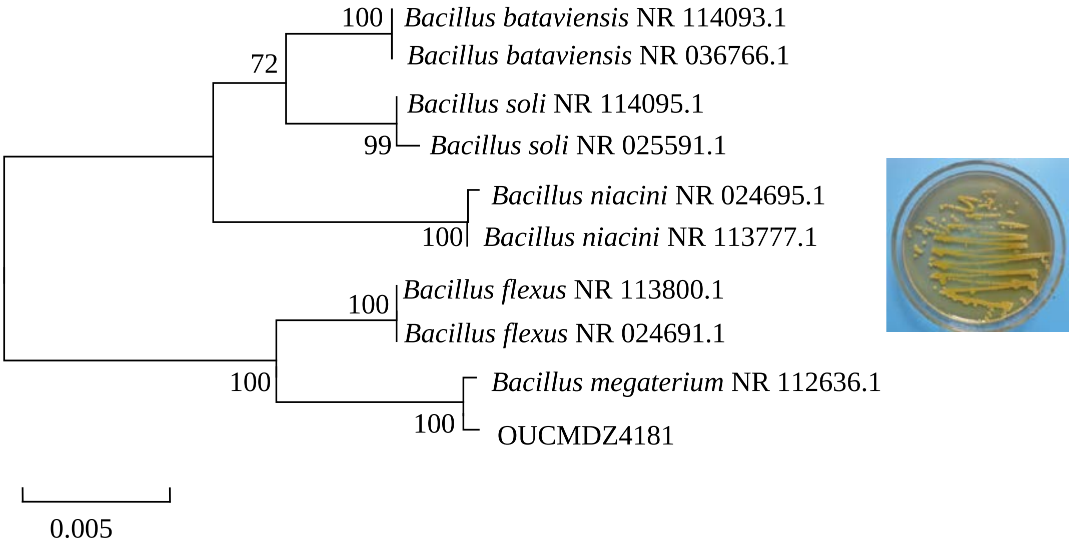

Figure S48. Neighbor-joining of phylogenetic tree of strain OUCMDZ4181 from *E. superba* based on 16S rRNA gene sequences. The numbers on each node represent the bootstrap value from 1000 replicates and the scale bar represents 0.005 substitutions per nucleotide.

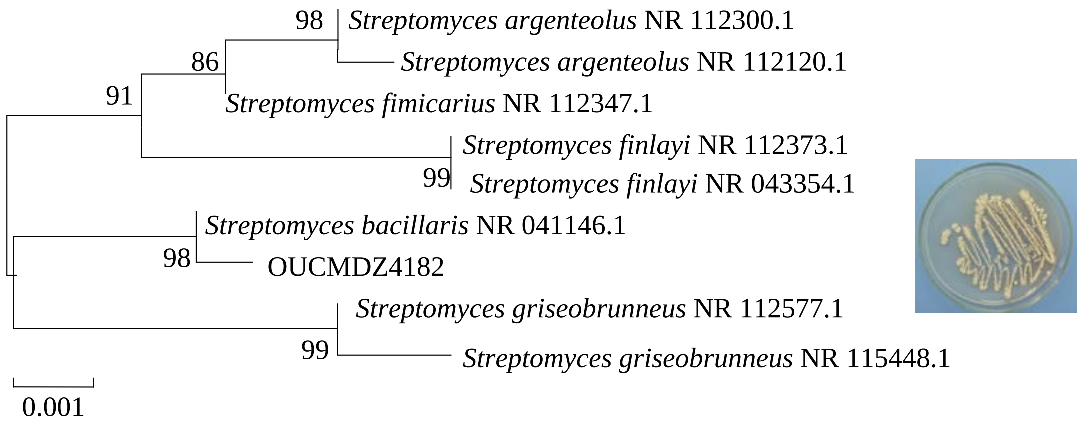

Figure S49. Neighbor-joining of phylogenetic tree of strain OUCMDZ4182 from *E. superba* based on 16S rRNA gene sequences. The numbers on each node represent the bootstrap value from 1000 replicates and the scale bar represents 0.001 substitutions per nucleotide.

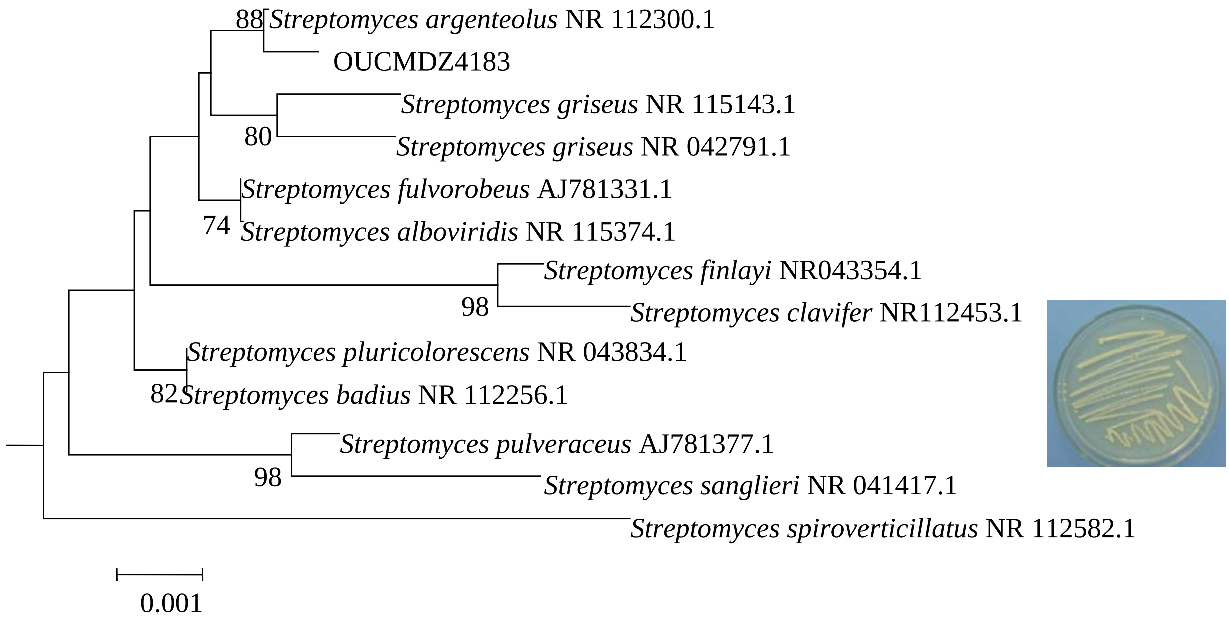

Figure S50. Neighbor-joining of phylogenetic tree of strain OUCMDZ4183 from *E. superba* based on 16S rRNA gene sequences. The numbers on each node represent the bootstrap value from 1000 replicates and the scale bar represents 0.001 substitutions per nucleotide.

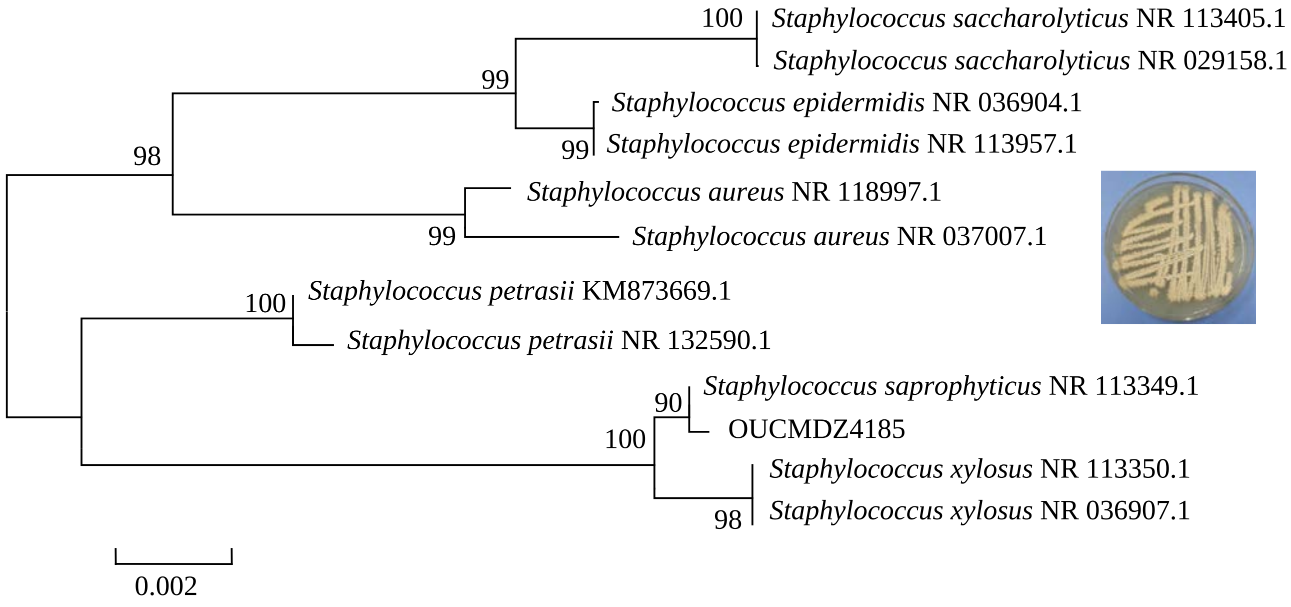

Figure S51. Neighbor-joining of phylogenetic tree of strain OUCMDZ4185 from *E. superba* based on 16S rRNA gene sequences. The numbers on each node represent the bootstrap value from 1000 replicates and the scale bar represents 0.002 substitutions per nucleotide.

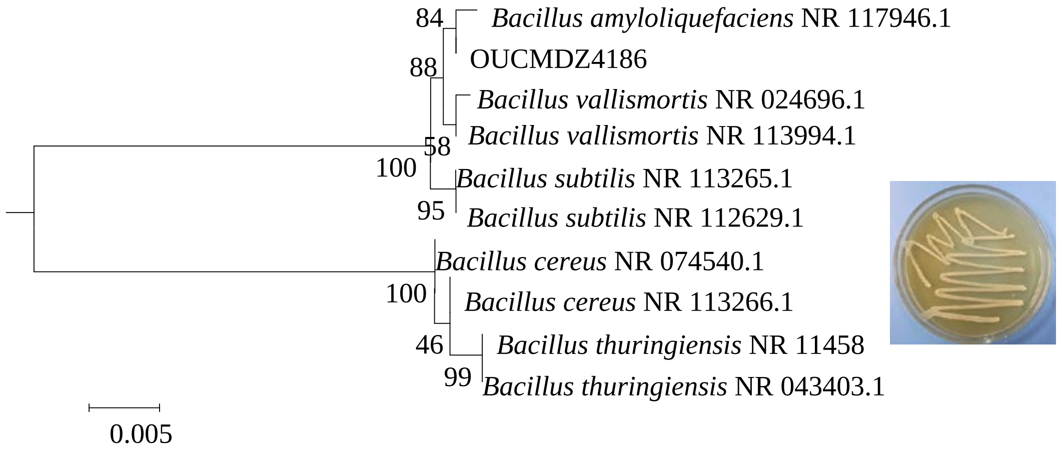

Figure S52. Neighbor-joining of phylogenetic tree of strain OUCMDZ4186 from *E. superba* based on 16S rRNA gene sequences. The numbers on each node represent the bootstrap value from 1000 replicates and the scale bar represents 0.005 substitutions per nucleotide.

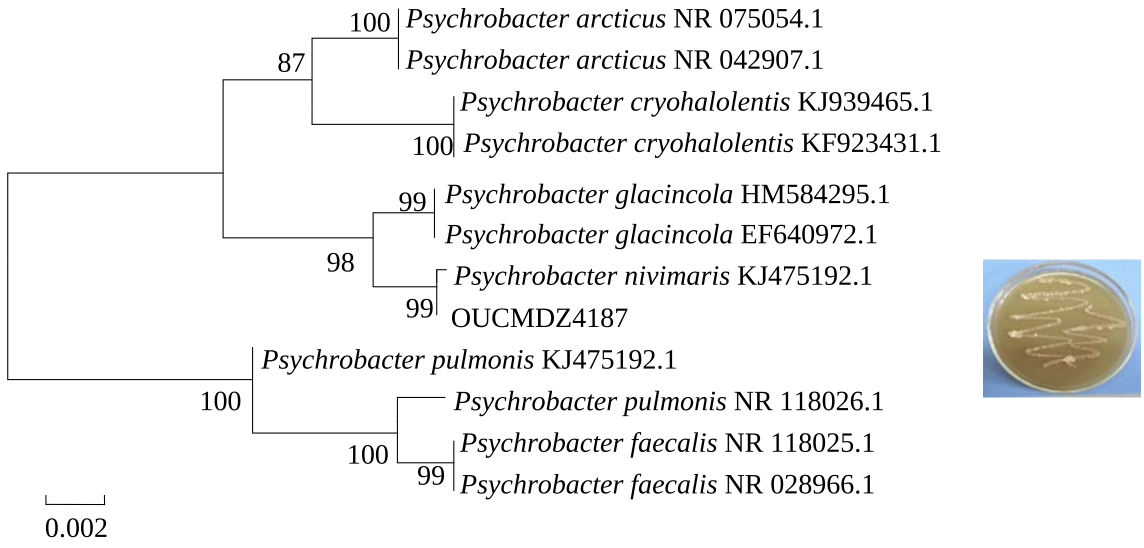

Figure S53. Neighbor-joining of phylogenetic tree of strain OUCMDZ4187 from *E. superba* based on 16S rRNA gene sequences. The numbers on each node represent the bootstrap value from 1000 replicates and the scale bar represents 0.002 substitutions per nucleotide.

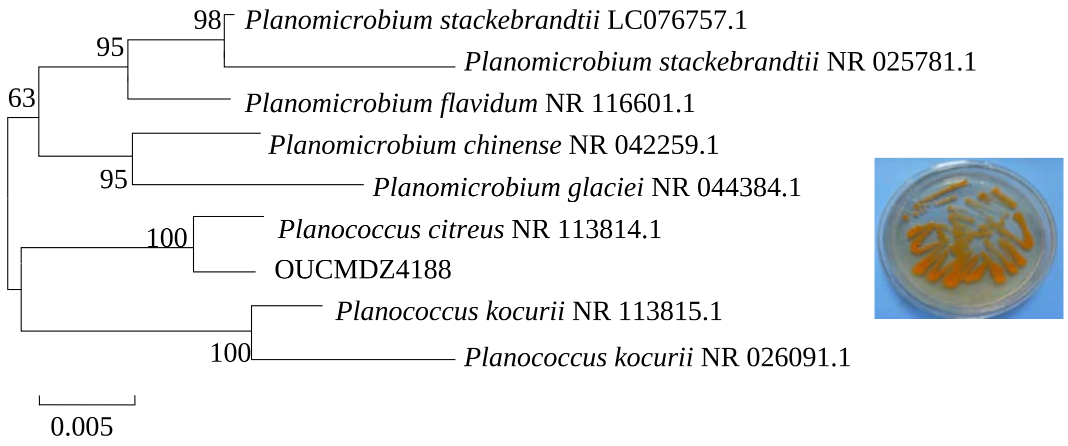

Figure S54. Neighbor-joining of phylogenetic tree of strain OUCMDZ4188 from *E. superba* based on 16S rRNA gene sequences. The numbers on each node represent the bootstrap value from 1000 replicates and the scale bar represents 0.005 substitutions per nucleotide.

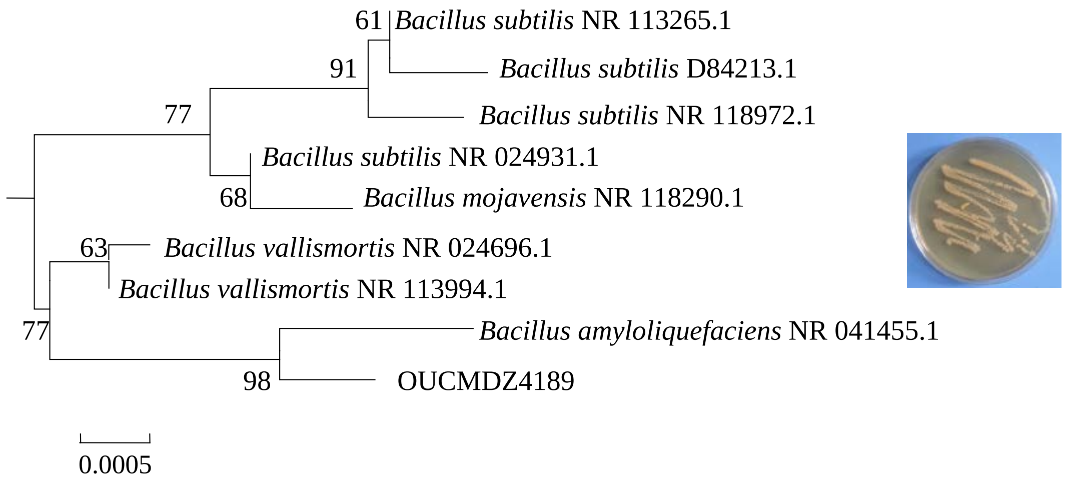

Figure S55. Neighbor-joining of phylogenetic tree of strain OUCMDZ4189 from *E. superba* based on 16S rRNA gene sequences. The numbers on each node represent the bootstrap value from 1000 replicates and the scale bar represents 0.0005 substitutions per nucleotide.

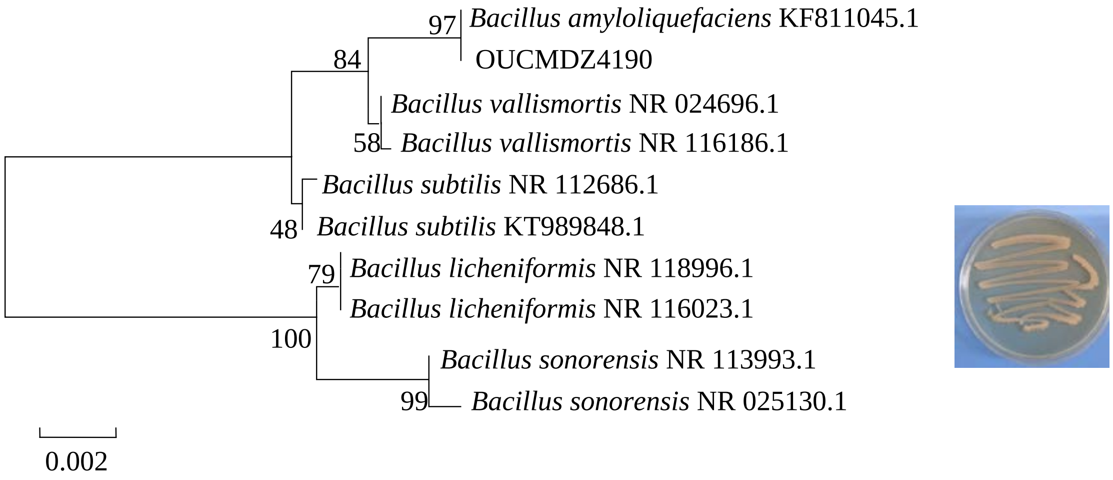

Figure S56. Neighbor-joining of phylogenetic tree of strain OUCMDZ4190 from *E. superba* based on 16S rRNA gene sequences. The numbers on each node represent the bootstrap value from 1000 replicates and the scale bar represents 0.002 substitutions per nucleotide.

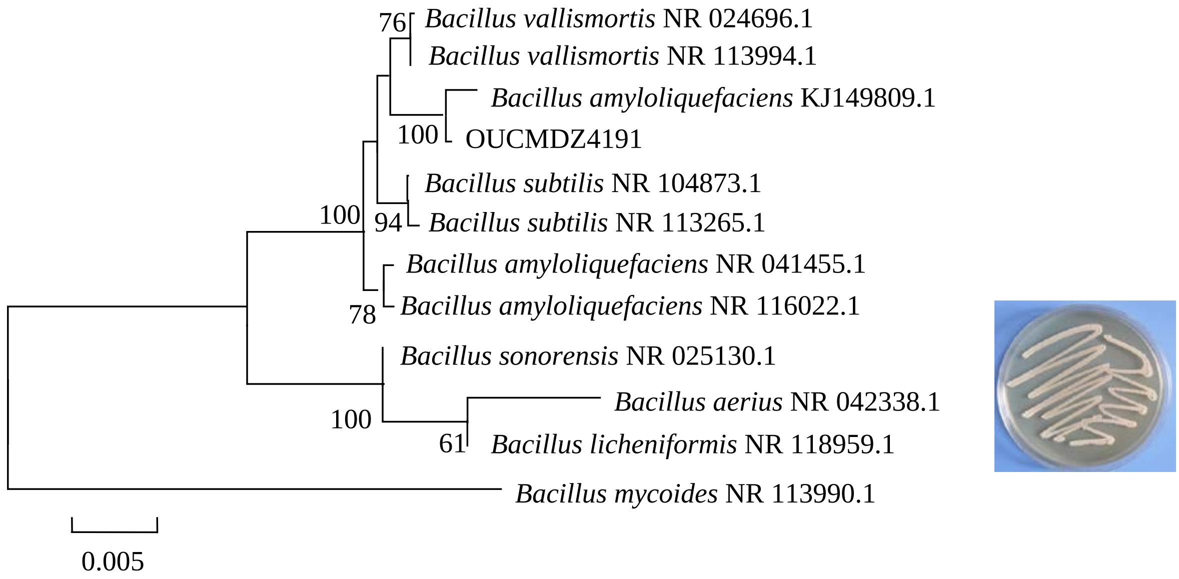

Figure S57. Neighbor-joining of phylogenetic tree of strain OUCMDZ4191 from *E. superba* based on 16S rRNA gene sequences. The numbers on each node represent the bootstrap value from 1000 replicates and the scale bar represents 0.005 substitutions per nucleotide.

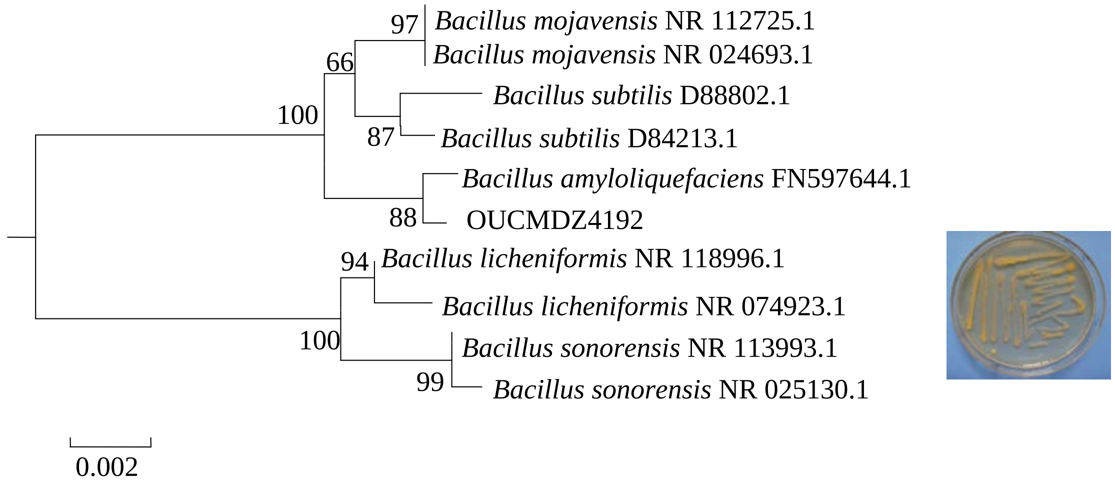

Figure S58. Neighbor-joining of phylogenetic tree of strain OUCMDZ4192 from *E. superba* based on 16S rRNA gene sequences. The numbers on each node represent the bootstrap value from 1000 replicates and the scale bar represents 0.002 substitutions per nucleotide.

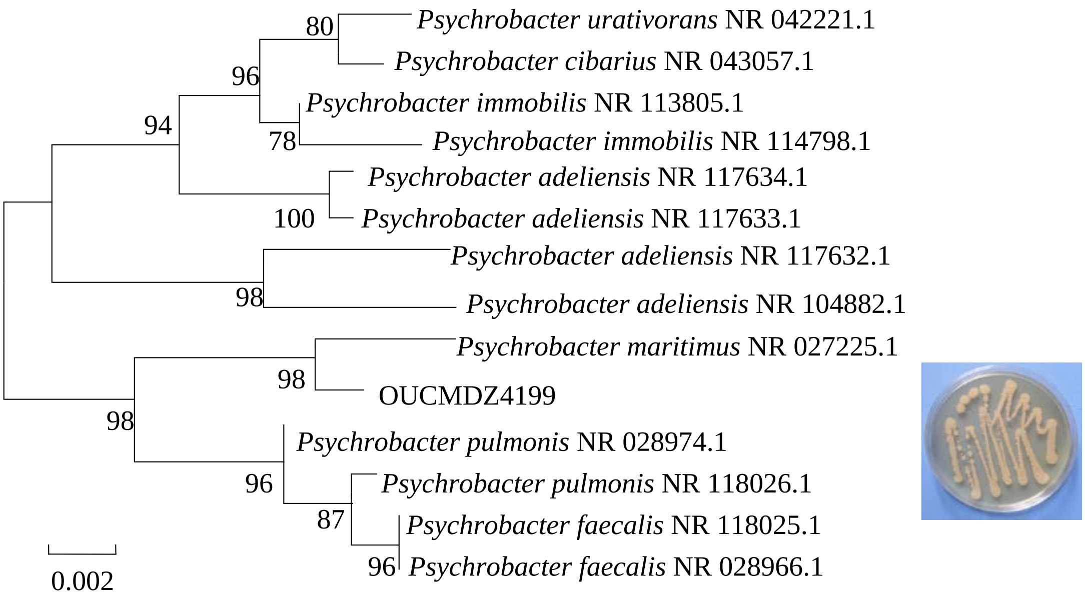

Figure S59. Neighbor-joining of phylogenetic tree of strain OUCMDZ4199 from *E. superba* based on 16S rRNA gene sequences. The numbers on each node represent the bootstrap value from 1000 replicates and the scale bar represents 0.002 substitutions per nucleotide.

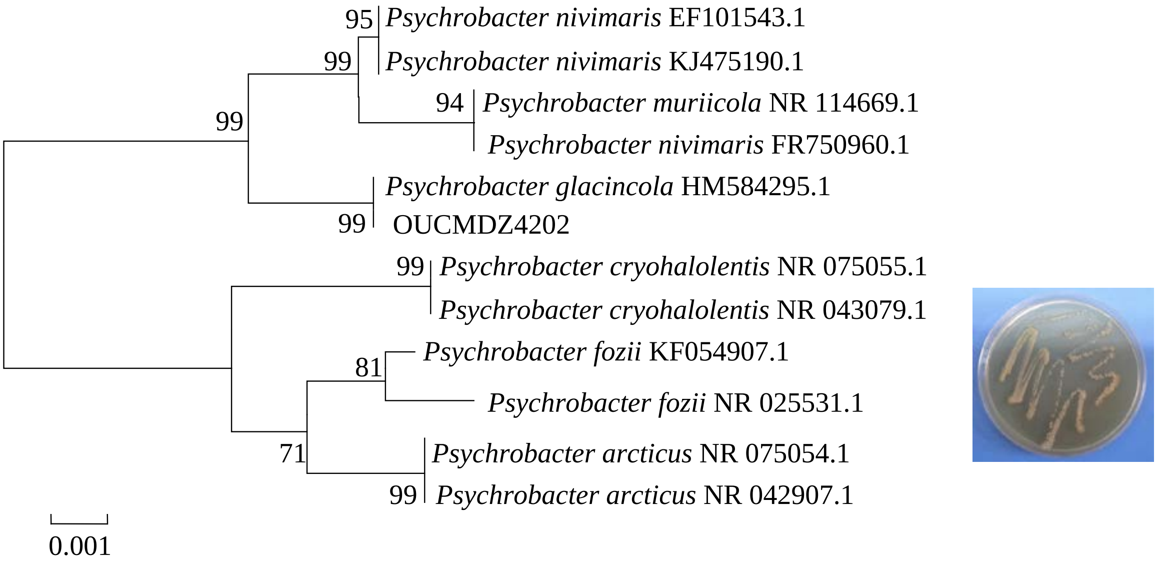

Figure S60. Neighbor-joining of phylogenetic tree of strain OUCMDZ4202 from *E. superba* based on 16S rRNA gene sequences. The numbers on each node represent the bootstrap value from 1000 replicates and the scale bar represents 0.001 substitutions per nucleotide.

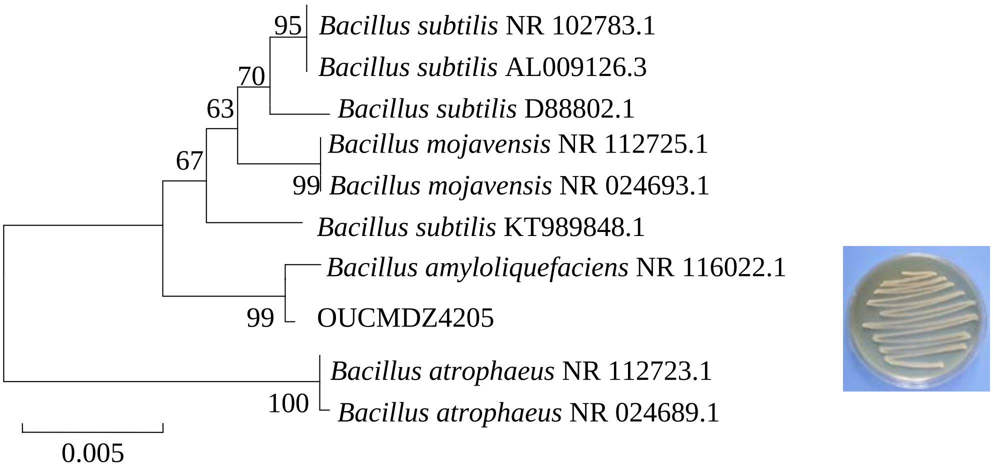

Figure S61. Neighbor-joining of phylogenetic tree of strain OUCMDZ4205 from *E. superba* based on 16S rRNA gene sequences. The numbers on each node represent the bootstrap value from 1000 replicates and the scale bar represents 0.005 substitutions per nucleotide.

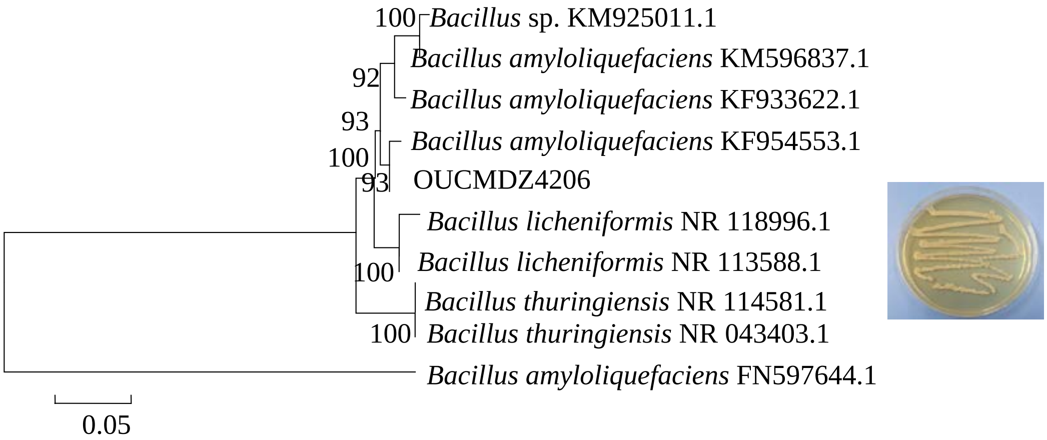

Figure S62. Neighbor-joining of phylogenetic tree of strain OUCMDZ4206 from *E. superba* based on 16S rRNA gene sequences. The numbers on each node represent the bootstrap value from 1000 replicates and the scale bar represents 0.05 substitutions per nucleotide.

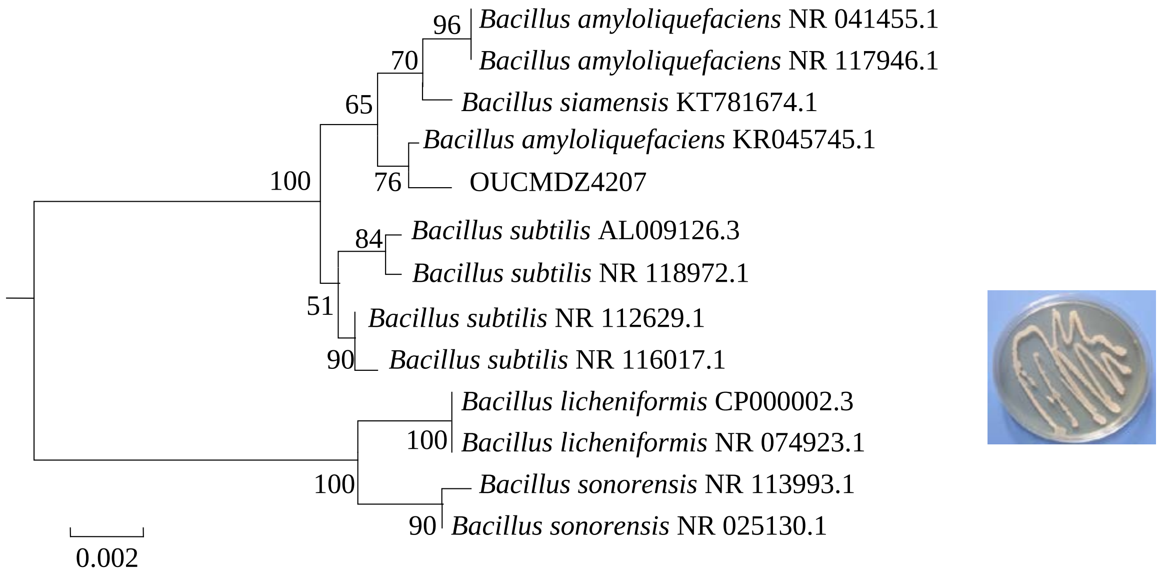

Figure S63. Neighbor-joining of phylogenetic tree of strain OUCMDZ4207 from *E. superba* based on 16S rRNA gene sequences. The numbers on each node represent the bootstrap value from 1000 replicates and the scale bar represents 0.002 substitutions per nucleotide.

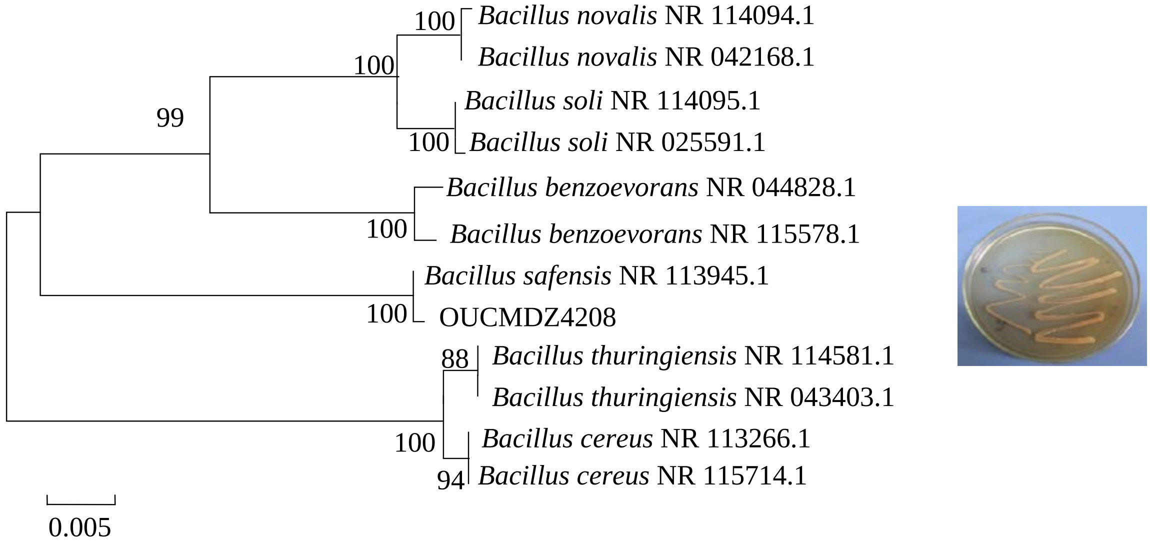

Figure S64. Neighbor-joining of phylogenetic tree of strain OUCMDZ4208 from *E. superba* based on 16S rRNA gene sequences. The numbers on each node represent the bootstrap value from 1000 replicates and the scale bar represents 0.005 substitutions per nucleotide.

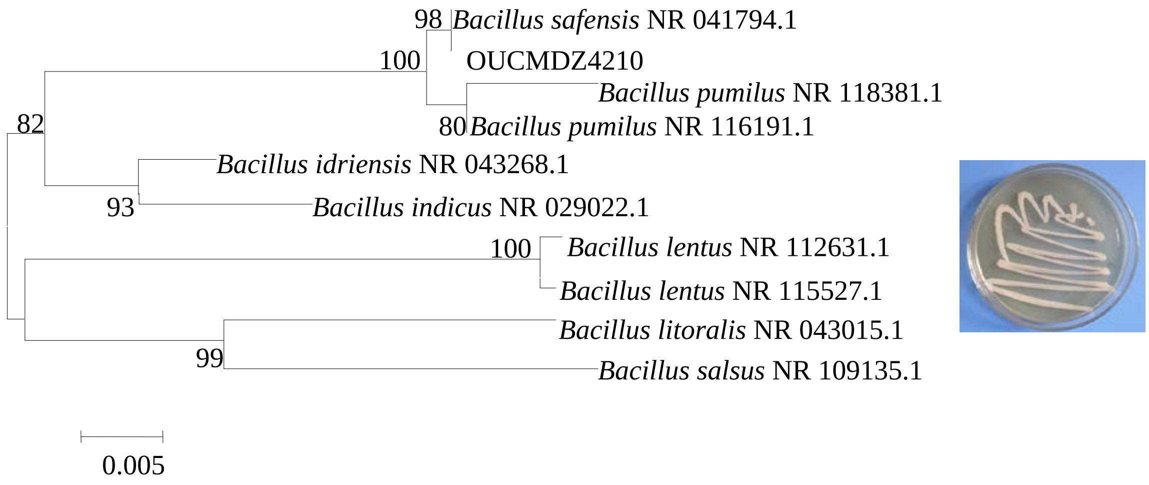

Figure S65. Neighbor-joining of phylogenetic tree of strain OUCMDZ4210 from *E. superba* based on 16S rRNA gene sequences. The numbers on each node represent the bootstrap value from 1000 replicates and the scale bar represents 0.005 substitutions per nucleotide.

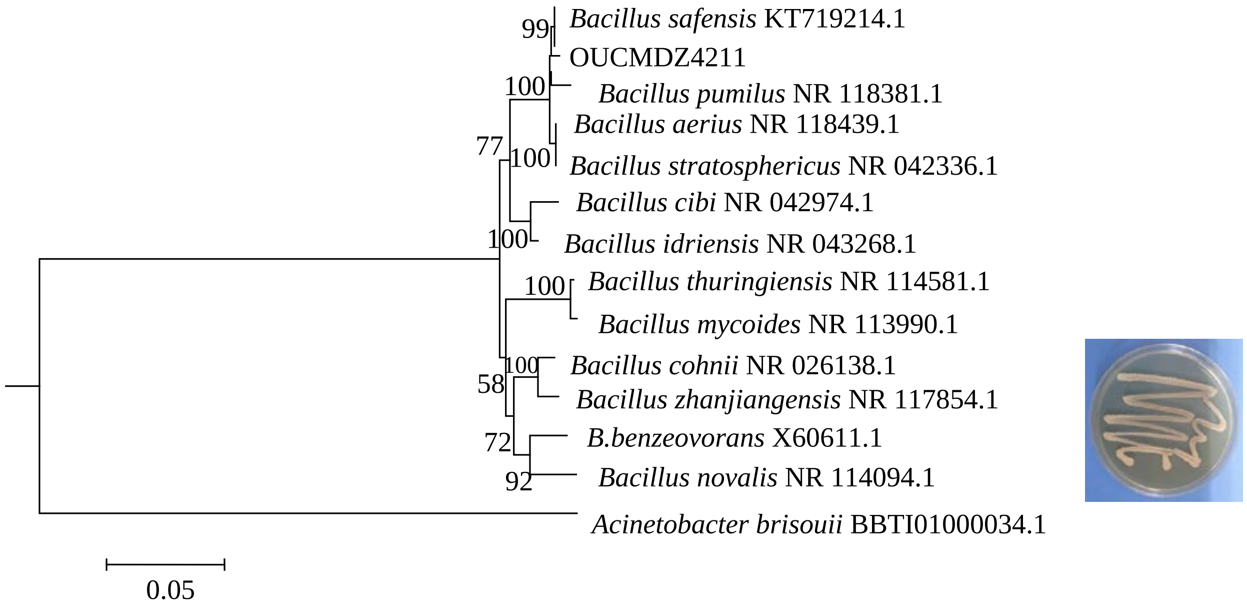

Figure S66. Neighbor-joining of phylogenetic tree of strain OUCMDZ4211 from *E. superba* based on 16S rRNA gene sequences. The numbers on each node represent the bootstrap value from 1000 replicates and the scale bar represents 0.05 substitutions per nucleotide.

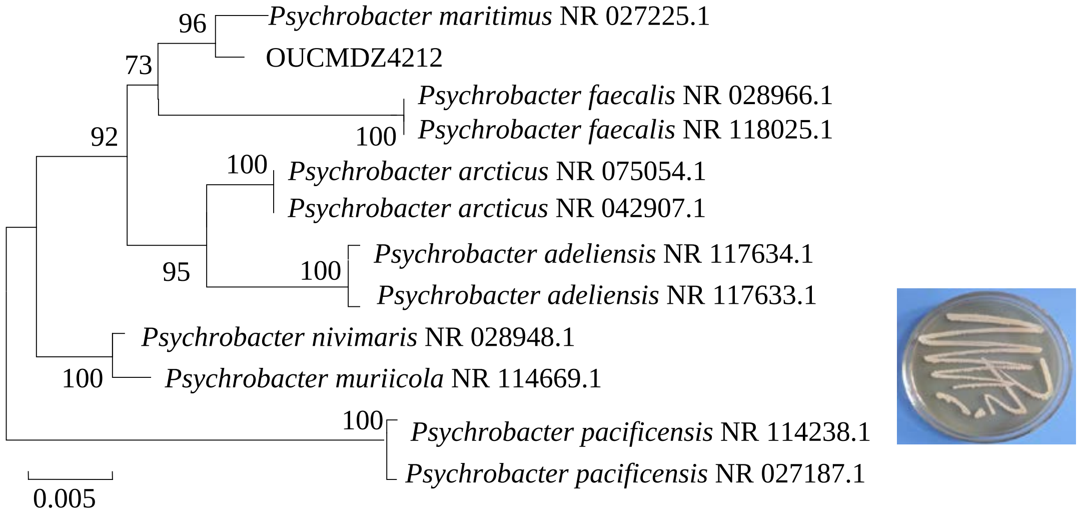

Figure S67. Neighbor-joining of phylogenetic tree of strain OUCMDZ4212 from *E. superba* based on 16S rRNA gene sequences. The numbers on each node represent the bootstrap value from 1000 replicates and the scale bar represents 0.005 substitutions per nucleotide.

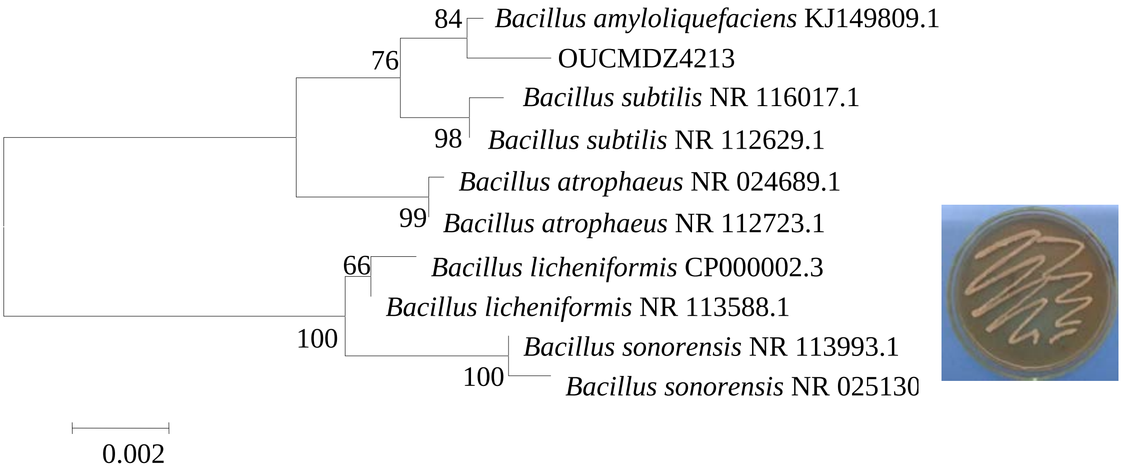

Figure S68. Neighbor-joining of phylogenetic tree of strain OUCMDZ4213 from *E. superba* based on 16S rRNA gene sequences. The numbers on each node represent the bootstrap value from 1000 replicates and the scale bar represents 0.002 substitutions per nucleotide.

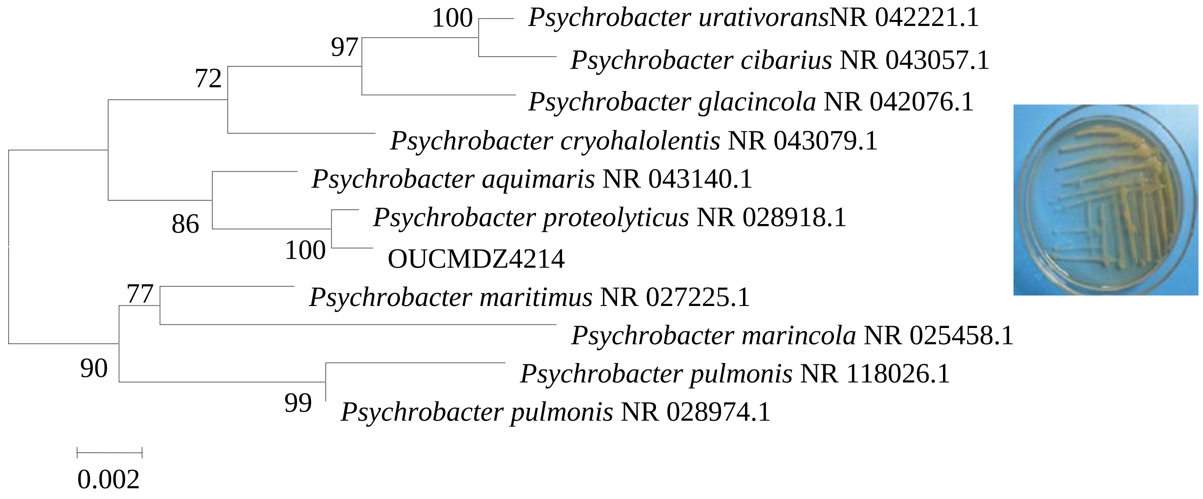

Figure S69. Neighbor-joining of phylogenetic tree of strain OUCMDZ4214 from *E. superba* based on 16S rRNA gene sequences. The numbers on each node represent the bootstrap value from 1000 replicates and the scale bar represents 0.002 substitutions per nucleotide.

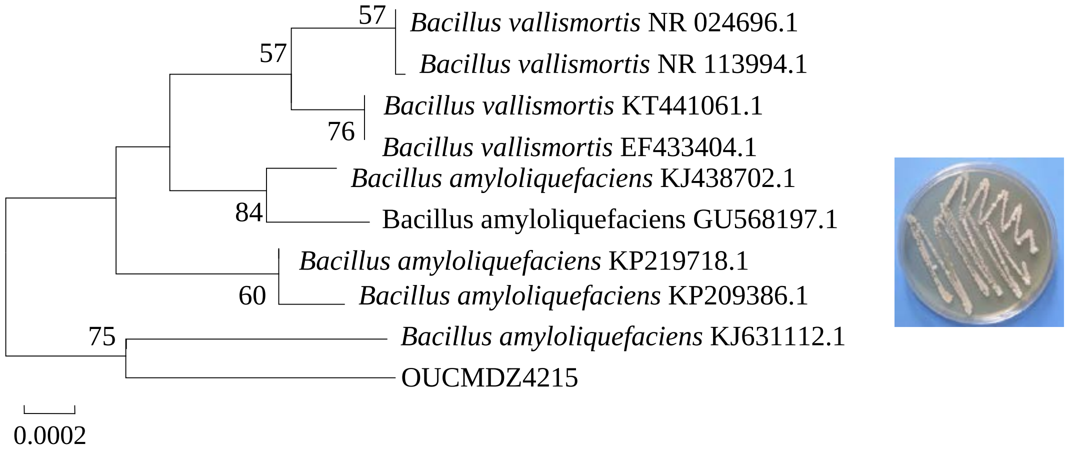

Figure S70. Neighbor-joining of phylogenetic tree of strain OUCMDZ4215 from *E. superba* based on 16S rRNA gene sequences. The numbers on each node represent the bootstrap value from 1000 replicates and the scale bar represents 0.0002 substitutions per nucleotide.

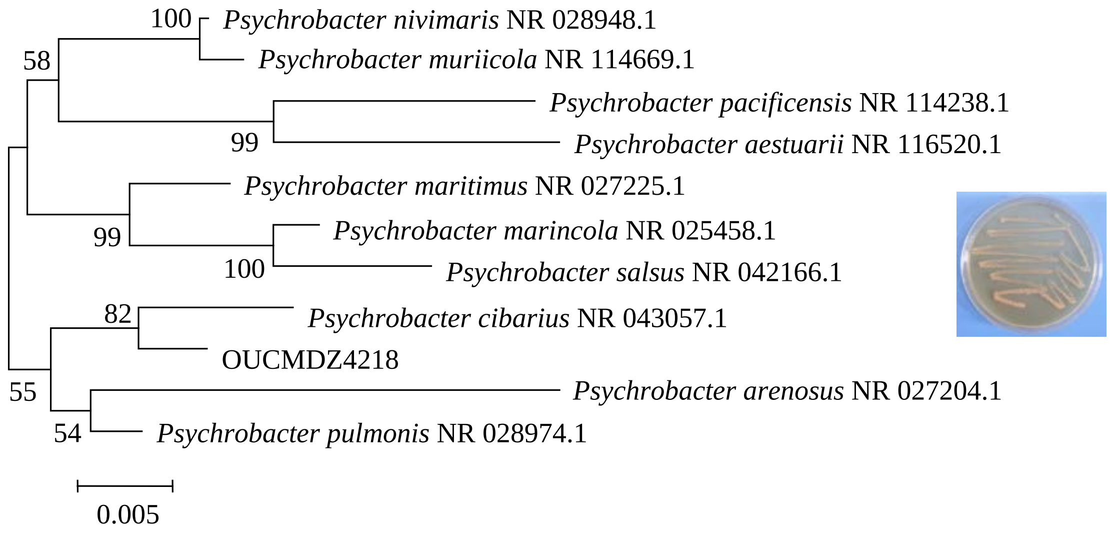

Figure S71. Neighbor-joining of phylogenetic tree of strain OUCMDZ4218 from *E. superba* based on 16S rRNA gene sequences. The numbers on each node represent the bootstrap value from 1000 replicates and the scale bar represents 0.005 substitutions per nucleotide.

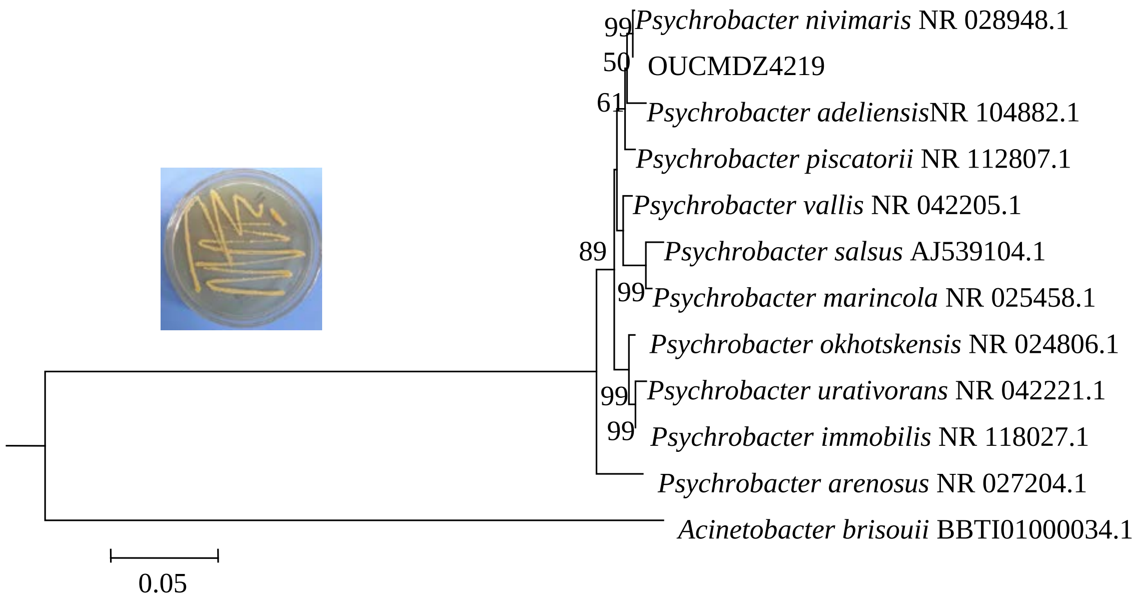

Figure S72. Neighbor-joining of phylogenetic tree of strain OUCMDZ4219 from *E. superba* based on 16S rRNA gene sequences. The numbers on each node represent the bootstrap value from 1000 replicates and the scale bar represents 0.05 substitutions per nucleotide.

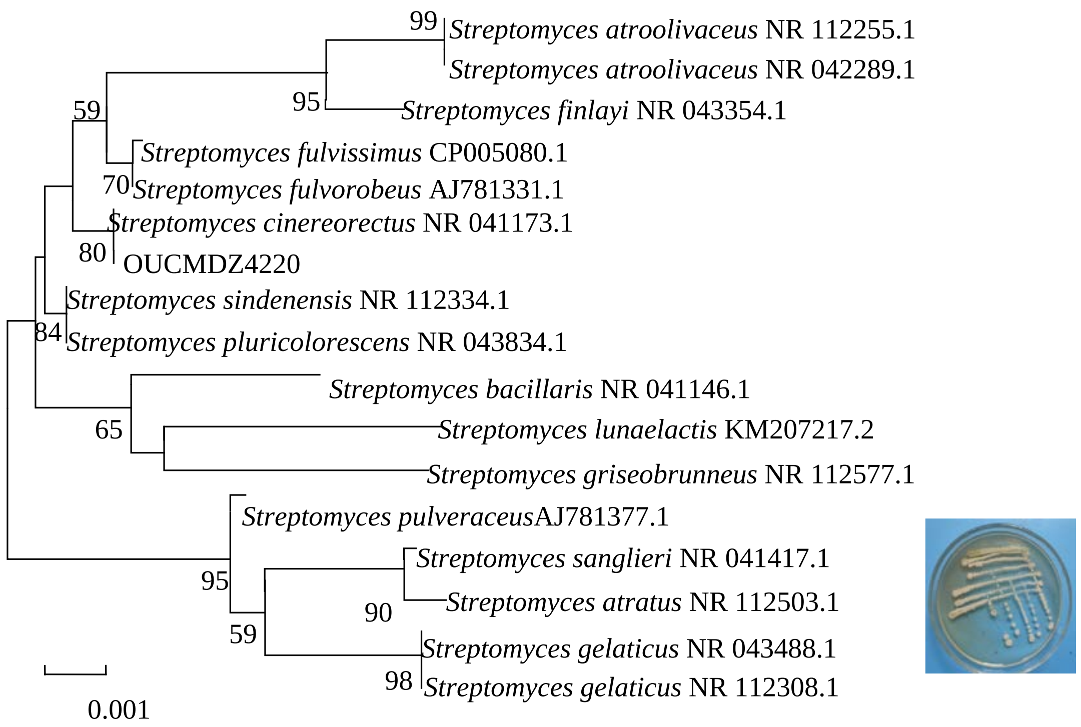

Figure S73. Neighbor-joining of phylogenetic tree of strain OUCMDZ4220 from *E. superba* based on 16S rRNA gene sequences. The numbers on each node represent the bootstrap value from 1000 replicates and the scale bar represents 0.001 substitutions per nucleotide.

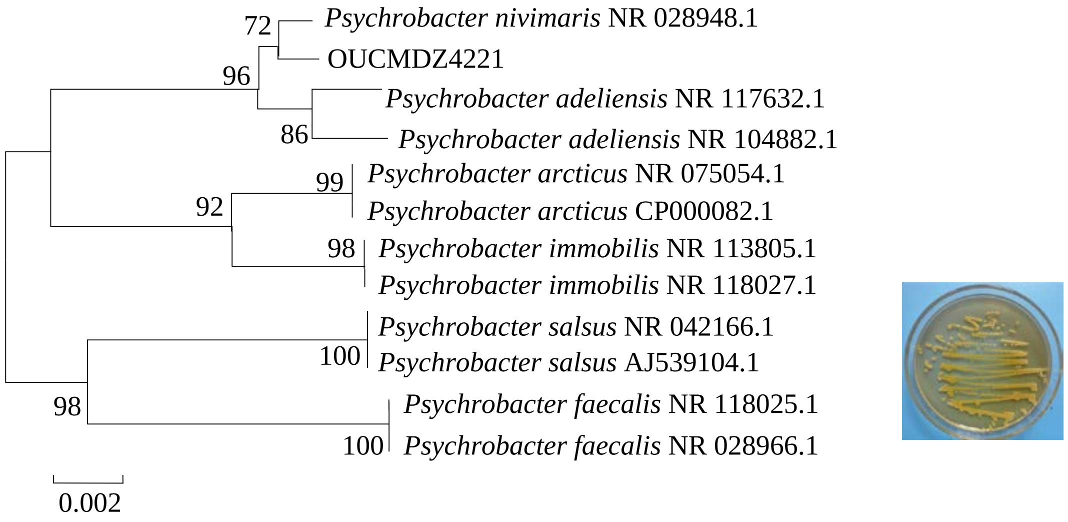

Figure S74. Neighbor-joining of phylogenetic tree of strain OUCMDZ4221 from *E. superba* based on 16S rRNA gene sequences. The numbers on each node represent the bootstrap value from 1000 replicates and the scale bar represents 0.002 substitutions per nucleotide.

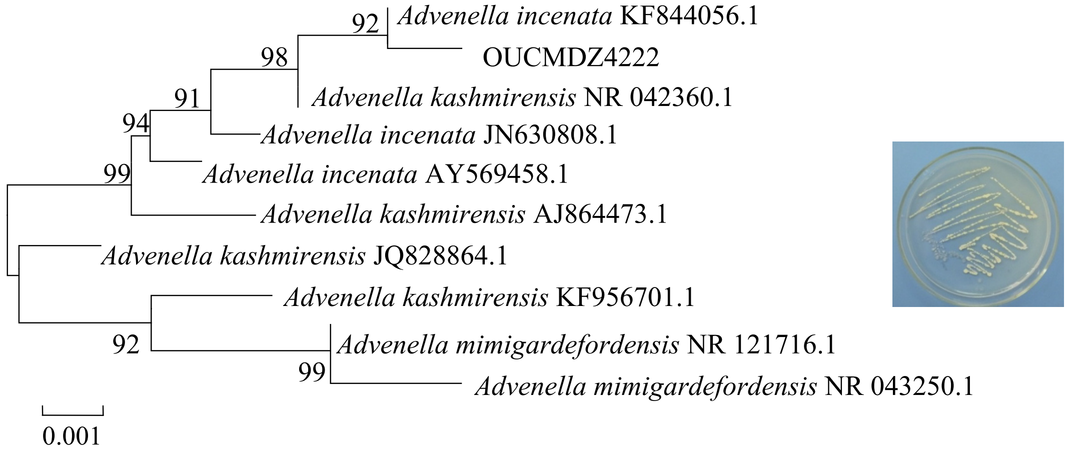

Figure S75. Neighbor-joining of phylogenetic tree of strain OUCMDZ4222 from *E. superba* based on 16S rRNA gene sequences. The numbers on each node represent the bootstrap value from 1000 replicates and the scale bar represents 0.001 substitutions per nucleotide.

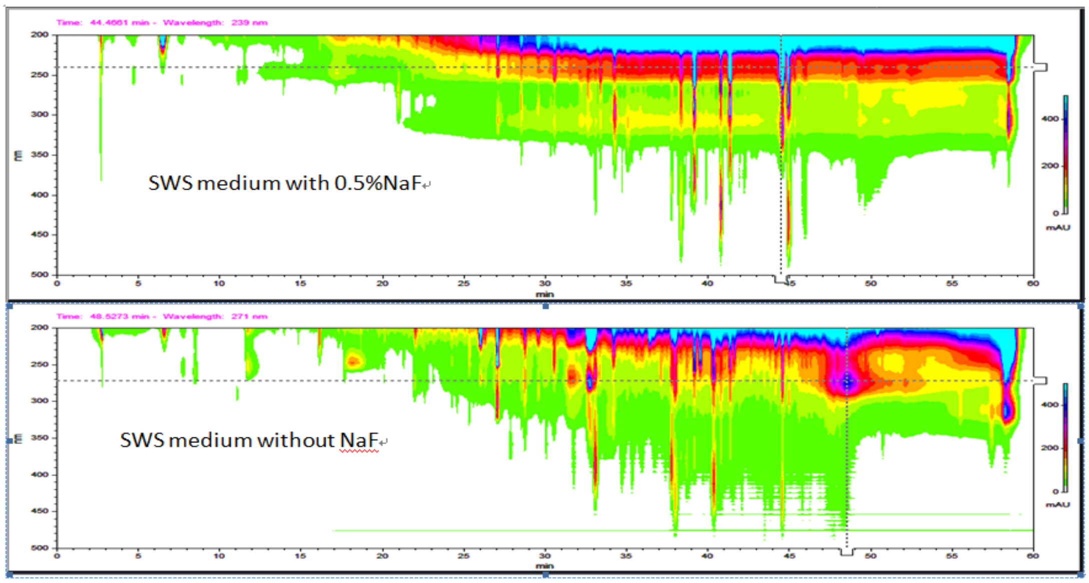

Figure S76. The HPLC profile of the EtOAc extracts from the cultures of *Penicillium citrinum* OUCMDZ4136

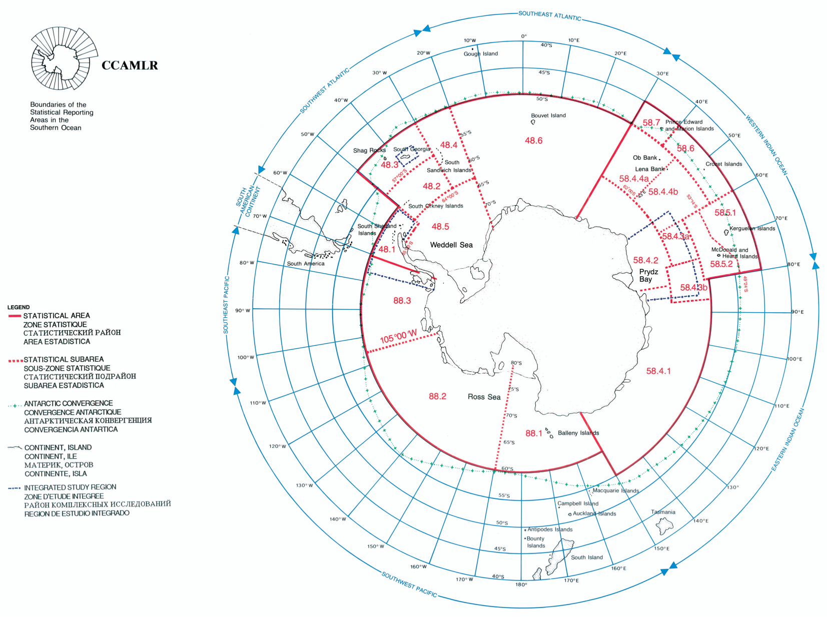

Figure S77. Sampling site (at FAO48.1) of *E. superba* (Picture and license to use the picture from CCAMLR, https://www.ccamlr.org/en/organisation/convention-area)
